# Global burden of disease analysis and projections of ischemic stroke linked to inadequate polyunsaturated fatty acid intake in older women (1990–2021)

**DOI:** 10.3389/fnut.2025.1659895

**Published:** 2025-12-12

**Authors:** Ying Luo, Guoliang Zhu, Quandan Tan, Yapeng Lin, Jie Yang

**Affiliations:** 1 Department of Neurology, Sichuan Provincial People’s Hospital, School of Medicine, University of Electronic Science and Technology of China, Chengdu, China; 2 Laboratory of Neurological Diseases and Brain Function, Department of Neurology, The Affiliated Hospital of Southwest Medical University, Luzhou, China; 3 Department of Neurology, The First Affiliated Hospital of Chengdu Medical College, Chengdu, China

**Keywords:** ischemic stroke, PUFA intake, GBD, elderly women, ASRs, DALYs, dietary risk factors, epidemiological trends

## Abstract

**Background:**

Ischemic stroke (IS), a major global cause of death and disability, is significantly influenced by diet. While polyunsaturated fatty acid (PUFA) exhibits neuroprotective effects, the specific stroke burden attributable to inadequate PUFA intake among women ≥ 50 years remains unquantified. This study aimed to quantify the global IS burden linked to suboptimal PUFA intake in this population and analyze its temporal and sociodemographic trends.

**Methods:**

We estimated ischemic stroke deaths and disability-adjusted life years (DALYs) attributable to the global burden of disease (GBD) risk factor “diet low in PUFA” using the GBD 2021 comparative risk assessment, reporting age-standardized rates and counts with 95% uncertainty intervals. Key burden metrics were assessed using age-standardized rates, with temporal trends (estimated annual percentage change), regional/sociodemographic variations, and decomposition analyses performed. Future burden (to 2050) was projected.

**Results:**

From 1990 to 2021, global age-standardized rates (ASRs) for DALYs, deaths, years lived with disability (YLDs), and years of life lost (YLLs) attributable to PUFA-related ischemic stroke in women aged ≥ 50 years declined or remained stable, with EAPCs of −1.32% (DALYs), −1.47% (deaths), +0.07% (YLDs), and −1.61% (YLLs) per year. Absolute counts increased over the same period, a pattern explained by population growth and aging as shown by our decomposition analysis. Older age groups exhibited higher ASRs, and China and India contributed the largest share of the global absolute burden. Across SDI strata, declines were steepest in high-SDI settings, whereas changes were smaller—often flat to slightly decreasing—in low-SDI settings; the rise in absolute burden there reflects population growth and aging rather than higher per-capita risk. The GBD due to IS associated with PUFAs in women over 50 years is expected to continue increasing in the coming decades, with YLDs projected to experience the most rapid growth. External validation (Global Dietary Database 2018, women ≥ 50 years) confirmed a negative association consistent with the main analysis.

**Conclusion:**

Suboptimal PUFA intake accounted for a substantial share of ischemic stroke burden among women aged ≥ 50 years, while age-standardized rates declined, absolute numbers rose with population growth and aging.

## Introduction

Stroke constitutes the second most prevalent etiology of mortality and functional impairment among adults globally, imposing an economic burden of approximately 0.66% of global gross domestic product (1, 2). Among the various types of strokes, ischemic stroke (IS) is the most common, resulting from the obstruction or interruption of blood flow to the brain. IS accounts for up to 80% of all strokes and can be as high as 91% in some high-income countries (3). The prevalence of IS and the associated disability-adjusted life years (DALYs) were estimated to reach approximately 70 million in 2021, with significant regional and national variations (4, 5).

Traditional stroke risk encompasses both alterable and inherent determinants, including hypertension, diabetes, dyslipidemia, cardiovascular disease, physical inactivity, atrial fibrillation, tobacco and alcohol use, alongside non-modifiable factors such as age, sex, and genetic predisposition (4–6). Among these, age is the most significant risk factor, with stroke incidence doubling every decade after 55 years (7). GBD 2021 indicates that ∼85% of incident strokes occur at ≥ 50 years. Mortality and DALY rates rise sharply after 50. Although age-standardized rates have fallen since 1990, absolute deaths and DALYs have risen with population growth and aging—supporting our focus on women ≥ 50 years (8, 9). Recent evidence supports cardiovascular and cerebrovascular benefits of polyunsaturated fatty acids (PUFAs) (10–13). Omega-3 PUFAs, especially docosahexaenoic acid (DHA), are established to exert neuroprotective effects by modulating inflammatory pathways and enhancing endothelial function—mechanisms that collectively contribute to reduced brain injury in stroke (11). According to the GBD 2021 analysis, insufficient dietary intake of omega-6 PUFAs increased by 5.3 % of stroke-related DALYs (9).

Emerging evidence from large-scale epidemiological studies indicates that PUFA deficiency may disproportionately affect postmenopausal women due to hormonal changes influencing lipid metabolism and vascular function, yet comprehensive burden-of-disease analyses focusing on this vulnerable population remain limited (14, 15). The postmenopausal decline in estrogen disrupts lipid metabolism by affecting hepatic lipase activity and cholesterol efflux, thereby increasing cardiovascular susceptibility through elevated LDL-C and triglyceride (16, 17). Recent systematic reviews have demonstrated that PUFAs supplementation may be particularly beneficial in older adults, with sex-specific differences in absorption, metabolism, and cardiovascular outcomes warranting targeted investigation (18, 19).

To date, there have been no comprehensive analyses of the disease burden specifically associated with PUFAs and IS. Furthermore, few studies have examined the influence of broader dietary patterns on stroke burden, with most analyses confined to GBD frameworks (4, 5). The GBD 2021 release includes data on morbidity, mortality, and DALYs for 371 conditions across 204 countries, along with 88 risk factors (20). Using GBD 2021, we estimate the global burden of ischemic stroke attributable to diet low in omega-6 PUFA among women aged ≥ 50 years from 1990 to 2021, quantifying population-attributable deaths and DALYs under the theoretical minimum risk exposure level (TMREL, defined as the exposure distribution that minimizes population risk) framework and describing regional and age-related differences and trends.

## Materials and methods

### Data acquisition and sources

This research evaluates the burden associated with PUFA-related IS by utilizing data from the GBD 2021. The GBD 2021 dataset is openly available via the GHDx Outcomes Tool, which offers access to global and regional health metrics. The analysis specifically examines the absolute numbers and age-standardized rates (ASRs) of mortality, DALYs, years lived with disability (YLDs), and years of life lost (YLLs) attributed to PUFA-associated IS. ASRs per 100,000 population were derived using the following equation (5):


$$A\,S\,R=\frac{\sum_{i=1}^{A}a_{i}\,w_{i}}{\sum_{i=1}^{A}w_{i}}\,\text{X}\,100,000$$

(A: the number of age groups; *ai*: the age-specific rate in the age group; w: population count within the corresponding with age category of the reference population standard).

Age-standardized rates were calculated using the GBD 2019 reference population—a theoretical population structure based on the average age distribution across all GBD locations from 1990 to 2017, weighted by population size. This standard comprises 18 age groups (0–4, 5–9, …, 80–84, 85+ years) with predefined weights that eliminate confounding from differing age structures, ensuring observed variations reflect true epidemiological differences rather than demographic effects. The results are reported as mean values accompanied by 95% uncertainty intervals (UIs), calculated from the 2.5th and 97.5th percentile values obtained through 500 posterior sampling iterations (21). DALYs, encompassing both YLDs and YLLs, offer a comprehensive measure of the burden associated with both fatal and non-fatal conditions. Sociodemographic Index (SDI) metrics were integrated into the analysis to evaluate how socioeconomic determinants influence disease burden patterns. Given that this investigation utilized exclusively open-access datasets, institutional review board approval and participant consent procedures were deemed unnecessary, consistent with recognized protocols for secondary analysis of deidentified population health surveillance data.

### GBD modeling framework

In the GBD study, IS was identified according to the GBD 2021 cause list and mapped from source data using ICD-10 I63–I63.9 and ICD-9 433–434; hemorrhagic stroke (I60–I62) was excluded. Non-fatal IS incidence and prevalence were synthesized with DisMod-MR 2.1 from all available epidemiological inputs (5, 20, 22). Mortality from IS was estimated through the Cause of Death Ensemble Modeling (CODEm) framework, which integrates geospatial relationships and covariate data to produce mortality estimates spanning from 1990 to 2021 across various geographic locations. For deaths attributed to implausible, intermediate, or unspecified stroke causes in vital registration systems, statistical redistribution techniques were employed to ensure accurate cause-of-death attribution (5, 20, 22).

### Risk-factor exposure (GBD)—diet low in PUFA

Diet low in PUFA is defined relative to a TMREL of ∼8–12% of total energy from PUFA, reflecting mainly omega-6 fatty acids from liquid vegetable oils; seafood long-chain omega-3 is modeled as a separate risk factor and is not included in this exposure. Country-, year-, sex- and age-specific PUFA exposures are compiled from national dietary surveys (24-h recalls and FFQs) and FAO food balance sheets, harmonized via GBD spatiotemporal modeling and cross-walk procedures; uncertainty intervals propagate sampling, study heterogeneity, and modeling error. Attributable deaths, YLLs, YLDs and DALYs for ischemic stroke were estimated using the comparative risk assessment with TMREL distributions and corresponding risk–outcome functions (23, 24). Following the GBD 2021 comparative risk assessment framework, all estimates represent attributable burden under a TMREL counterfactual and should not be interpreted as individual-level causal effects.

### Population and global burden analysis

Deaths, DALYs, YLDs, and YLLs attributable to PUFA-associated IS from 1990 to 2021 were evaluated, with results reported along with 95% UIs and estimated annual percentage changes (EAPC). The GBD 2021 study organizes the 204 countries and territories into a hierarchical classification system consisting of 7 super-regions, 21 regions, 54 sub-regions, and five SDI quintiles (25). These seven super-regions comprise: Central Europe, Eastern Europe, and Central Asia; High-income; Latin America and Caribbean; North Africa and Middle East; South Asia; Southeast Asia, East Asia, and Oceania; and Sub-Saharan Africa. The population was stratified into 10 distinct age cohorts, each spanning 5 years.

High-resolution mapping was employed to visualize the global distribution of PUFA-related IS burden, emphasizing regional and sociodemographic disparities. Building on this classification, we then examined temporal trends in age-standardized rates (ASR) of PUFA-related IS across super-regions. Temporal trends in ASR of PUFA-related IS were analyzed using the EAPC, which was computed through a linear regression model of the natural logarithm of ASR against calendar year: y = α + βx + ε, where y represents the natural logarithm of the rate, x corresponds to the calendar year, and ε denotes the error term. In this model, β indicates the trend direction of ASR. The EAPC and its 95% confidence interval (CI) were derived using the formula: 100 × (exp(β) − 1). An increasing trend was identified when both the EAPC and the lower bound of its 95% CI were greater than zero; a decreasing trend was identified when both the EAPC and the upper bound of the CI were less than zero; and a stable trend was indicated when the 95% CI contained zero (21, 26).

### SDI analysis

Building on this geographic framework, we subsequently conducted an SDI analysis. The SDI was utilized to evaluate socioeconomic development, functioning as a composite metric based on the total fertility rate, mean years of education, and lag-distributed per capita income for individuals aged 15 and older. SDI values were normalized on a 0–100 scale. Nations and territories were subsequently categorized into five SDI categories: low, low-middle, middle, high-middle, and high (21). To examine the relationship between SDI and PUFA-related IS burden, disease rates were calculated for each SDI category.

### Decomposition analysis

To understand the influence of population growth, aging, and epidemiological shifts on trends in deaths, DALYs, YLDs, and YLLs, we applied decomposition analysis. This approach offers valuable insights into how demographic changes and shifts in health systems contribute to disease burden. Our decomposition methodology incorporates established analytical principles from GBD research, systematically evaluating the relative contributions of demographic transitions and epidemiological factors to observed changes in health outcomes.

### Statistical analysis

Statistical computations were performed utilizing R software (version 4.3.3). Primary outcomes were ASRs and EAPC. Absolute counts are shown for context and interpreted using the decomposition separating demographic (population growth and aging) from epidemiologic change. Absolute DALYs, YLLs, and YLDs are reported in years, and the corresponding rates [age-standardized death rates (ASDRs), ASYLLR (age-standardized YLL rate), ASYLDR (age-standardized YLD rate)] per 100,000, each with 95% UI. Specialized programming code was developed to execute decomposition procedures and sensitivity evaluations. Bayesian statistical modeling was implemented through WinBUGS software (version 1.4). For geographic and spatial analysis, ArcGIS Pro and QGIS 3.16 were used to generate high-resolution maps that illustrate disease burden and disparities related to diet-associated PUFA. Data visualizations, including biaxial and bilateral plots, were produced using the “ggplot2” and “Benchmarking” packages in R. Uncertainty is represented by 95% uncertainty intervals derived from 500 posterior draws per GBD protocol. We report ASRs per 100,000 to two decimals and EAPC as % per year to two decimals with 95% CIs. Each rate is accompanied by the corresponding absolute count for the same years with 95% UIs; absolute counts ≥ 1,000 are rounded to the nearest 1,000, and values < 1,000 are exact. Absolute counts are reported in thousands unless otherwise stated. This study uses the GBD 2021 comparative risk assessment: exposure to diet low in PUFA is mapped to a TMREL, population-attributable fractions (PAFs) are derived from exposure–risk functions, and PAFs are applied to ischemic-stroke deaths and DALYs to yield PUFA-attributable rates and counts with 95% uncertainty intervals. To contextualize the primary population without changing scope, we added global, 2021, descriptive contrasts: women ≥ 50 vs. men ≥ 50 and women ≥ 50 vs. women < 50 for deaths, DALYs, YLLs, and YLDs attributable to diet low in PUFA. We applied a three-factor Das Gupta decomposition (growth, aging, rate change) to the 1990–2021 change in counts; percent contributions with 95% UIs appear in Supplementary Tables 1–4.

### Predictive analytics and projections

To guide public health policy and resource allocation, we projected global trends in deaths, DALYs, YLLs, and YLDs attributable to PUFA-associated ischemic stroke from 2022 to 2050. We used a dual-model approach, autoregressive integrated moving average (ARIMA) and parsimonious error-correction (EC) specifications, to generate complementary forecasts. Candidate models were selected using standard statistical criteria and validated on a hold-out period (2018–2021). Forecasts are shown with 80%/95% prediction intervals. Complete technical specifications are provided in Supplementary Section 1.

## Results

### Analysis of the current status of the burden of disease for IS attributed to insufficient intake of PUFAs and the trend of change from 1990 to 2021 global burden and trends (1990–2021)

**FIGURE 1:**
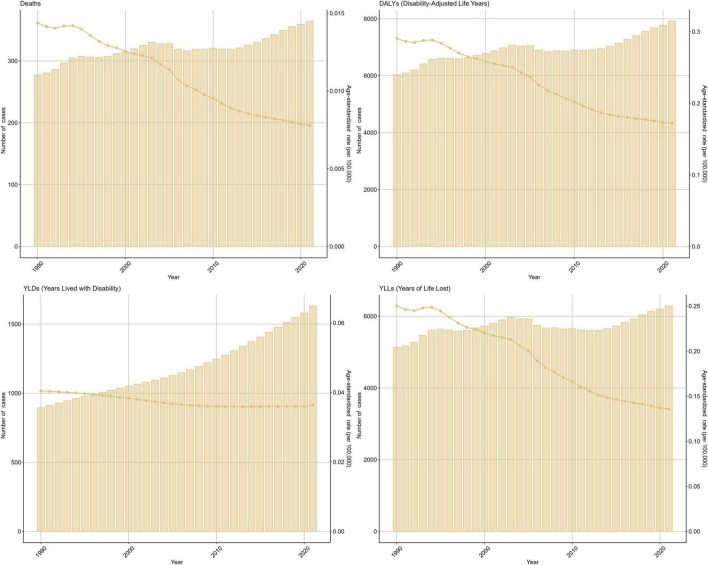
Global time trends (1990–2021) in ischemic stroke attributable to insufficient polyunsaturated fatty acid (PUFA) intake among women aged ≥ 50 years. Deaths. DALYs, disability-adjusted life years. YLDs, years lived with disability. YLLs, years of life lost. Units: rates per 100,000 population; DALYs/YLLs/YLDs (absolute) in years; contributions, % of total change; forecasts with 80 and 95% prediction intervals.

From 1990 to 2021, global age-standardized rates for ischemic stroke attributable to suboptimal omega-6 PUFA intake in women aged ≥ 50 years declined overall: DALYs [EAPC −1.32% (95% CI −1.52 to −1.12)], deaths [−1.47% (−1.71 to −1.23)], and YLLs [−1.61% (−1.82 to −1.40)], while YLD rates were approximately stable [+0.07% (−0.07 to +0.22)]. In 2021, the corresponding age-standardized rates were ASDR 0.17, ASYLDR 0.04, and ASYLLR 0.14 per 100,000 (Figure 1). Absolute counts increased over the same period, with the largest rise for YLDs; this pattern is explained by population growth and aging according to the decomposition (Supplementary Tables 1–4). To contextualize the burden in women ≥ 50 years relative to other groups, we conducted descriptive comparisons using GBD 2021 data. These analyses confirmed that the burden is predominantly concentrated in older adults: women ≥ 50 years had a substantially higher burden than younger women across all measures. Furthermore, among adults ≥ 50 years, men generally exhibited higher crude rates for deaths and DALYs, while YLD rates were similar between sexes (Supplementary Figure 1 and Supplementary Table 5).

### IS attributed to insufficient intake of PUFAs in different subgroups (age, SDI, region, and country) in terms of disease burden and trend of change age-stratified patterns

**FIGURE 2:**
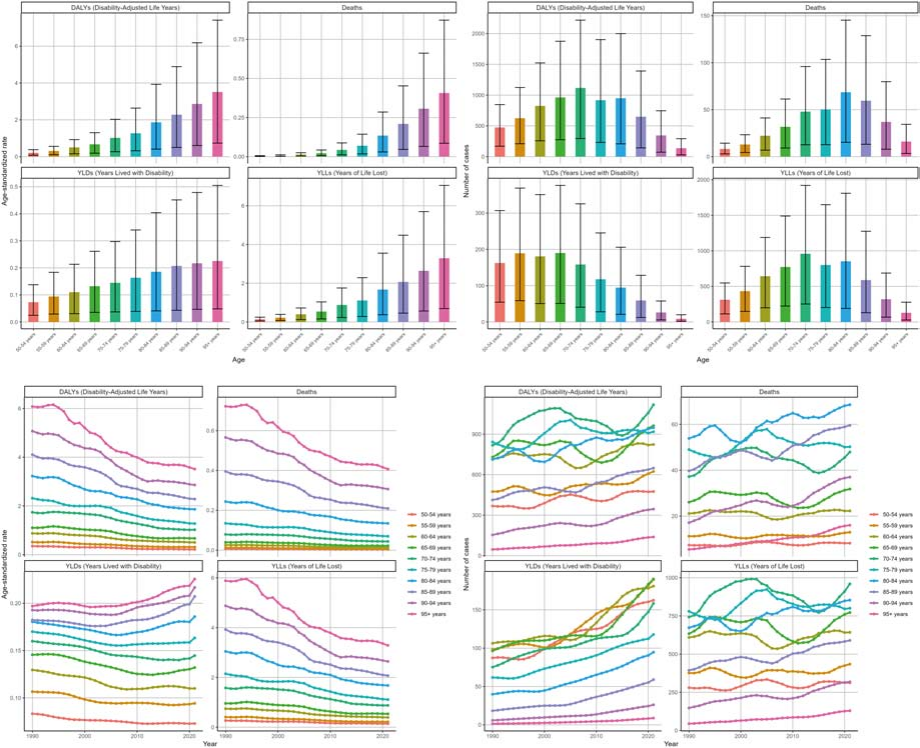
Age-specific burden in 2021 and trends (1990–2021) by 5-year age bands (women ≥ 50 years). Units: rates per 100,000 population; DALYs/YLLs/YLDs (absolute) in years; contributions, % of total change; forecasts with 80 and 95% prediction intervals.

From 1990 to 2021, age-standardized rates (ASRs, per 100,000) increased with age, whereas absolute counts peaked in the late-older strata. In 2021, the burden concentrated at ages 70–75 for DALYs and YLLs, deaths peaked later (around 80–84), and YLDs were spread across 50–74. For each 5-year age band, we pair the ASR with the absolute count and its 95% uncertainty interval (Figure 2; Supplementary Tables 1–4). Across age bands, ASRs generally declined over 1990–2021 (age-band–specific EAPCs are reported in Supplementary Tables 1–4), while absolute numbers rose, reflecting population growth and aging.

#### SDI gradients

**FIGURE 3:**
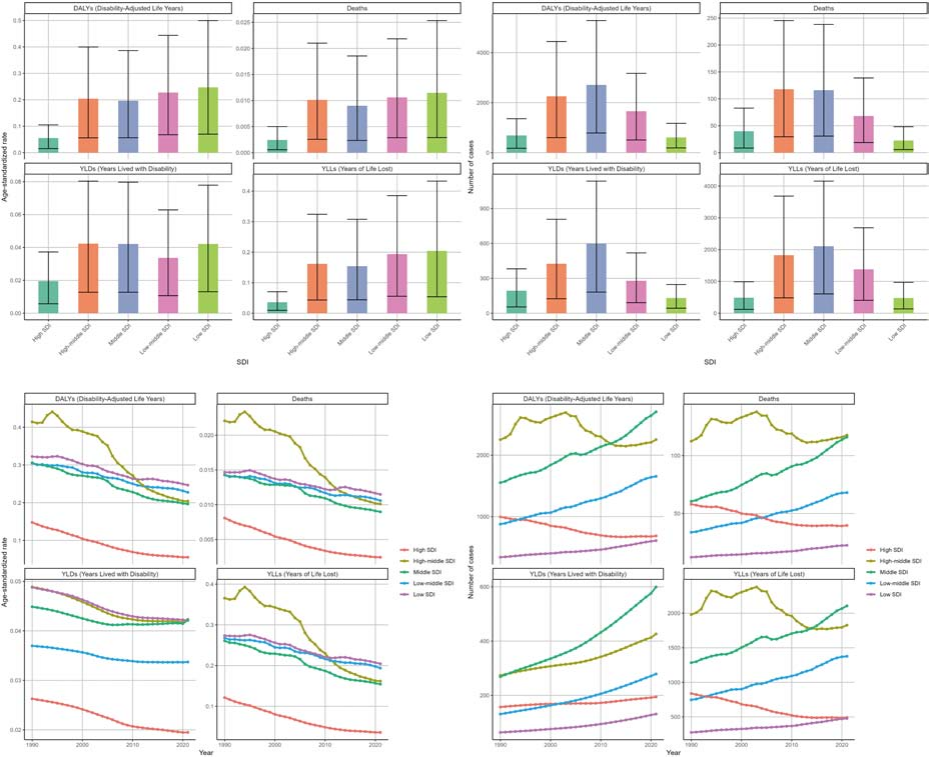
The distribution of the disease burden of ischemic stroke related to insufficient polyunsaturated fatty acid intake among women aged 50 and above worldwide in 2021 across different SDI regions and its trend changes from 1990 to 2021. SDI, sociodemographic index. Units: rates per 100,000 population; DALYs/YLLs/YLDs (absolute) in years; contributions, % of total change; forecasts with 80 and 95% prediction intervals.

By SDI, high-middle SDI locations experienced the fastest declines in ASRs over 1990–2021, whereas high-SDI locations consistently maintained the lowest ASRs. Despite a lower overall absolute burden, low-SDI settings showed the highest recent ASRs. For each SDI quintile, Figure 3 and Supplementary Tables 1–4 report ASR (per 100,000) together with the absolute count and 95% UI for deaths, DALYs, YLDs, and YLLs in 1990 and 2021, ensuring paired presentation of rates and counts.

#### Regional patterns

**FIGURE 4:**
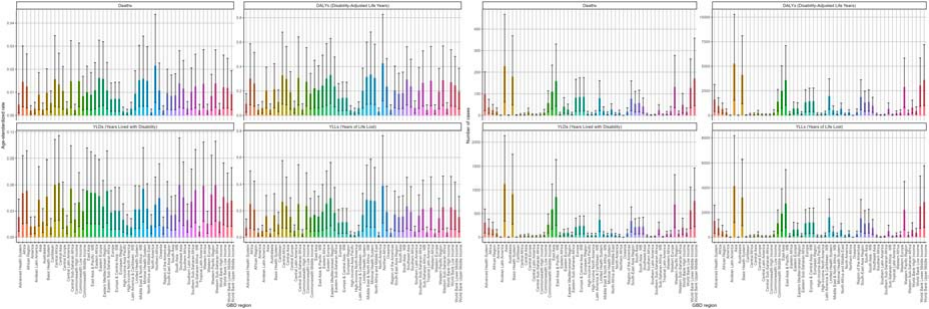
The distribution of the disease burden of ischemic stroke related to insufficient polyunsaturated fatty acid intake among women aged 50 and above worldwide in 2021 across different regions. Deaths. DALYs, disability-adjusted life years. YLDs, years lived with disability. YLLs, years of life lost. Units: rates per 100,000 population; DALYs/YLLs/YLDs (absolute) in years; contributions, % of total change; forecasts with 80 and 95% prediction intervals.

Across 54 GBD regions, larger-population groupings carried the largest absolute burden; Asia alone contributed > 60% of global DALYs and deaths. In 2021, several African/Middle Eastern groupings had higher rates, whereas most regions showed declining ASRs from 1990 to 2021; an exception was a rise in premature-mortality rates in Southern Sub-Saharan Africa (Figure 4; Supplementary Tables 1–4).

**FIGURE 5:**
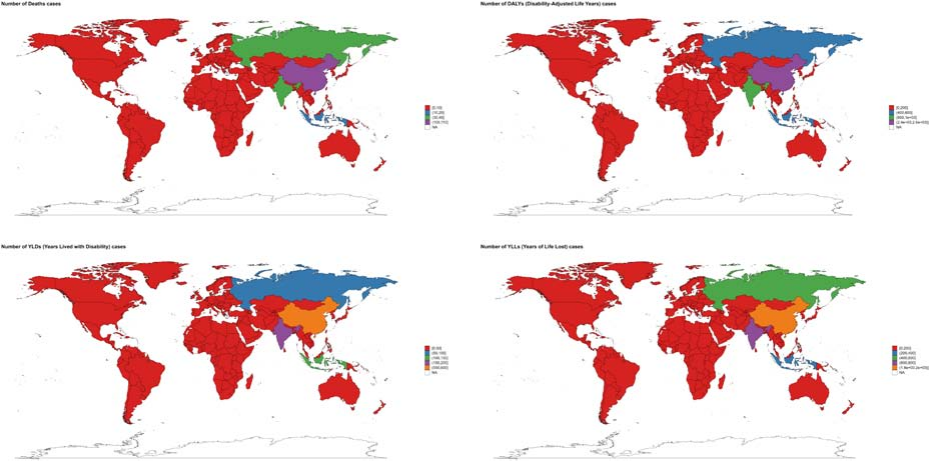
The geographical distribution of the disease burden of ischemic stroke related to insufficient intake of polyunsaturated fatty acids among women over 50 years old in 204 countries worldwide in 2021.

**FIGURE 6:**
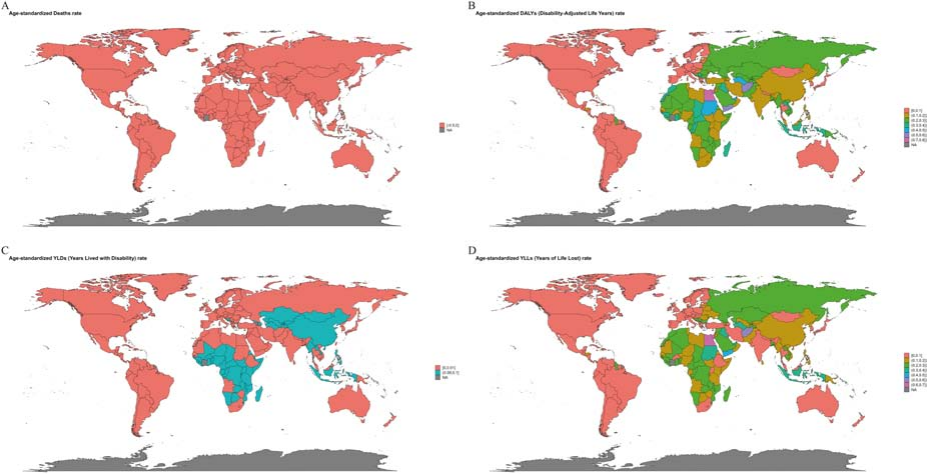
The geographic distribution of the age-standardized rate of ischemic stroke disease burden related to inadequate intake of polyunsaturated fatty acids among women over 50 years old in 204 countries worldwide in 2021. **(A)** Deaths. **(B)** DALYs, disability-adjusted life years. **(C)** YLDs, years lived with disability. **(D)** YLLs, years of life lost. Units: rates per 100,000 population; DALYs/YLLs/YLDs (absolute) in years; contributions, % of total change; forecasts with 80 and 95% prediction intervals.

**FIGURE 7:**
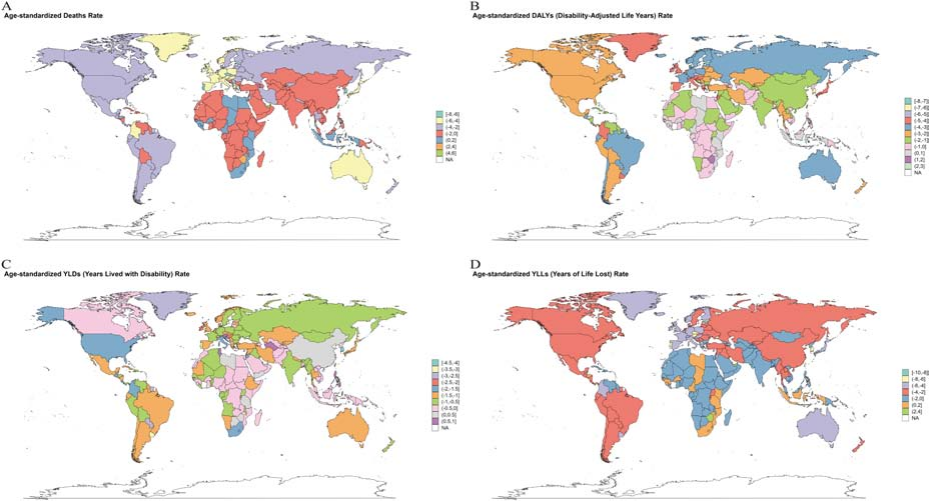
The geographic distribution of the EAPC of ischemic stroke disease burden related to insufficient intake of polyunsaturated fatty acids among women aged 50 and above globally from 1990 to 2021 across 204 countries. EAPC, Estimated Annual Percentage Change. **(A)** Deaths. **(B)** DALYs, disability-adjusted life years. **(C)** YLDs, years lived with disability. **(D)** YLLs, years of life lost. Units: rates per 100,000 population; DALYs/YLLs/YLDs (absolute) in years; contributions, % of total change; forecasts with 80 and 95% prediction intervals.

Among the 204 countries analyzed in 2021, China and India bore the largest absolute burden, jointly accounting for ∼43–45% of global DALYs, deaths, YLDs, and YLLs; China exceeded India across these outcomes (approximately 2–4 × ). Countries with the highest age-standardized rates clustered predominantly in North Africa, the Middle East, and parts of Central Asia, and the fastest increases were observed in selected countries in Central Asia, Southern Africa, and the Balkans (Figures 4–7; Supplementary Tables 1–4).

### Decomposition analysis

**FIGURE 8:**
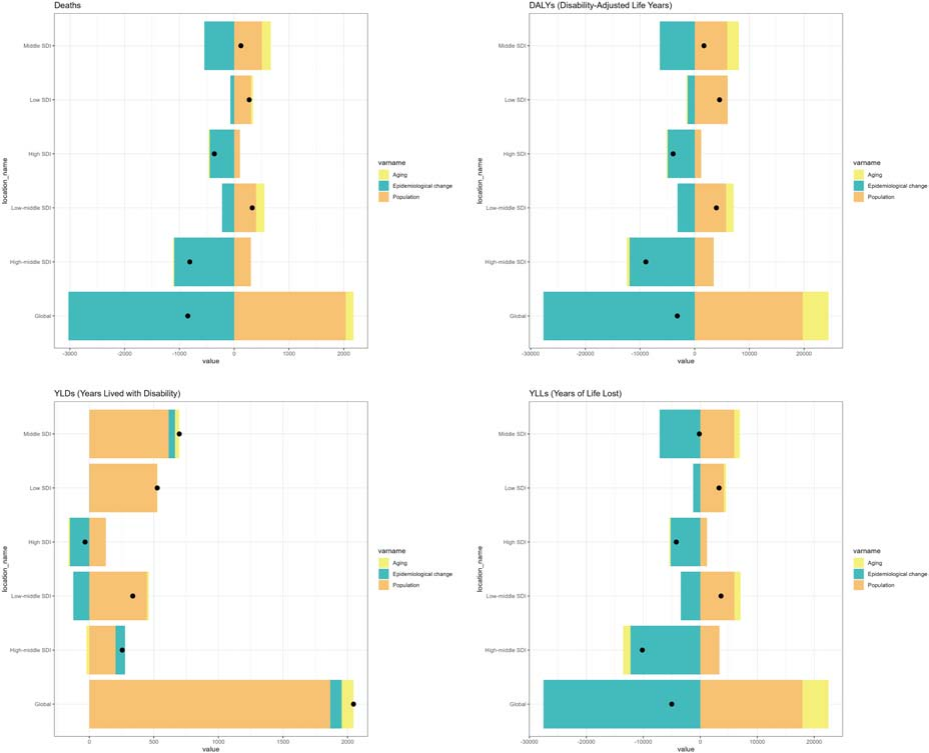
Decomposition of the 1990–2021 change in deaths/DALYs attributable to diet low in omega-6 PUFA. Stacked bars show percent contributions from population growth, population aging, and epidemiological change (age-specific rate change); components sum to 100% for each stratum. Error bars indicate 95% uncertainty intervals. SDI, Sociodemographic Index. Component-wise numerics are provided in Supplementary Tables 1–4; sex/age contrasts are summarized in Supplementary Table 5.

As illustrated in Figure 8, the GBD attributable to insufficient PUFAs intake has notably increased over the past three decades. The primary factors influencing the increase are population growth and epidemiological changes. Population growth has consistently contributed positively to each disease burden indicator across all SDI regions, with a more pronounced effect observed in Middle, Low-middle, and Low SDI regions. Conversely, epidemiological changes have generally contributed negatively to the disease burden, although they have had a positive effect on YLDs in High-middle and Middle SDI regions. In High and High-middle SDI settings, the negative contribution from epidemiological change—that is, declines in age-specific rates—was larger, offsetting demographic increases and yielding more pronounced declines in age-standardized rates. By contrast, population growth and population aging contributed positively but were smaller in magnitude than the epidemiological decline over most of the period. Globally, decomposition shows that the rise in absolute deaths, DALYs, YLDs and YLLs is driven primarily by population growth, with a smaller positive contribution from population aging. In contrast, epidemiological change (declines in age-specific rates) offsets part of these increases. The same pattern is observed across SDI strata; full percentage contributions (global–female and YLDs by SDI, Supplementary Table 6). The decomposition analysis showed that population growth and aging contributed positively to the increase in absolute counts of deaths, DALYs, YLDs, and YLLs, while epidemiologic change generally reduced ASRs over 1990–2021. Across strata, epidemiological change contributed negatively (i.e., reduced burden), whereas population growth and—though smaller—population aging contributed positively to increases in absolute numbers (details in Supplementary Tables 1–4).

### 2050 projections

**FIGURE 9:**
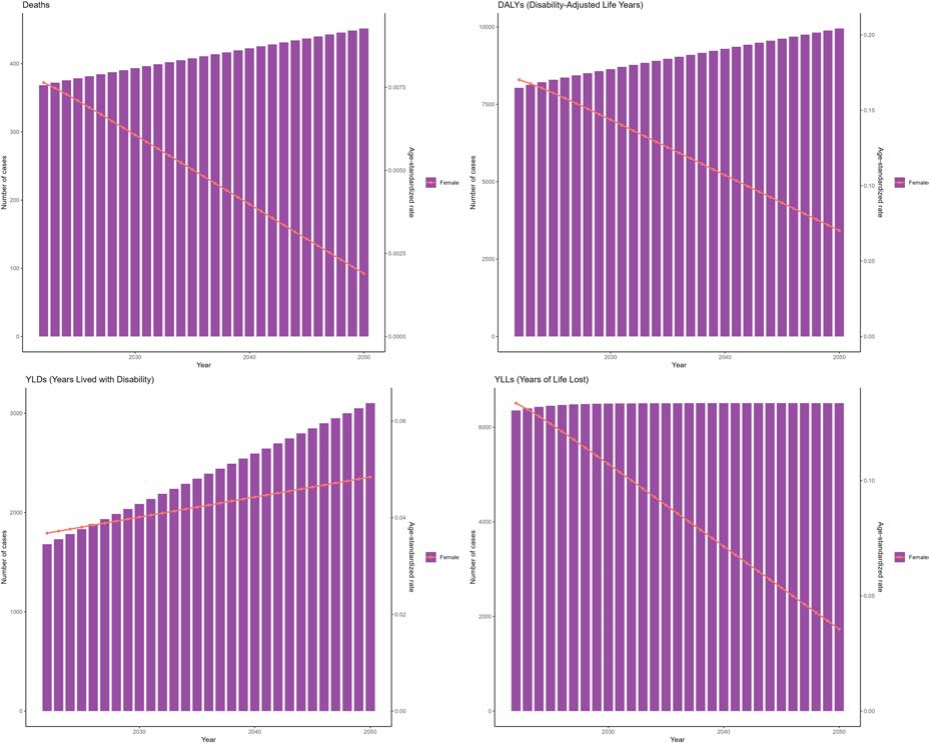
The ARIMA model predicts the trend of insufficient intake of polyunsaturated fatty acids and its associated ischemic stroke disease burden among women aged 50 and above globally until 2050. ARIMA, autoregressive integrated moving average. Deaths. DALYs, disability-adjusted life years. YLDs, years lived with disability. YLLs, years of life lost. Units: rates per 100,000 population; DALYs/YLLs/YLDs (absolute) in years; contributions, % of total change; forecasts with 80 and 95% prediction intervals.

**FIGURE 10:**
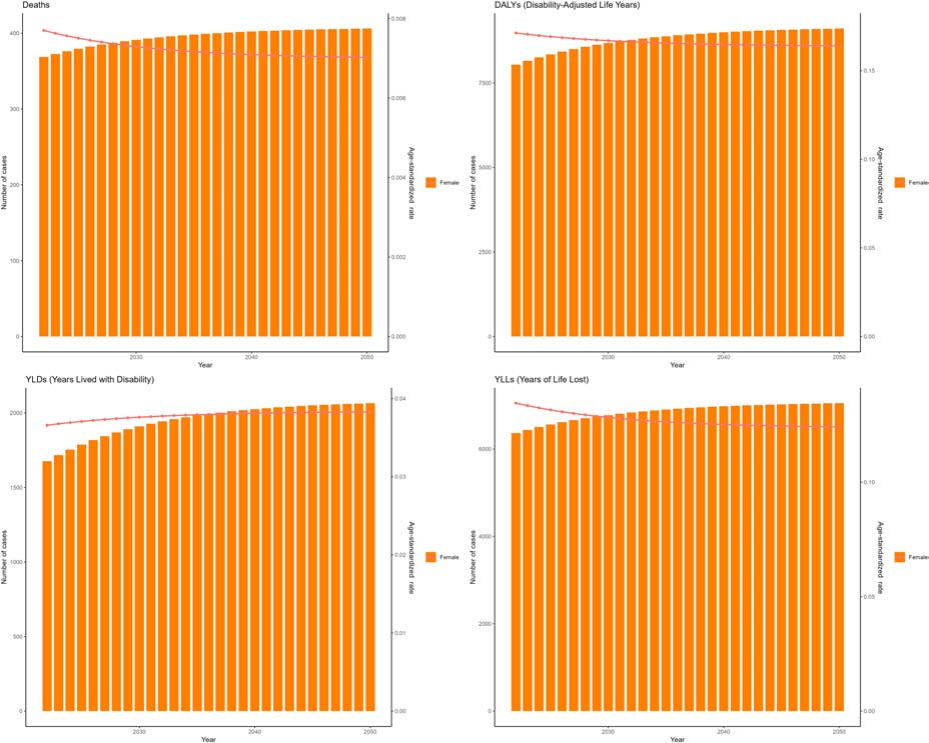
The EC model predicts the trend of insufficient intake of polyunsaturated fatty acids and its associated ischemic stroke disease burden among women aged 50 and above globally until 2050. EC, error-correction. Deaths. DALYs, disability-adjusted life years. YLDs, years lived with disability. YLLs, years of life lost. Units: rates per 100,000 population; DALYs/YLLs/YLDs (absolute) in years; contributions, % of total change; forecasts with 80 and 95% prediction intervals.

Selected models met residual diagnostics and showed low hold-out error (RMSE/MAPE); EC trajectories were consistent with ARIMA within the 80% and 95% prediction intervals. Results from both models predict a continued rise in the disease burden associated with IS due to insufficient intake of PUFAs in the coming decades, especially in terms of YLDs. Quantitatively, our models project that by 2050, global YLDs among women ≥ 50 years will increase by 68% versus 2021, whereas the age-standardized DALY rate is projected to decline by 18%, reinforcing the divergence of rising absolute burden alongside falling age-standardized rates. Projections to 2050 show a reduction in ASRs for mortality, DALYs, and YLLs (Figures 9, 10).

### External validation

To further validate the association between PUFAs and IS, we performed a country-level external validation for women aged ≥ 50 years, pairing Global Dietary Database (GDD, 2018)^1^ intakes, total ω-6 (% energy) and seafood ω-3 (mg/day), with GBD 2021 country-level IS DALY rates attributable to “diet low in PUFA” (year 2018). Data definitions, units, and risk–outcome specifications followed the public documentation of GDD and the GBD results tool.

Results indicated that ω-6 PUFA intake was inversely related to the attributable burden (Pearson *r* = −0.211, *p* < 0.01; Spearman ρ = −0.292, *p* < 0.001). In a log-linear model of ln(DALY rate) versus exposure, β = −1.200 (*p* < 0.01, *R*^2^ = 0.055), implying ≈70% lower DALY rate per +1%E ω-6 (95% CI −85.6 to −36.9%). Seafood ω-3 showed the same direction with smaller magnitude (*r* = −0.172, *p* < 0.05, *R*^2^ = 0.029). These ecological findings are directionally consistent with the GBD risk specification for diet low in PUFA.

## Discussion

This investigation represents the inaugural comprehensive analysis examining the worldwide health impact of insufficient PUFAs intake among women aged ≥ 50 years, and the first to explicitly center older women as the focal population at a global scale, spanning 204 countries and territories within the GBD 2021 comparative risk framework. Our findings indicate that while ASRs for PUFA-associated IS have consistently declined over time, the absolute numbers continue to rise, with the most pronounced increase observed in YLDs. Moreover, this investigation reveals substantial disparities in health burden patterns among various age cohorts, geographical territories, and national populations, with each demographic category being shaped by unique contributing elements. Higher age groups exhibited higher ASRs, while younger populations were primarily affected by YLDs. Notably, Low, Low-middle, and Middle SDI regions have experienced a continuous increase in disease burden, whereas High and High-middle SDI regions have seen an overall decline. We interpret regional and demographic contrasts as ecological signals of population structure and exposure distributions, rather than individual-level causation. These regional differences appear to be largely driven by population growth in lower SDI regions and epidemiological changes in higher SDI regions. Between-location differences reflect GBD 2021 population-attributable burden for omega-6 PUFA intake below the TMREL and should not be interpreted as whole-diet causation.

Regional patterns likely reflect both diet quality/PUFA sources and health-system capacity. In high-income coastal settings, higher consumption of marine omega-3 and liquid vegetable oils, together with better detection and control of hypertension and stronger stroke services, aligns with declining age-standardized burden (9, 27). In several low-income coastal settings, however, access does not equal intake: fish may be unaffordable or export-oriented and cold-chain/market access limited (28), while household cooking relies on palm-rich solid fats amid the nutrition transition (29); together with low hypertension control, these factors help explain rising burden despite coastal location (27).

The disease burden of PUFA-associated IS increased with age, which aligns with previous studies that have explored the impact of all-risk factors on a population-wide scale (30). Age has long been recognized as a key risk factor for IS, as it is associated with vascular changes, such as thickening and hardening of blood vessels, which increase the risk of stroke. Additionally, older individuals are more likely to have comorbidities like hypertension, diabetes mellitus, and dyslipidemia, all of which are well-established risk factors for IS.

Our study also found that the ASRs for disease burden were generally lower, and the EAPC showed the fastest decline in coastal high-income regions and countries, including Estonia, the Republic of Korea, Israel, Taiwan, Puerto Rico, Norway, Belgium, Switzerland, Malta, France, the United States, and the United States Virgin Islands. However, several low-income coastal countries, such as Yemen, Indonesia, Turkmenistan, the United Arab Emirates, Nauru, the Solomon Islands, Vanuatu, Madagascar, Fiji, the Philippines, Tunisia, and Lebanon, exhibited higher ASRs for the disease burden, and their EAPCs were also increasing rapidly.

Despite the fact that PUFA-rich foods such as fish, shrimp, and crab are central to the diets of many of the countries mentioned above, the differing trends in disease burden suggest that supplementation with PUFAs alone is not sufficient to completely prevent IS-related disease burden. This indicates that more comprehensive strategies, including access to adequate economic and medical resources, are needed to address the issue. This conclusion is further supported by our analysis across SDI regions, which showed a general decline in disease burden in higher SDI regions, while the burden increased in medium and low SDI regions. Additionally, we compared China and India, two countries with similar population sizes, and found that the disease burden in China was significantly higher than in India. This discrepancy may be consistent with differences in sodium intake (31), hypertension control (27), and population aging (32); these are population-level hypotheses and should not be interpreted as causal without dedicated comparative analyses.

Our decomposition analysis revealed that epidemiological changes generally had a negative impact on the disease burden of PUFA-associated IS both globally and across SDI regions. The effect was particularly pronounced in the High and High-middle SDI regions, as well as in the Middle SDI regions, where the contribution from epidemiological changes was substantial. In contrast, Low and Low-middle SDI regions were primarily affected by population growth, which drove the increase in disease burden. This disparity highlights a global imbalance in the burden of IS attributed to PUFAs, underscoring the need for more targeted policy interventions and healthcare resource investments in higher SDI regions.

Additionally, our analysis indicates that aging has not disproportionately affected the disease burden in either Low or High SDI regions. In Low SDI regions, the impact of aging may not be fully realized, possibly due to shorter life expectancies and limited medical interventions. Conversely, in High SDI regions, the burden of IS on older populations appears less pronounced, likely due to better health awareness, preventive measures, and timely treatments. The burden of PUFA-associated IS was predominantly associated with the loss of life expectancy, with a nearly 4:1 ratio compared to the loss of disability. However, younger individuals were more affected by the loss of disability, suggesting a greater need for long-term rehabilitation and therapeutic care. This is especially concerning as it may lead to long-term family and social challenges, highlighting the importance of addressing these issues in future healthcare planning and policy.

For individuals at high risk of IS, we recommend appropriate supplementation with foods rich in PUFAs, such as seafood (e.g., deep-sea fish, shrimp, and crab) and certain nuts and vegetable oils. These dietary choices have been shown to effectively reduce the disease burden of IS, as demonstrated in two recent large-scale studies (33, 34). Estruch et al. found that a Mediterranean diet, which includes olive oil or nuts, significantly lowered cardiovascular event rates compared to a low-fat diet, based on a Spanish population of individuals aged 55 years and older, with 57% of participants being women (34). Another large-scale study, based on three databases, reported a negative association between PUFA intake and stroke risk (33).

While the exact recommended intake of PUFAs remains uncertain, it is suggested that they should constitute 5–10% of total energy intake (35). Given the complex interplay of multiple risk factors, it is essential to monitor and manage blood pressure, blood glucose, and lipid levels, in addition to adjusting lifestyle habits. Furthermore, as previously discussed, a country’s economic level, national health awareness, and sanitary conditions can significantly influence the disease burden of PUFA-associated IS.

We advocate for providing support and assistance to Low SDI regions and countries, particularly in terms of technical, knowledge, and medical resources. It is well established that early access to thrombolytic therapy, such as alteplase, within the appropriate time window greatly improves the prognosis of stroke patients and significantly reduces DALYs. Therefore, we recommend that regions with a high burden of IS and the necessary infrastructure should follow the example of China in establishing national stroke centers (36, 37).

Previous studies have also assessed the global disease burden of IS associated with various risk factors, including diet, lead exposure, and smoking (38–40). However, it is notable that recent research has increasingly focused on the impact of PUFAs on the disease burden of IS. A large-scale study reported that total n-3 PUFA intake, including DHA, and n-6 PUFA intake, including linoleic acid (LA), were negatively correlated with stroke risk (33). Visioli et al. hypothesized that the underlying mechanism involves the activation of oxidative enzymes and proliferator-activated receptors (PPARs), which regulate oxylipins involved in glucose and lipid metabolism (35). In the GBD framework, our exposure is “diet low in polyunsaturated fat,” representing total PUFA intake—predominantly non-seafood sources (n-6 linoleic acid and plant ALA)—whereas seafood-derived long-chain n-3 (EPA/DHA) is modeled as a separate risk factor. This distinction matters: increasing n-6-rich plant oils in place of saturated fat improves lipids and is linked to lower cardiovascular risk, while marine n-3 confers anti-inflammatory and antithrombotic effects relevant to stroke prevention (9, 41). Accordingly, our attributable estimates capture the burden tied to shortfalls in total (mainly n-6/ALA) PUFA, not the seafood n-3 component. Policy should therefore encompass both wider access to PUFA-rich vegetable oils, nuts, and seeds and promotion of regular oily-fish intake.

For public health implications, the substantial global burden of PUFA-deficient ischemic stroke among women over 50 demands the immediate integration of targeted nutritional interventions into national stroke prevention programs. Healthcare systems must establish evidence-based dietary guidelines recommending 5–10% of total energy intake from polyunsaturated fatty acids, with particular emphasis on accessible sources such as fish, nuts, and plant oils for postmenopausal women. The disproportionate burden in low-middle SDI regions necessitates culturally adapted, cost-effective dietary education campaigns that address local food availability and economic constraints. Given that years lived with disability constitute the primary burden component, prevention strategies should prioritize early intervention over post-stroke rehabilitation (42), emphasizing the critical importance of nutritional counseling in primary healthcare settings for this high-risk population.

Prevention should pair system-level programs with clinician-level advice. Health systems ought to integrate targeted nutrition into stroke prevention and recommend 5–10% of energy from PUFA, emphasizing accessible sources for postmenopausal women (fish, nuts, plant oils). For adults ≥ 50, clinicians should replace solid animal/tropical fats and partially hydrogenated fats with liquid PUFA-rich oils, encourage oily fish twice weekly (or ALA-rich plants where seafood is scarce), and add nuts/seeds several times per week, alongside routine control of blood pressure, lipids, diabetes, and smoking (12, 41). In low- and middle-SDI settings, feasible levers include public procurement defaults to liquid oils, reformulation and front-of-pack labeling, and measures that improve supply and affordability of PUFA-rich oils and fish/ALA sources, with brief dietary counseling embedded in primary care (9, 12, 41). The core replacement message is universal; tailoring is context-specific, with high-SDI settings emphasizing procurement/labeling, middle-SDI using price supports plus community health-worker counseling, and low-SDI prioritizing basic availability/affordability; this focus is warranted as YLDs constitute a large share of the burden.

This study has limitations. First, because our estimates derive from the GBD comparative risk assessment and rely on observational risk–outcome associations, they should not be interpreted as individual-level causal effects. The GBD framework quantifies attributable fractions and burdens rather than defining a distinct disease entity. Dietary exposures partly rely on modeled or imputed inputs and heterogeneous instruments across countries; some misclassification between total PUFA (largely n-6) and seafood omega-3 may persist within the GBD risk-factor structure. The GBD database has inherent shortcomings, including diversity and variable quality of sources, challenges in diagnosing ischemic stroke, particularly in low-SDI settings with limited MRI access, and potential model error. Our forward projections are author-derived status-quo scenarios based on GBD 2021 estimates and incorporate statistical uncertainty; unmodeled policy or care improvements could shift future trajectories. Finally, our regional interpretation is contextual rather than causal; ecological, risk-specific attribution cannot fully separate the joint influences of overall diet patterns, PUFA sources and supply chains, blood-pressure control, and health-system capacity.

### Future research directions

As the study focused solely on the burden of PUFA-attributed IS in a specific population, a comprehensive assessment covering the entire population is essential. Moreover, significant disparities in the burden and temporal trends of PUFA-related IS were observed across countries, particularly in coastal regions. These variations warrant further investigation through epidemiological studies and correlation analyses examining differences in dietary patterns, lifestyle factors, and cultural practices. Lastly, future research should aim to elucidate the optimal intake levels and supplementation strategies for PUFAs, explore the biological mechanisms underlying their influence on IS onset and progression, and define the dose–response relationship between PUFA consumption and IS risk.

## Conclusion

In conclusion, our study demonstrates a persistent escalation in the global burden of IS linked to insufficient intake of PUFAs, with substantial variation across age groups, SDIs, regions, and countries. The findings highlight the complexity and variability of the burden of PUFA-associated IS. Through this study, we aim to bring more attention to this specific group of PUFA-associated IS patients and emphasize the need for global high-risk populations to adjust poor dietary habits. Daily supplementation with PUFAs may be beneficial for reducing stroke risk in older women. However, some of the conclusions drawn in this study remain speculative due to a lack of more definitive evidence, and caution is needed when interpreting these results. Future research should focus on establishing clearer causal relationships between PUFA intake and stroke outcomes, as well as developing targeted interventions that address region-specific challenges in stroke prevention and management.

## Data Availability

The original contributions presented in this study are included in the article/Supplementary material, further inquiries can be directed to the corresponding author.

## Footnotes

1
https://www.globaldietarydatabase.org/
