# Supplementary material for: Global burden of disease analysis and projections of ischemic stroke linked to inadequate polyunsaturated fatty acid intake in older women (1990–2021)
Source: Front Nutr. 2025 Dec 12;12:1659895. doi: 10.3389/fnut.2025.1659895 (PMC12742205; doi:10.3389/fnut.2025.1659895)
Supplement: Supplementary file 1 [file Table_1.docx]

Table 1 The DALYs cases and age-standardized DALYs rate of ischemic stroke attributed to IIPUFAs disease in 1990 and 2021, along with their temporal trend.

|  | Rate per 100 000(95%UI) | | 2021 | | 1990-2021 |
| --- | --- | --- | --- | --- | --- |
|  | 1990 | |  | |  |
|  | DALYs cases | The age-standardized DALYs rate | DALYs cases | The age-standardized DALYs rate | EAPC |

| **Global** | 6026 (1794-11424) | 0.29 (0.09-0.55) | 7917 (2314-15162) | 0.17 (0.05-0.33) | -1.32 (-1.52--1.12) |
| --- | --- | --- | --- | --- | --- |
| **Age** |  |  |  |  |  |
| 50-54 years | 368 (132-654) | 0.35 (0.13-0.62) | 475 (169-846) | 0.21 (0.08-0.38) | -1.74 (-1.86--1.63) |
| 55-59 years | 475 (158-861) | 0.51 (0.17-0.93) | 624 (210-1124) | 0.31 (0.1-0.56) | -1.97 (-2.09--1.85) |
| 60-64 years | 716 (217-1328) | 0.87 (0.26-1.62) | 824 (258-1523) | 0.5 (0.16-0.93) | -2.11 (-2.25--1.97) |
| 65-69 years | 730 (207-1406) | 1.1 (0.31-2.12) | 962 (275-1875) | 0.67 (0.19-1.3) | -2.17 (-2.38--1.96) |
| 70-74 years | 818 (213-1645) | 1.74 (0.45-3.5) | 1118 (294-2218) | 1.02 (0.27-2.03) | -2.12 (-2.32--1.91) |
| 75-79 years | 842 (213-1730) | 2.32 (0.59-4.76) | 918 (231-1902) | 1.27 (0.32-2.64) | -2.02 (-2.16--1.89) |
| 80-84 years | 713 (163-1504) | 3.23 (0.74-6.81) | 948 (208-2000) | 1.86 (0.41-3.93) | -1.97 (-2.04--1.89) |
| 85-89 years | 413 (91-879) | 4.11 (0.9-8.75) | 649 (143-1392) | 2.28 (0.5-4.89) | -2.09 (-2.21--1.97) |
| 90-94 years | 153 (33-326) | 5.07 (1.08-10.76) | 345 (73-746) | 2.86 (0.6-6.18) | -2.16 (-2.32--2) |
| 95+ years | 46 (10-97) | 6.08 (1.29-12.83) | 138 (29-292) | 3.51 (0.74-7.41) | -2.07 (-2.22--1.92) |
| **SDI region** |  |  |  |  |  |
| High SDI | 996 (262-1942) | 0.15 (0.04-0.28) | 686 (176-1357) | 0.06 (0.02-0.1) | -3.42 (-3.56--3.28) |
| High-middle SDI | 2253 (645-4323) | 0.41 (0.12-0.8) | 2254 (602-4443) | 0.2 (0.06-0.4) | -2.81 (-3.06--2.56) |
| Middle SDI | 1553 (477-3004) | 0.31 (0.09-0.6) | 2706 (787-5281) | 0.2 (0.06-0.39) | -1.55 (-1.62--1.47) |
| Low-middle SDI | 878 (287-1657) | 0.3 (0.1-0.58) | 1655 (508-3170) | 0.23 (0.07-0.44) | -1 (-1.05--0.95) |
| Low SDI | 338 (107-659) | 0.32 (0.1-0.65) | 609 (190-1172) | 0.25 (0.07-0.5) | -0.97 (-1.04--0.91) |
| **GBD region** |  |  |  |  |  |
| Advanced Health System | 2439 (653-4689) | 0.24 (0.07-0.46) | 1618 (408-3144) | 0.09 (0.03-0.18) | -3 (-3.79--2.2) |
| Africa | 524 (167-965) | 0.39 (0.12-0.74) | 961 (297-1792) | 0.3 (0.09-0.59) | -0.71 (-1.83-0.43) |
| African Region | 340 (108-665) | 0.32 (0.1-0.64) | 651 (197-1252) | 0.26 (0.07-0.52) | -0.69 (-1.89-0.52) |
| America | 318 (87-600) | 0.09 (0.03-0.17) | 341 (94-653) | 0.05 (0.01-0.09) | -1.85 (-2.11--1.58) |
| Andean Latin America | 12 (4-23) | 0.12 (0.04-0.22) | 19 (5-35) | 0.06 (0.02-0.11) | -1.62 (-2.13--1.11) |
| Asia | 3066 (970-5790) | 0.31 (0.09-0.59) | 5267 (1549-10284) | 0.2 (0.06-0.4) | -0.58 (-0.92--0.23) |
| Australasia | 18 (5-37) | 0.14 (0.03-0.27) | 16 (4-32) | 0.05 (0.01-0.09) | -2.82 (-3.57--2.07) |
| Basic Health System | 2452 (747-4791) | 0.33 (0.1-0.66) | 4119 (1181-8108) | 0.21 (0.06-0.42) | -0.48 (-0.8--0.15) |
| Caribbean | 25 (7-48) | 0.19 (0.06-0.36) | 32 (9-61) | 0.11 (0.03-0.21) | -0.94 (-1.16--0.72) |
| Central Africa | 53 (16-100) | 0.39 (0.11-0.75) | 103 (31-205) | 0.33 (0.09-0.68) | -0.79 (-2.05-0.49) |
| Central Asia | 114 (34-212) | 0.41 (0.12-0.77) | 133 (39-248) | 0.3 (0.09-0.56) | -1.39 (-1.73--1.06) |
| Central Europe | 378 (104-726) | 0.45 (0.12-0.86) | 263 (70-511) | 0.19 (0.05-0.36) | -2.22 (-3--1.43) |
| Central Latin America | 57 (17-104) | 0.14 (0.04-0.26) | 83 (24-155) | 0.06 (0.02-0.12) | -1.56 (-2.12--0.98) |
| Central Sub-Saharan Africa | 39 (11-75) | 0.37 (0.1-0.73) | 81 (23-162) | 0.31 (0.08-0.64) | -0.78 (-2.06-0.52) |
| Commonwealth High Income | 141 (36-279) | 0.15 (0.04-0.29) | 79 (19-157) | 0.05 (0.01-0.09) | -3.38 (-4.25--2.51) |
| Commonwealth Low Income | 134 (41-255) | 0.35 (0.1-0.68) | 293 (84-577) | 0.28 (0.08-0.55) | -0.02 (-1.24-1.21) |
| Commonwealth Middle Income | 707 (227-1375) | 0.25 (0.08-0.49) | 1403 (434-2642) | 0.18 (0.05-0.34) | -0.53 (-1.23-0.17) |
| East Asia | 1518 (457-2999) | 0.36 (0.1-0.71) | 2487 (704-5049) | 0.22 (0.06-0.45) | -0.41 (-0.73--0.08) |
| East Asia & Pacific - WB | 2180 (665-4281) | 0.32 (0.09-0.64) | 3581 (1026-7103) | 0.21 (0.06-0.41) | -0.43 (-0.72--0.13) |
| Eastern Africa | 112 (36-215) | 0.34 (0.1-0.66) | 194 (59-381) | 0.24 (0.07-0.49) | -1.1 (-2.4-0.22) |
| Eastern Europe | 1082 (298-2068) | 0.6 (0.17-1.14) | 692 (178-1366) | 0.3 (0.08-0.59) | -2.62 (-3.41--1.82) |
| Eastern Mediterranean Region | 394 (121-724) | 0.48 (0.14-0.91) | 707 (225-1290) | 0.33 (0.1-0.63) | -0.89 (-1.82-0.04) |
| Eastern Sub-Saharan Africa | 109 (34-213) | 0.31 (0.09-0.62) | 197 (62-383) | 0.24 (0.07-0.48) | -0.91 (-2.28-0.47) |
| Europe | 2109 (569-4053) | 0.33 (0.09-0.62) | 1340 (342-2610) | 0.14 (0.04-0.26) | -2.83 (-3.67--1.99) |
| Europe & Central Asia - WB | 2190 (592-4198) | 0.33 (0.09-0.63) | 1434 (372-2767) | 0.14 (0.04-0.27) | -2.76 (-3.53--1.98) |
| European Region | 2198 (595-4212) | 0.33 (0.09-0.63) | 1445 (375-2786) | 0.14 (0.04-0.27) | -2.76 (-3.53--1.98) |
| High-income Asia Pacific | 184 (48-358) | 0.17 (0.04-0.32) | 133 (32-271) | 0.04 (0.01-0.08) | -3.21 (-4.27--2.14) |
| High-income North America | 129 (34-253) | 0.06 (0.02-0.12) | 115 (29-227) | 0.03 (0.01-0.06) | -2.24 (-2.98--1.49) |
| Latin America & Caribbean - WB | 190 (54-352) | 0.14 (0.04-0.27) | 227 (64-426) | 0.06 (0.02-0.11) | -1.85 (-2.23--1.48) |
| Limited Health System | 1014 (324-1905) | 0.28 (0.08-0.54) | 1966 (608-3728) | 0.2 (0.06-0.39) | -0.63 (-1.43-0.18) |
| Middle East & North Africa - WB | 292 (90-550) | 0.5 (0.14-0.96) | 515 (155-963) | 0.31 (0.09-0.6) | -0.86 (-1.65--0.06) |
| Minimal Health System | 113 (34-217) | 0.4 (0.11-0.8) | 205 (60-405) | 0.33 (0.09-0.68) | -0.85 (-2.11-0.44) |
| North Africa and Middle East | 403 (124-740) | 0.5 (0.14-0.94) | 672 (202-1257) | 0.31 (0.09-0.58) | -0.96 (-1.69--0.23) |
| North America | 129 (34-253) | 0.06 (0.02-0.12) | 115 (29-227) | 0.03 (0.01-0.06) | -2.24 (-2.98--1.49) |
| Northern Africa | 175 (53-346) | 0.61 (0.17-1.23) | 303 (89-568) | 0.43 (0.12-0.83) | -0.43 (-1.21-0.35) |
| Oceania | 4 (1-7) | 0.27 (0.08-0.55) | 8 (3-15) | 0.24 (0.07-0.47) | -0.29 (-1.38-0.81) |
| Region of the Americas | 318 (87-600) | 0.09 (0.03-0.17) | 341 (94-653) | 0.05 (0.01-0.09) | -1.85 (-2.11--1.58) |
| South-East Asia Region | 926 (306-1752) | 0.28 (0.09-0.54) | 1864 (578-3621) | 0.21 (0.06-0.4) | -0.25 (-0.84-0.35) |
| South Asia | 640 (207-1262) | 0.25 (0.08-0.5) | 1297 (400-2469) | 0.18 (0.05-0.34) | -0.4 (-1.09-0.29) |
| South Asia - WB | 680 (223-1327) | 0.25 (0.08-0.52) | 1369 (423-2598) | 0.18 (0.06-0.35) | -0.42 (-1.11-0.27) |
| Southeast Asia | 473 (150-882) | 0.37 (0.11-0.71) | 974 (293-1836) | 0.29 (0.08-0.56) | 0.17 (-0.35-0.69) |
| Southern Africa | 58 (18-110) | 0.25 (0.08-0.49) | 118 (36-222) | 0.24 (0.07-0.46) | -0.01 (-0.96-0.95) |
| Southern Latin America | 43 (12-82) | 0.17 (0.05-0.32) | 32 (8-62) | 0.06 (0.02-0.12) | -2.47 (-2.8--2.13) |
| Southern Sub-Saharan Africa | 33 (10-63) | 0.22 (0.06-0.42) | 63 (18-119) | 0.2 (0.06-0.39) | 0.4 (-0.26-1.06) |
| Sub-Saharan Africa - WB | 350 (112-692) | 0.33 (0.1-0.66) | 660 (197-1267) | 0.27 (0.08-0.53) | -0.74 (-1.96-0.49) |
| Tropical Latin America | 53 (15-101) | 0.12 (0.03-0.23) | 63 (17-123) | 0.04 (0.01-0.09) | -2.01 (-2.4--1.61) |
| Western Africa | 125 (38-264) | 0.34 (0.1-0.72) | 243 (73-461) | 0.28 (0.08-0.56) | -0.79 (-2.06-0.49) |
| Western Europe | 572 (142-1141) | 0.15 (0.04-0.29) | 283 (66-576) | 0.04 (0.01-0.09) | -3.42 (-4.55--2.29) |
| Western Pacific Region | 1836 (547-3620) | 0.32 (0.09-0.63) | 2896 (806-5837) | 0.19 (0.05-0.39) | -0.54 (-0.86--0.22) |
| Western Sub-Saharan Africa | 139 (43-290) | 0.34 (0.1-0.72) | 275 (83-520) | 0.29 (0.08-0.57) | -0.73 (-2-0.56) |
| World Bank High Income | 1255 (324-2447) | 0.16 (0.04-0.3) | 785 (196-1558) | 0.05 (0.01-0.1) | -3.05 (-3.92--2.16) |
| World Bank Low Income | 259 (82-484) | 0.36 (0.11-0.7) | 456 (135-869) | 0.27 (0.08-0.54) | -1.02 (-2.14-0.12) |
| World Bank Lower Middle Income | 1725 (547-3272) | 0.35 (0.11-0.68) | 3055 (936-5853) | 0.25 (0.07-0.48) | -0.71 (-1.4--0.02) |
| World Bank Upper Middle Income | 2778 (816-5370) | 0.37 (0.11-0.72) | 3613 (996-7202) | 0.19 (0.05-0.39) | -1.4 (-1.7--1.11) |
| **Countries** |  |  |  |  |  |
| Afghanistan | 23 (7-47) | 0.72 (0.19-1.47) | 35 (12-74) | 0.62 (0.18-1.33) | -1.52 (-2.62--0.4) |
| Albania | 2 (1-4) | 0.18 (0.05-0.36) | 3 (1-7) | 0.15 (0.04-0.3) | 1.43 (0.98-1.88) |
| Algeria | 24 (7-48) | 0.49 (0.13-1.02) | 44 (12-87) | 0.3 (0.08-0.61) | -0.66 (-1.53-0.21) |
| American Samoa | 0 (0-0) | 0.21 (0.06-0.4) | 0 (0-0) | 0.19 (0.05-0.37) | 0.74 (0-1.49) |
| Andorra | 0 (0-0) | 0.07 (0.02-0.15) | 0 (0-0) | 0.04 (0.01-0.08) | -0.79 (-1.47--0.1) |
| Angola | 5 (1-9) | 0.27 (0.08-0.53) | 14 (4-26) | 0.24 (0.06-0.46) | -0.71 (-2.2-0.79) |
| Antigua and Barbuda | 0 (0-0) | 0.09 (0.02-0.17) | 0 (0-0) | 0.07 (0.02-0.14) | -0.66 (-0.87--0.45) |
| Argentina | 28 (8-54) | 0.16 (0.04-0.31) | 20 (5-39) | 0.06 (0.02-0.12) | -2.48 (-2.82--2.14) |
| Armenia | 6 (2-11) | 0.38 (0.11-0.72) | 5 (1-10) | 0.21 (0.06-0.39) | -1.79 (-2.21--1.38) |
| Australia | 16 (4-31) | 0.14 (0.03-0.27) | 13 (3-26) | 0.05 (0.01-0.09) | -2.92 (-3.69--2.15) |
| Austria | 15 (4-30) | 0.18 (0.05-0.36) | 6 (1-12) | 0.05 (0.01-0.1) | -3.88 (-4.99--2.75) |
| Azerbaijan | 7 (2-13) | 0.24 (0.07-0.46) | 10 (3-21) | 0.2 (0.06-0.41) | -0.41 (-0.72--0.11) |
| Bahamas | 0 (0-0) | 0.09 (0.02-0.17) | 0 (0-0) | 0.07 (0.02-0.15) | 0.35 (0.01-0.69) |
| Bahrain | 0 (0-0) | 0.31 (0.09-0.61) | 0 (0-1) | 0.16 (0.05-0.32) | -2.15 (-3.47--0.81) |
| Bangladesh | 76 (23-146) | 0.4 (0.11-0.79) | 179 (52-354) | 0.29 (0.08-0.6) | 0.49 (-0.68-1.66) |
| Barbados | 0 (0-1) | 0.18 (0.05-0.36) | 0 (0-1) | 0.12 (0.03-0.24) | -0.81 (-1.44--0.17) |
| Belarus | 39 (11-75) | 0.47 (0.13-0.89) | 26 (7-52) | 0.25 (0.07-0.48) | -2.37 (-3.19--1.54) |
| Belgium | 12 (3-23) | 0.12 (0.03-0.23) | 5 (1-10) | 0.03 (0.01-0.06) | -3.28 (-4.41--2.14) |
| Belize | 0 (0-0) | 0.1 (0.03-0.2) | 0 (0-0) | 0.07 (0.02-0.13) | -1.34 (-2.2--0.47) |
| Benin | 4 (1-8) | 0.4 (0.11-0.84) | 9 (3-17) | 0.34 (0.09-0.68) | -0.58 (-1.89-0.75) |
| Bermuda | 0 (0-0) | 0.12 (0.03-0.23) | 0 (0-0) | 0.05 (0.01-0.09) | -2.28 (-2.99--1.56) |
| Bhutan | 0 (0-1) | 0.25 (0.07-0.5) | 0 (0-1) | 0.16 (0.04-0.32) | -0.68 (-1.51-0.15) |
| Bolivia (Plurinational State of) | 3 (1-5) | 0.16 (0.04-0.33) | 4 (1-8) | 0.09 (0.02-0.18) | -1.32 (-1.99--0.64) |
| Bosnia and Herzegovina | 12 (3-22) | 0.55 (0.15-1.05) | 12 (3-24) | 0.33 (0.09-0.67) | -0.69 (-1.35--0.01) |
| Botswana | 1 (0-2) | 0.35 (0.09-0.67) | 2 (1-3) | 0.23 (0.06-0.45) | -0.05 (-1.07-0.98) |
| Brazil | 52 (14-98) | 0.12 (0.03-0.23) | 61 (16-119) | 0.04 (0.01-0.09) | -1.99 (-2.38--1.6) |
| Brunei Darussalam | 0 (0-0) | 0.33 (0.09-0.66) | 0 (0-0) | 0.16 (0.04-0.3) | -1.56 (-2.64--0.48) |
| Bulgaria | 30 (8-58) | 0.52 (0.14-0.99) | 29 (8-57) | 0.33 (0.09-0.64) | -0.48 (-1.37-0.42) |
| Burkina Faso | 4 (1-9) | 0.2 (0.06-0.41) | 9 (3-19) | 0.2 (0.06-0.4) | 0.09 (-1.09-1.3) |
| Burundi | 7 (2-14) | 0.58 (0.16-1.16) | 7 (2-14) | 0.32 (0.09-0.69) | -2.96 (-4.32--1.59) |
| Cabo Verde | 0 (0-1) | 0.2 (0.06-0.41) | 0 (0-1) | 0.15 (0.04-0.3) | -0.47 (-0.87--0.07) |
| Cambodia | 9 (3-18) | 0.4 (0.12-0.77) | 21 (6-42) | 0.32 (0.1-0.66) | 0.05 (-0.79-0.9) |
| Cameroon | 7 (2-13) | 0.32 (0.1-0.64) | 20 (6-39) | 0.33 (0.1-0.66) | 0.1 (-1.19-1.41) |
| Canada | 22 (6-42) | 0.11 (0.03-0.22) | 23 (6-46) | 0.06 (0.02-0.11) | -1.78 (-2.56--0.99) |
| Central African Republic | 2 (1-5) | 0.47 (0.13-0.93) | 4 (1-8) | 0.4 (0.1-0.8) | -0.73 (-2.11-0.66) |
| Chad | 5 (1-10) | 0.34 (0.1-0.76) | 9 (3-18) | 0.36 (0.1-0.74) | -0.59 (-2-0.84) |
| Chile | 9 (3-18) | 0.18 (0.05-0.34) | 9 (2-18) | 0.06 (0.02-0.12) | -1.93 (-2.26--1.6) |
| China | 1489 (448-2946) | 0.36 (0.1-0.73) | 2440 (691-4961) | 0.23 (0.06-0.46) | -0.41 (-0.73--0.08) |
| Colombia | 14 (4-26) | 0.16 (0.05-0.3) | 16 (4-30) | 0.05 (0.01-0.1) | -2.66 (-3.16--2.15) |
| Comoros | 0 (0-1) | 0.4 (0.11-0.79) | 1 (0-1) | 0.27 (0.07-0.54) | -0.65 (-1.58-0.29) |
| Congo | 2 (1-5) | 0.45 (0.13-0.88) | 5 (1-9) | 0.37 (0.1-0.75) | -0.75 (-1.88-0.39) |
| Cook Islands | 0 (0-0) | 0.2 (0.06-0.41) | 0 (0-0) | 0.11 (0.03-0.22) | -0.44 (-0.82--0.06) |
| Costa Rica | 1 (0-2) | 0.1 (0.03-0.19) | 1 (0-3) | 0.04 (0.01-0.08) | -1.93 (-2.42--1.43) |
| C么te d'Ivoire | 6 (2-11) | 0.34 (0.1-0.65) | 16 (5-31) | 0.32 (0.09-0.63) | 0.57 (-0.95-2.11) |
| Croatia | 12 (3-23) | 0.35 (0.09-0.69) | 6 (1-13) | 0.1 (0.03-0.21) | -3.17 (-4.14--2.2) |
| Cuba | 8 (2-16) | 0.16 (0.05-0.32) | 11 (3-21) | 0.1 (0.03-0.19) | -0.63 (-1.13--0.13) |
| Cyprus | 1 (0-1) | 0.19 (0.04-0.39) | 1 (0-1) | 0.05 (0.01-0.11) | -3.54 (-3.86--3.21) |
| Czechia | 42 (11-84) | 0.48 (0.13-0.95) | 13 (3-26) | 0.1 (0.03-0.2) | -4.77 (-5.66--3.87) |
| Democratic People's Republic of Korea | 24 (8-47) | 0.26 (0.08-0.5) | 43 (13-85) | 0.23 (0.07-0.44) | 0.17 (-0.1-0.45) |
| Democratic Republic of the Congo | 28 (8-54) | 0.38 (0.11-0.77) | 56 (15-117) | 0.32 (0.08-0.7) | -0.68 (-1.92-0.58) |
| Denmark | 6 (2-13) | 0.12 (0.03-0.24) | 4 (1-7) | 0.05 (0.01-0.1) | -2.92 (-3.95--1.87) |
| Djibouti | 0 (0-0) | 0.25 (0.07-0.52) | 1 (0-1) | 0.22 (0.07-0.45) | 0.09 (-1.27-1.47) |
| Dominica | 0 (0-0) | 0.24 (0.07-0.47) | 0 (0-0) | 0.18 (0.05-0.34) | -0.4 (-0.67--0.13) |
| Dominican Republic | 2 (0-3) | 0.09 (0.03-0.18) | 3 (1-5) | 0.05 (0.01-0.1) | -0.34 (-0.91-0.23) |
| Ecuador | 3 (1-6) | 0.13 (0.04-0.24) | 4 (1-8) | 0.05 (0.01-0.09) | -2.17 (-2.71--1.63) |
| Egypt | 109 (31-228) | 0.97 (0.26-2.07) | 173 (53-331) | 0.75 (0.21-1.45) | -0.35 (-1.5-0.82) |
| El Salvador | 2 (1-3) | 0.11 (0.03-0.22) | 2 (1-5) | 0.06 (0.02-0.12) | -1.07 (-1.5--0.63) |
| Equatorial Guinea | 0 (0-1) | 0.4 (0.12-0.83) | 1 (0-2) | 0.28 (0.08-0.6) | -1.58 (-2.89--0.26) |
| Eritrea | 2 (1-5) | 0.38 (0.11-0.82) | 4 (1-8) | 0.29 (0.08-0.58) | -0.32 (-1.63-1.02) |
| Estonia | 7 (2-13) | 0.47 (0.13-0.92) | 1 (0-3) | 0.08 (0.02-0.16) | -6.3 (-7.29--5.31) |
| Eswatini | 0 (0-1) | 0.33 (0.09-0.65) | 1 (0-2) | 0.35 (0.09-0.72) | 1.14 (-0.19-2.49) |
| Ethiopia | 23 (7-51) | 0.26 (0.07-0.56) | 34 (11-69) | 0.16 (0.05-0.34) | -1.62 (-3.05--0.16) |
| Fiji | 0 (0-0) | 0.12 (0.04-0.24) | 0 (0-1) | 0.13 (0.04-0.26) | 1.22 (0.52-1.93) |
| Finland | 10 (3-19) | 0.2 (0.05-0.4) | 6 (1-12) | 0.07 (0.02-0.14) | -2.56 (-3.69--1.43) |
| France | 59 (14-119) | 0.1 (0.03-0.2) | 37 (8-77) | 0.03 (0.01-0.07) | -2.73 (-3.88--1.57) |
| Gabon | 1 (0-2) | 0.33 (0.08-0.64) | 1 (0-3) | 0.28 (0.08-0.6) | -0.96 (-1.8--0.12) |
| Gambia | 0 (0-1) | 0.29 (0.08-0.6) | 2 (0-3) | 0.33 (0.08-0.72) | 0.82 (-0.51-2.17) |
| Georgia | 13 (4-24) | 0.34 (0.1-0.64) | 13 (3-25) | 0.33 (0.09-0.64) | 0.36 (-0.44-1.17) |
| Germany | 145 (36-290) | 0.17 (0.04-0.32) | 65 (16-130) | 0.05 (0.01-0.1) | -3.03 (-4.21--1.84) |
| Ghana | 14 (4-28) | 0.47 (0.13-0.94) | 36 (11-71) | 0.41 (0.12-0.84) | -0.09 (-1.16-1) |
| Greece | 25 (6-50) | 0.3 (0.08-0.6) | 15 (3-31) | 0.09 (0.02-0.17) | -3.45 (-4.47--2.42) |
| Greenland | 0 (0-0) | 0.25 (0.07-0.5) | 0 (0-0) | 0.07 (0.02-0.14) | -3.63 (-4.15--3.12) |
| Grenada | 0 (0-0) | 0.3 (0.08-0.58) | 0 (0-0) | 0.11 (0.03-0.21) | -2.58 (-2.94--2.22) |
| Guam | 0 (0-0) | 0.18 (0.05-0.35) | 0 (0-0) | 0.1 (0.03-0.18) | -0.38 (-0.84-0.08) |
| Guatemala | 2 (1-4) | 0.14 (0.04-0.27) | 3 (1-6) | 0.06 (0.02-0.11) | -2.35 (-3.39--1.29) |
| Guinea | 6 (2-12) | 0.36 (0.1-0.77) | 9 (3-19) | 0.35 (0.1-0.72) | -0.23 (-1.24-0.78) |
| Guinea-Bissau | 1 (0-2) | 0.41 (0.12-0.85) | 1 (0-3) | 0.41 (0.11-0.84) | 0 (-1.39-1.4) |
| Guyana | 1 (0-2) | 0.51 (0.14-1) | 1 (0-2) | 0.28 (0.08-0.56) | -0.31 (-1.03-0.43) |
| Haiti | 8 (2-15) | 0.5 (0.13-0.98) | 11 (3-24) | 0.33 (0.09-0.7) | -1.11 (-2.1--0.11) |
| Honduras | 2 (1-4) | 0.2 (0.05-0.38) | 7 (2-14) | 0.22 (0.06-0.44) | 0.65 (-0.32-1.62) |
| Hungary | 37 (10-71) | 0.43 (0.12-0.82) | 15 (4-30) | 0.12 (0.03-0.24) | -3.7 (-4.6--2.79) |
| Iceland | 0 (0-0) | 0.11 (0.03-0.21) | 0 (0-0) | 0.04 (0.01-0.08) | -2.81 (-3.48--2.13) |
| India | 480 (156-945) | 0.22 (0.07-0.45) | 951 (288-1844) | 0.16 (0.05-0.31) | -0.45 (-1.04-0.15) |
| Indonesia | 214 (64-416) | 0.43 (0.13-0.86) | 491 (143-962) | 0.44 (0.12-0.87) | 0.75 (0.14-1.36) |
| Iran (Islamic Republic of) | 40 (12-74) | 0.33 (0.09-0.64) | 60 (18-110) | 0.16 (0.05-0.3) | -1.36 (-2.1--0.61) |
| Iraq | 25 (8-46) | 0.61 (0.18-1.13) | 46 (15-85) | 0.39 (0.11-0.74) | -1.55 (-2.46--0.63) |
| Ireland | 4 (1-8) | 0.17 (0.04-0.33) | 2 (0-4) | 0.04 (0.01-0.08) | -4.46 (-5.02--3.89) |
| Israel | 2 (0-4) | 0.08 (0.02-0.15) | 2 (0-4) | 0.02 (0.01-0.05) | -3.39 (-3.74--3.04) |
| Italy | 76 (19-152) | 0.14 (0.03-0.27) | 45 (9-95) | 0.04 (0.01-0.08) | -3.26 (-4.58--1.91) |
| Jamaica | 2 (1-4) | 0.22 (0.06-0.43) | 2 (1-5) | 0.14 (0.04-0.26) | -0.77 (-1.11--0.44) |
| Japan | 131 (33-259) | 0.14 (0.04-0.27) | 107 (24-218) | 0.04 (0.01-0.08) | -2.56 (-3.83--1.27) |
| Jordan | 3 (1-6) | 0.52 (0.14-0.99) | 6 (2-11) | 0.18 (0.05-0.37) | -3.52 (-4.77--2.25) |
| Kazakhstan | 41 (11-76) | 0.53 (0.15-0.99) | 32 (9-61) | 0.33 (0.09-0.62) | -2.23 (-2.59--1.86) |
| Kenya | 9 (3-19) | 0.23 (0.07-0.5) | 24 (7-48) | 0.21 (0.06-0.44) | 0.49 (-0.81-1.8) |
| Kiribati | 0 (0-0) | 0.26 (0.07-0.5) | 0 (0-0) | 0.25 (0.08-0.49) | 0.09 (-0.69-0.87) |
| Kuwait | 0 (0-1) | 0.15 (0.04-0.28) | 1 (0-2) | 0.06 (0.02-0.11) | -2.09 (-3.1--1.06) |
| Kyrgyzstan | 9 (3-18) | 0.53 (0.16-1.01) | 8 (2-15) | 0.29 (0.09-0.53) | -2.75 (-3.23--2.27) |
| Lao People's Democratic Republic | 6 (2-13) | 0.62 (0.18-1.2) | 8 (2-16) | 0.37 (0.1-0.71) | -1.54 (-2.43--0.63) |
| Latvia | 11 (3-22) | 0.47 (0.13-0.9) | 6 (2-13) | 0.2 (0.05-0.4) | -2.34 (-3.41--1.27) |
| Lebanon | 1 (0-3) | 0.14 (0.04-0.29) | 2 (1-4) | 0.07 (0.02-0.13) | -1.95 (-2.23--1.66) |
| Lesotho | 1 (0-2) | 0.25 (0.07-0.5) | 3 (1-5) | 0.42 (0.11-0.86) | 2.67 (1.93-3.41) |
| Liberia | 2 (1-4) | 0.37 (0.1-0.73) | 3 (1-7) | 0.35 (0.1-0.74) | -0.74 (-1.99-0.53) |
| Libya | 2 (1-3) | 0.19 (0.05-0.36) | 5 (2-11) | 0.19 (0.05-0.39) | 1.29 (0.4-2.19) |
| Lithuania | 9 (2-16) | 0.31 (0.08-0.58) | 6 (1-12) | 0.14 (0.04-0.28) | -1.44 (-2.4--0.46) |
| Luxembourg | 1 (0-2) | 0.22 (0.06-0.43) | 0 (0-1) | 0.04 (0.01-0.07) | -5.2 (-6.06--4.33) |
| Madagascar | 11 (3-21) | 0.44 (0.13-0.89) | 21 (7-43) | 0.39 (0.11-0.78) | -0.66 (-2-0.71) |
| Malawi | 7 (2-13) | 0.35 (0.09-0.69) | 12 (4-24) | 0.32 (0.09-0.63) | -0.6 (-1.91-0.73) |
| Malaysia | 12 (3-22) | 0.25 (0.07-0.49) | 24 (7-46) | 0.18 (0.05-0.36) | -0.01 (-0.71-0.69) |
| Maldives | 0 (0-0) | 0.48 (0.14-0.9) | 0 (0-0) | 0.16 (0.04-0.31) | -2.66 (-3.74--1.57) |
| Mali | 7 (2-15) | 0.38 (0.11-0.81) | 13 (4-27) | 0.31 (0.09-0.68) | -0.93 (-2.25-0.41) |
| Malta | 0 (0-1) | 0.12 (0.03-0.23) | 0 (0-0) | 0.03 (0.01-0.07) | -3.03 (-3.88--2.17) |
| Marshall Islands | 0 (0-0) | 0.31 (0.08-0.61) | 0 (0-0) | 0.29 (0.08-0.61) | 0.51 (-0.7-1.73) |
| Mauritania | 2 (1-4) | 0.36 (0.1-0.74) | 3 (1-5) | 0.26 (0.08-0.54) | -1.21 (-2.28--0.13) |
| Mauritius | 0 (0-1) | 0.13 (0.04-0.25) | 0 (0-1) | 0.05 (0.02-0.1) | -2.56 (-2.98--2.15) |
| Mexico | 30 (9-55) | 0.15 (0.04-0.28) | 41 (12-79) | 0.06 (0.02-0.12) | -1.59 (-2.21--0.97) |
| Micronesia (Federated States of) | 0 (0-0) | 0.35 (0.1-0.69) | 0 (0-0) | 0.29 (0.09-0.58) | -0.05 (-0.87-0.78) |
| Monaco | 0 (0-0) | 0.12 (0.03-0.25) | 0 (0-0) | 0.05 (0.01-0.09) | -3.15 (-4.7--1.58) |
| Mongolia | 1 (0-2) | 0.16 (0.05-0.3) | 2 (1-3) | 0.12 (0.04-0.23) | -0.62 (-1.33-0.09) |
| Montenegro | 0 (0-1) | 0.13 (0.04-0.26) | 1 (0-2) | 0.19 (0.05-0.39) | 2.15 (1.62-2.67) |
| Morocco | 34 (10-67) | 0.47 (0.13-0.93) | 65 (17-133) | 0.38 (0.1-0.78) | 0.23 (-0.3-0.76) |
| Mozambique | 10 (3-18) | 0.34 (0.1-0.65) | 18 (5-36) | 0.32 (0.09-0.65) | -0.18 (-1.48-1.15) |
| Myanmar | 60 (18-113) | 0.5 (0.14-0.98) | 74 (22-144) | 0.28 (0.08-0.56) | -1.47 (-1.97--0.97) |
| Namibia | 1 (0-3) | 0.42 (0.12-0.84) | 2 (1-4) | 0.32 (0.09-0.65) | -0.79 (-1.8-0.24) |
| Nauru | 0 (0-0) | 0.42 (0.13-0.85) | 0 (0-0) | 0.42 (0.13-0.84) | 0.36 (-0.61-1.35) |
| Nepal | 14 (4-27) | 0.31 (0.09-0.62) | 17 (5-34) | 0.14 (0.04-0.29) | -1.97 (-2.82--1.12) |
| Netherlands | 12 (3-24) | 0.1 (0.02-0.19) | 9 (2-19) | 0.04 (0.01-0.08) | -2.21 (-3.15--1.26) |
| New Zealand | 3 (1-6) | 0.13 (0.03-0.25) | 3 (1-6) | 0.05 (0.01-0.11) | -2.32 (-2.99--1.64) |
| Nicaragua | 1 (0-2) | 0.11 (0.03-0.21) | 2 (0-3) | 0.06 (0.02-0.12) | -0.88 (-1.66--0.09) |
| Niger | 4 (1-9) | 0.32 (0.09-0.72) | 11 (3-23) | 0.28 (0.07-0.61) | -0.42 (-2.01-1.19) |
| Nigeria | 66 (19-142) | 0.32 (0.09-0.71) | 110 (32-213) | 0.25 (0.07-0.49) | -1.42 (-2.71--0.11) |
| Niue | 0 (0-0) | 0.28 (0.08-0.54) | 0 (0-0) | 0.22 (0.06-0.44) | -0.66 (-1.03--0.28) |
| North Macedonia | 6 (2-12) | 0.69 (0.18-1.34) | 9 (2-17) | 0.58 (0.14-1.17) | -0.23 (-0.53-0.07) |
| Northern Mariana Islands | 0 (0-0) | 0.2 (0.06-0.39) | 0 (0-0) | 0.13 (0.04-0.25) | 0.34 (-0.71-1.4) |
| Norway | 4 (1-7) | 0.08 (0.02-0.15) | 2 (0-3) | 0.03 (0.01-0.05) | -3.78 (-4.87--2.68) |
| Oman | 1 (0-2) | 0.39 (0.11-0.77) | 2 (1-4) | 0.22 (0.06-0.42) | -0.96 (-2.12-0.21) |
| Pakistan | 70 (22-135) | 0.29 (0.09-0.58) | 150 (50-298) | 0.27 (0.09-0.54) | -0.56 (-1.63-0.51) |
| Palau | 0 (0-0) | 0.28 (0.08-0.54) | 0 (0-0) | 0.26 (0.08-0.53) | 0.74 (0.33-1.16) |
| Palestine | 3 (1-5) | 0.62 (0.17-1.23) | 4 (1-7) | 0.32 (0.09-0.62) | -2.12 (-3.3--0.93) |
| Panama | 1 (0-1) | 0.07 (0.02-0.15) | 1 (0-2) | 0.04 (0.01-0.08) | -1.2 (-1.55--0.84) |
| Papua New Guinea | 2 (1-5) | 0.31 (0.09-0.61) | 6 (2-11) | 0.26 (0.07-0.52) | -0.56 (-1.81-0.71) |
| Paraguay | 2 (0-3) | 0.15 (0.04-0.3) | 2 (0-3) | 0.06 (0.01-0.12) | -2.47 (-3.02--1.92) |
| Peru | 6 (2-12) | 0.1 (0.03-0.19) | 10 (3-20) | 0.06 (0.02-0.11) | -1.46 (-1.96--0.95) |
| Philippines | 33 (10-62) | 0.25 (0.07-0.5) | 95 (30-185) | 0.22 (0.07-0.45) | 0.76 (-0.06-1.59) |
| Poland | 111 (30-215) | 0.42 (0.12-0.82) | 63 (16-124) | 0.14 (0.04-0.27) | -3.04 (-3.79--2.29) |
| Portugal | 28 (7-56) | 0.35 (0.09-0.7) | 11 (2-23) | 0.06 (0.01-0.12) | -4.93 (-5.99--3.87) |
| Puerto Rico | 1 (0-2) | 0.07 (0.02-0.13) | 1 (0-2) | 0.02 (0.01-0.04) | -2.44 (-3.13--1.75) |
| Qatar | 0 (0-0) | 0.24 (0.06-0.49) | 0 (0-1) | 0.08 (0.02-0.17) | -3.23 (-4.9--1.54) |
| Republic of Korea | 50 (14-97) | 0.34 (0.09-0.67) | 25 (7-52) | 0.05 (0.01-0.1) | -5.31 (-5.82--4.79) |
| Republic of Moldova | 8 (2-15) | 0.32 (0.09-0.63) | 7 (2-14) | 0.2 (0.06-0.37) | -0.46 (-1.07-0.16) |
| Romania | 68 (18-133) | 0.49 (0.13-0.95) | 61 (16-118) | 0.25 (0.07-0.47) | -1.48 (-2.28--0.67) |
| Russian Federation | 756 (210-1444) | 0.65 (0.18-1.23) | 505 (131-994) | 0.32 (0.09-0.63) | -2.72 (-3.5--1.94) |
| Rwanda | 9 (3-17) | 0.6 (0.18-1.19) | 9 (3-18) | 0.26 (0.07-0.55) | -3.22 (-4.45--1.98) |
| Saint Kitts and Nevis | 0 (0-0) | 0.41 (0.12-0.79) | 0 (0-0) | 0.17 (0.04-0.32) | -2.69 (-2.86--2.53) |
| Saint Lucia | 0 (0-0) | 0.45 (0.13-0.88) | 0 (0-0) | 0.17 (0.05-0.33) | -2.38 (-2.86--1.89) |
| Saint Vincent and the Grenadines | 0 (0-0) | 0.34 (0.09-0.64) | 0 (0-0) | 0.17 (0.05-0.32) | -1.38 (-1.68--1.07) |
| Samoa | 0 (0-0) | 0.27 (0.08-0.51) | 0 (0-0) | 0.24 (0.07-0.46) | 0.09 (-0.58-0.77) |
| San Marino | 0 (0-0) | 0.1 (0.03-0.21) | 0 (0-0) | 0.04 (0.01-0.09) | -2.2 (-3.4--1) |
| Sao Tome and Principe | 0 (0-0) | 0.33 (0.1-0.62) | 0 (0-0) | 0.36 (0.1-0.67) | 0 (-0.93-0.94) |
| Saudi Arabia | 13 (4-25) | 0.5 (0.14-0.98) | 31 (10-56) | 0.33 (0.1-0.64) | -0.77 (-1.88-0.35) |
| Senegal | 5 (1-9) | 0.31 (0.09-0.63) | 9 (3-18) | 0.24 (0.07-0.49) | -0.42 (-1.55-0.73) |
| Serbia | 35 (10-67) | 0.7 (0.19-1.33) | 36 (9-73) | 0.36 (0.09-0.72) | -1.5 (-2.21--0.79) |
| Seychelles | 0 (0-0) | 0.21 (0.05-0.4) | 0 (0-0) | 0.16 (0.04-0.31) | 0.07 (-0.09-0.23) |
| Sierra Leone | 4 (1-7) | 0.39 (0.11-0.78) | 7 (2-15) | 0.4 (0.11-0.82) | 0.08 (-1.04-1.21) |
| Singapore | 3 (1-5) | 0.24 (0.07-0.47) | 2 (0-3) | 0.04 (0.01-0.07) | -4.99 (-5.29--4.68) |
| Slovakia | 12 (3-23) | 0.34 (0.09-0.66) | 9 (2-17) | 0.15 (0.04-0.3) | -2.13 (-2.79--1.46) |
| Slovenia | 4 (1-7) | 0.25 (0.07-0.49) | 2 (0-4) | 0.06 (0.02-0.12) | -3.62 (-4.63--2.59) |
| Solomon Islands | 0 (0-0) | 0.39 (0.12-0.79) | 1 (0-1) | 0.36 (0.11-0.72) | 0.36 (-0.92-1.67) |
| Somalia | 4 (1-8) | 0.31 (0.08-0.71) | 8 (2-17) | 0.25 (0.07-0.56) | -0.77 (-2.31-0.78) |
| South Africa | 25 (8-49) | 0.21 (0.06-0.41) | 46 (13-88) | 0.18 (0.05-0.35) | 0.07 (-0.44-0.58) |
| South Sudan | 3 (1-6) | 0.28 (0.08-0.58) | 4 (1-8) | 0.24 (0.07-0.49) | -0.83 (-2.38-0.73) |
| Spain | 56 (13-111) | 0.17 (0.04-0.33) | 26 (6-54) | 0.04 (0.01-0.07) | -3.99 (-5.11--2.85) |
| Sri Lanka | 17 (5-32) | 0.38 (0.1-0.74) | 37 (10-73) | 0.26 (0.07-0.52) | 0.51 (0.06-0.97) |
| Sudan | 30 (8-64) | 0.66 (0.17-1.38) | 45 (13-93) | 0.46 (0.12-0.98) | -1.39 (-2.49--0.28) |
| Suriname | 0 (0-0) | 0.16 (0.05-0.31) | 0 (0-0) | 0.06 (0.02-0.12) | -2.42 (-2.84--1.99) |
| Sweden | 12 (3-25) | 0.12 (0.03-0.24) | 7 (2-14) | 0.05 (0.01-0.1) | -2.87 (-4.12--1.6) |
| Switzerland | 8 (2-16) | 0.11 (0.03-0.22) | 4 (1-9) | 0.03 (0.01-0.07) | -3.27 (-4.43--2.09) |
| Syrian Arab Republic | 11 (4-21) | 0.45 (0.13-0.87) | 16 (5-32) | 0.29 (0.08-0.58) | -0.99 (-2.18-0.22) |
| Taiwan (Province of China) | 5 (2-11) | 0.08 (0.02-0.16) | 4 (1-8) | 0.02 (0-0.04) | -2.97 (-3.47--2.47) |
| Tajikistan | 7 (2-12) | 0.43 (0.13-0.81) | 10 (3-19) | 0.35 (0.1-0.71) | -1.18 (-2.11--0.25) |
| Thailand | 41 (12-79) | 0.22 (0.06-0.44) | 71 (19-142) | 0.12 (0.03-0.24) | -0.91 (-1.33--0.49) |
| Timor-Leste | 0 (0-1) | 0.34 (0.1-0.66) | 1 (0-3) | 0.31 (0.09-0.64) | 0.9 (-0.23-2.03) |
| Togo | 2 (1-5) | 0.39 (0.12-0.81) | 7 (2-14) | 0.34 (0.09-0.7) | 0.1 (-1.19-1.42) |
| Tokelau | 0 (0-0) | 0.33 (0.09-0.67) | 0 (0-0) | 0.24 (0.06-0.48) | -0.56 (-0.76--0.36) |
| Tonga | 0 (0-0) | 0.16 (0.05-0.31) | 0 (0-0) | 0.15 (0.04-0.29) | 0.53 (0.02-1.05) |
| Trinidad and Tobago | 1 (0-1) | 0.14 (0.04-0.27) | 1 (0-1) | 0.05 (0.01-0.1) | -2.49 (-2.84--2.14) |
| Tunisia | 6 (2-11) | 0.24 (0.07-0.5) | 12 (3-26) | 0.18 (0.05-0.38) | 0.16 (-0.28-0.61) |
| Turkey | 60 (17-114) | 0.34 (0.09-0.67) | 80 (22-158) | 0.17 (0.04-0.33) | -1.17 (-1.53--0.81) |
| Turkmenistan | 6 (2-11) | 0.5 (0.15-0.96) | 12 (4-22) | 0.53 (0.16-1.02) | 0.6 (0.01-1.19) |
| Tuvalu | 0 (0-0) | 0.38 (0.11-0.77) | 0 (0-0) | 0.29 (0.08-0.6) | -0.58 (-0.86--0.29) |
| Uganda | 9 (3-17) | 0.28 (0.08-0.56) | 15 (4-31) | 0.19 (0.05-0.41) | -1.75 (-3.22--0.25) |
| Ukraine | 253 (69-492) | 0.54 (0.15-1.06) | 140 (36-282) | 0.28 (0.07-0.56) | -2.46 (-3.28--1.64) |
| United Arab Emirates | 0 (0-1) | 0.3 (0.08-0.58) | 2 (1-4) | 0.46 (0.11-0.94) | 1.69 (-0.52-3.95) |
| United Kingdom | 96 (24-191) | 0.16 (0.04-0.31) | 36 (9-73) | 0.05 (0.01-0.09) | -4.11 (-5.14--3.06) |
| United Republic of Tanzania | 12 (4-23) | 0.22 (0.06-0.44) | 28 (8-57) | 0.23 (0.06-0.47) | 0.14 (-1.09-1.38) |
| United States of America | 107 (28-211) | 0.05 (0.01-0.11) | 91 (23-181) | 0.03 (0.01-0.05) | -2.4 (-3.14--1.66) |
| United States Virgin Islands | 0 (0-0) | 0.11 (0.03-0.22) | 0 (0-0) | 0.03 (0.01-0.07) | -2.11 (-2.65--1.57) |
| Uruguay | 6 (2-11) | 0.24 (0.07-0.47) | 3 (1-6) | 0.08 (0.02-0.15) | -3.4 (-4.15--2.64) |
| Uzbekistan | 25 (8-46) | 0.37 (0.11-0.69) | 41 (13-76) | 0.29 (0.09-0.55) | -0.98 (-1.56--0.4) |
| Vanuatu | 0 (0-0) | 0.33 (0.1-0.66) | 0 (0-0) | 0.29 (0.08-0.55) | 0.03 (-1.09-1.16) |
| Venezuela (Bolivarian Republic of) | 4 (1-8) | 0.08 (0.02-0.17) | 10 (3-19) | 0.06 (0.02-0.12) | -0.28 (-0.87-0.32) |
| Viet Nam | 79 (22-161) | 0.35 (0.1-0.72) | 151 (42-300) | 0.29 (0.08-0.58) | 0.47 (0.1-0.85) |
| Yemen | 17 (5-34) | 0.67 (0.18-1.35) | 41 (11-84) | 0.57 (0.14-1.2) | -0.6 (-1.87-0.69) |
| Zambia | 4 (1-8) | 0.34 (0.09-0.69) | 11 (3-21) | 0.32 (0.09-0.66) | -0.19 (-1.75-1.4) |

Abbreviations: IS,Ischemic stroke,IIPUFAs, insufficient intake of polyunsaturated fatty acids,EAPC, estimated annual percentage change, SDl, Sociodemographic Index; Ul,uncertainty interval. “ EAPC is expressed as 95% CIs.

Table2 The Deaths cases and age-standardized Deaths rate of ischemic stroke attributed to IIPUFAs disease in 1990 and 2021, along with their temporal trend.

|  | Rate per 100 000(95%UI) | | 2021 | | 1990-2021 |
| --- | --- | --- | --- | --- | --- |
|  | 1990 | |  | |  |
|  | Deaths cases | The age-standardized deaths rate | Deaths cases | The age-standardized deaths rate | EAPC |

| **Global** | 277 (76-549) | 0.01 (0-0.03) | 364 (96-736) | 0.01 (0-0.02) | -1.47 (-1.71--1.23) |
| --- | --- | --- | --- | --- | --- |
| **Age** |  |  |  |  |  |
| 50-54 years | 7 (3-13) | 0.01 (0-0.01) | 8 (3-14) | 0 (0-0.01) | -2.27 (-2.43--2.11) |
| 55-59 years | 11 (4-20) | 0.01 (0-0.02) | 13 (4-23) | 0.01 (0-0.01) | -2.46 (-2.61--2.31) |
| 60-64 years | 21 (6-39) | 0.03 (0.01-0.05) | 22 (7-41) | 0.01 (0-0.03) | -2.45 (-2.61--2.28) |
| 65-69 years | 26 (7-50) | 0.04 (0.01-0.08) | 32 (9-61) | 0.02 (0.01-0.04) | -2.47 (-2.71--2.23) |
| 70-74 years | 37 (10-74) | 0.08 (0.02-0.16) | 48 (13-96) | 0.04 (0.01-0.09) | -2.32 (-2.55--2.09) |
| 75-79 years | 49 (12-101) | 0.13 (0.03-0.28) | 50 (13-104) | 0.07 (0.02-0.14) | -2.2 (-2.36--2.05) |
| 80-84 years | 54 (12-114) | 0.24 (0.06-0.52) | 69 (15-145) | 0.13 (0.03-0.29) | -2.12 (-2.2--2.04) |
| 85-89 years | 40 (9-85) | 0.4 (0.09-0.85) | 60 (13-129) | 0.21 (0.05-0.45) | -2.24 (-2.37--2.11) |
| 90-94 years | 17 (4-36) | 0.57 (0.12-1.2) | 37 (8-80) | 0.31 (0.07-0.66) | -2.29 (-2.46--2.13) |
| 95+ years | 5 (1-12) | 0.72 (0.15-1.52) | 16 (3-34) | 0.41 (0.09-0.87) | -2.14 (-2.28--1.99) |
| **SDI region** |  |  |  |  |  |
| High SDI | 58 (14-118) | 0.01 (0-0.02) | 40 (9-83) | 0 (0-0.01) | -4.1 (-4.23--3.97) |
| High-middle SDI | 112 (30-222) | 0.02 (0.01-0.04) | 118 (30-245) | 0.01 (0-0.02) | -3.05 (-3.31--2.78) |
| Middle SDI | 61 (17-119) | 0.01 (0-0.03) | 116 (31-238) | 0.01 (0-0.02) | -1.61 (-1.7--1.51) |
| Low-middle SDI | 34 (10-66) | 0.01 (0-0.03) | 68 (19-139) | 0.01 (0-0.02) | -1 (-1.05--0.94) |
| Low SDI | 12 (4-25) | 0.01 (0-0.03) | 23 (6-48) | 0.01 (0-0.03) | -0.85 (-0.93--0.78) |
| **GBD region** |  |  |  |  |  |
| Advanced Health System | 138 (35-277) | 0.01 (0-0.03) | 98 (22-201) | 0 (0-0.01) | -3.02 (-3.97--2.06) |
| Africa | 20 (6-39) | 0.02 (0.01-0.04) | 37 (10-74) | 0.01 (0-0.03) | -0.61 (-2.02-0.82) |
| African Region | 13 (4-26) | 0.01 (0-0.03) | 25 (6-51) | 0.01 (0-0.03) | -0.56 (-2.05-0.95) |
| America | 16 (4-32) | 0 (0-0.01) | 17 (4-35) | 0 (0-0) | -1.85 (-2.19--1.5) |
| Andean Latin America | 1 (0-1) | 0.01 (0-0.01) | 1 (0-2) | 0 (0-0.01) | -1.51 (-2.13--0.89) |
| Asia | 120 (34-234) | 0.01 (0-0.03) | 228 (62-468) | 0.01 (0-0.02) | -0.48 (-0.97-0.01) |
| Australasia | 1 (0-2) | 0.01 (0-0.02) | 1 (0-2) | 0 (0-0.01) | -2.95 (-3.87--2.02) |
| Basic Health System | 96 (26-193) | 0.02 (0-0.03) | 179 (48-369) | 0.01 (0-0.02) | -0.31 (-0.78-0.16) |
| Caribbean | 1 (0-2) | 0.01 (0-0.02) | 2 (0-3) | 0.01 (0-0.01) | -0.95 (-1.21--0.68) |
| Central Africa | 2 (1-4) | 0.02 (0-0.04) | 4 (1-8) | 0.02 (0-0.03) | -0.66 (-2.23-0.95) |
| Central Asia | 5 (1-9) | 0.02 (0-0.03) | 5 (1-11) | 0.01 (0-0.03) | -1.32 (-1.72--0.92) |
| Central Europe | 21 (6-42) | 0.03 (0.01-0.05) | 17 (4-34) | 0.01 (0-0.02) | -2.04 (-2.87--1.2) |
| Central Latin America | 2 (1-5) | 0.01 (0-0.01) | 4 (1-8) | 0 (0-0.01) | -1.48 (-2.18--0.77) |
| Central Sub-Saharan Africa | 1 (0-3) | 0.02 (0-0.03) | 3 (1-7) | 0.01 (0-0.03) | -0.59 (-2.21-1.05) |
| Commonwealth High Income | 9 (2-18) | 0.01 (0-0.02) | 5 (1-10) | 0 (0-0) | -3.81 (-4.88--2.73) |
| Commonwealth Low Income | 5 (2-11) | 0.02 (0-0.04) | 13 (3-27) | 0.01 (0-0.03) | 0.19 (-1.3-1.71) |
| Commonwealth Middle Income | 27 (8-55) | 0.01 (0-0.02) | 57 (16-113) | 0.01 (0-0.02) | -0.38 (-1.31-0.56) |
| East Asia | 58 (16-117) | 0.02 (0-0.03) | 110 (29-233) | 0.01 (0-0.02) | -0.18 (-0.6-0.24) |
| East Asia & Pacific - WB | 87 (23-174) | 0.02 (0-0.03) | 158 (42-330) | 0.01 (0-0.02) | -0.35 (-0.73-0.02) |
| Eastern Africa | 4 (1-8) | 0.01 (0-0.03) | 7 (2-14) | 0.01 (0-0.02) | -1.06 (-2.68-0.59) |
| Eastern Europe | 58 (15-115) | 0.03 (0.01-0.07) | 41 (10-82) | 0.02 (0-0.03) | -2.51 (-3.34--1.68) |
| Eastern Mediterranean Region | 15 (4-30) | 0.02 (0.01-0.05) | 27 (8-52) | 0.02 (0-0.03) | -0.9 (-2.1-0.31) |
| Eastern Sub-Saharan Africa | 4 (1-8) | 0.01 (0-0.03) | 7 (2-14) | 0.01 (0-0.02) | -0.78 (-2.46-0.94) |
| Europe | 121 (30-240) | 0.02 (0-0.04) | 82 (19-168) | 0.01 (0-0.01) | -2.8 (-3.77--1.82) |
| Europe & Central Asia - WB | 124 (31-247) | 0.02 (0-0.04) | 85 (20-174) | 0.01 (0-0.01) | -2.78 (-3.69--1.86) |
| European Region | 124 (32-247) | 0.02 (0-0.04) | 86 (20-175) | 0.01 (0-0.01) | -2.78 (-3.68--1.87) |
| High-income Asia Pacific | 10 (2-20) | 0.01 (0-0.02) | 9 (2-19) | 0 (0-0) | -3.36 (-4.73--1.98) |
| High-income North America | 7 (2-14) | 0 (0-0.01) | 6 (1-13) | 0 (0-0) | -2.44 (-3.44--1.43) |
| Latin America & Caribbean - WB | 9 (2-17) | 0.01 (0-0.02) | 11 (3-22) | 0 (0-0.01) | -1.83 (-2.29--1.38) |
| Limited Health System | 38 (11-77) | 0.01 (0-0.03) | 80 (22-161) | 0.01 (0-0.02) | -0.49 (-1.54-0.57) |
| Middle East & North Africa - WB | 12 (3-23) | 0.02 (0.01-0.05) | 21 (6-40) | 0.02 (0-0.03) | -0.82 (-1.87-0.25) |
| Minimal Health System | 4 (1-8) | 0.02 (0-0.04) | 7 (2-16) | 0.02 (0-0.03) | -0.73 (-2.3-0.87) |
| North Africa and Middle East | 16 (4-31) | 0.02 (0.01-0.05) | 27 (7-54) | 0.01 (0-0.03) | -0.83 (-1.79-0.14) |
| North America | 7 (2-14) | 0 (0-0.01) | 6 (1-13) | 0 (0-0) | -2.44 (-3.44--1.42) |
| Northern Africa | 7 (2-14) | 0.03 (0.01-0.07) | 12 (3-24) | 0.02 (0.01-0.04) | -0.33 (-1.4-0.76) |
| Oceania | 0 (0-0) | 0.01 (0-0.03) | 0 (0-1) | 0.01 (0-0.02) | -0.27 (-1.75-1.23) |
| Region of the Americas | 16 (4-32) | 0 (0-0.01) | 17 (4-35) | 0 (0-0) | -1.85 (-2.19--1.5) |
| South-East Asia Region | 34 (10-67) | 0.01 (0-0.03) | 77 (22-160) | 0.01 (0-0.02) | 0 (-0.83-0.85) |
| South Asia | 24 (7-49) | 0.01 (0-0.02) | 54 (15-111) | 0.01 (0-0.02) | -0.2 (-1.12-0.73) |
| South Asia - WB | 26 (8-53) | 0.01 (0-0.03) | 57 (16-117) | 0.01 (0-0.02) | -0.22 (-1.14-0.72) |
| Southeast Asia | 18 (5-36) | 0.02 (0-0.03) | 41 (11-80) | 0.01 (0-0.03) | 0.39 (-0.35-1.13) |
| Southern Africa | 2 (1-4) | 0.01 (0-0.02) | 5 (1-10) | 0.01 (0-0.02) | 0.43 (-0.76-1.63) |
| Southern Latin America | 2 (1-5) | 0.01 (0-0.02) | 2 (0-3) | 0 (0-0.01) | -2.56 (-2.98--2.13) |
| Southern Sub-Saharan Africa | 1 (0-2) | 0.01 (0-0.02) | 3 (1-5) | 0.01 (0-0.02) | 1.03 (0.17-1.89) |
| Sub-Saharan Africa - WB | 13 (4-27) | 0.01 (0-0.03) | 24 (7-50) | 0.01 (0-0.03) | -0.66 (-2.17-0.86) |
| Tropical Latin America | 3 (1-5) | 0.01 (0-0.01) | 3 (1-7) | 0 (0-0) | -1.87 (-2.36--1.38) |
| Western Africa | 5 (1-11) | 0.02 (0-0.04) | 9 (2-18) | 0.01 (0-0.03) | -0.89 (-2.44-0.68) |
| Western Europe | 38 (9-78) | 0.01 (0-0.02) | 19 (4-41) | 0 (0-0) | -3.69 (-5.02--2.35) |
| Western Pacific Region | 75 (20-150) | 0.02 (0-0.03) | 132 (35-278) | 0.01 (0-0.02) | -0.47 (-0.85--0.09) |
| Western Sub-Saharan Africa | 6 (1-12) | 0.02 (0-0.04) | 10 (3-20) | 0.01 (0-0.03) | -0.82 (-2.37-0.75) |
| World Bank High Income | 75 (18-153) | 0.01 (0-0.02) | 49 (10-101) | 0 (0-0.01) | -3.27 (-4.35--2.17) |
| World Bank Low Income | 9 (3-18) | 0.02 (0-0.03) | 17 (4-35) | 0.01 (0-0.03) | -0.96 (-2.38-0.47) |
| World Bank Lower Middle Income | 70 (20-140) | 0.02 (0-0.04) | 128 (35-261) | 0.01 (0-0.02) | -0.71 (-1.62-0.22) |
| World Bank Upper Middle Income | 122 (33-243) | 0.02 (0-0.04) | 171 (45-357) | 0.01 (0-0.02) | -1.39 (-1.75--1.02) |
| **Countries** |  |  |  |  |  |
| Afghanistan | 1 (0-2) | 0.03 (0.01-0.07) | 1 (0-3) | 0.03 (0.01-0.06) | -1.61 (-2.99--0.22) |
| Albania | 0 (0-0) | 0.01 (0-0.02) | 0 (0-0) | 0.01 (0-0.02) | 2.17 (1.65-2.68) |
| Algeria | 1 (0-2) | 0.03 (0.01-0.06) | 2 (0-4) | 0.02 (0-0.04) | -0.14 (-1.44-1.18) |
| American Samoa | 0 (0-0) | 0.01 (0-0.02) | 0 (0-0) | 0.01 (0-0.02) | 0.93 (-0.15-2.02) |
| Andorra | 0 (0-0) | 0 (0-0.01) | 0 (0-0) | 0 (0-0) | -0.44 (-1.28-0.42) |
| Angola | 0 (0-0) | 0.01 (0-0.02) | 0 (0-1) | 0.01 (0-0.02) | -0.58 (-2.49-1.37) |
| Antigua and Barbuda | 0 (0-0) | 0 (0-0.01) | 0 (0-0) | 0 (0-0.01) | -1.14 (-1.39--0.89) |
| Argentina | 2 (0-3) | 0.01 (0-0.02) | 1 (0-2) | 0 (0-0.01) | -2.69 (-3.13--2.25) |
| Armenia | 0 (0-0) | 0.02 (0-0.03) | 0 (0-1) | 0.01 (0-0.02) | -1.39 (-1.84--0.94) |
| Australia | 1 (0-2) | 0.01 (0-0.02) | 1 (0-2) | 0 (0-0.01) | -3.09 (-4.03--2.14) |
| Austria | 1 (0-2) | 0.01 (0-0.02) | 0 (0-1) | 0 (0-0) | -5.18 (-6.5--3.85) |
| Azerbaijan | 0 (0-1) | 0.01 (0-0.02) | 0 (0-1) | 0.01 (0-0.02) | -0.08 (-0.48-0.32) |
| Bahamas | 0 (0-0) | 0 (0-0.01) | 0 (0-0) | 0 (0-0.01) | 0.29 (-0.15-0.73) |
| Bahrain | 0 (0-0) | 0.02 (0-0.03) | 0 (0-0) | 0.01 (0-0.02) | -2.17 (-4.03--0.28) |
| Bangladesh | 3 (1-7) | 0.02 (0.01-0.04) | 9 (2-19) | 0.02 (0-0.04) | 0.66 (-0.78-2.12) |
| Barbados | 0 (0-0) | 0.01 (0-0.02) | 0 (0-0) | 0.01 (0-0.01) | -1.08 (-1.79--0.36) |
| Belarus | 2 (0-4) | 0.02 (0.01-0.04) | 1 (0-3) | 0.01 (0-0.03) | -1.99 (-2.9--1.08) |
| Belgium | 1 (0-2) | 0.01 (0-0.02) | 0 (0-1) | 0 (0-0) | -3.62 (-4.95--2.28) |
| Belize | 0 (0-0) | 0.01 (0-0.01) | 0 (0-0) | 0 (0-0.01) | -1.37 (-2.32--0.41) |
| Benin | 0 (0-0) | 0.02 (0.01-0.04) | 0 (0-1) | 0.02 (0-0.04) | -0.48 (-2-1.08) |
| Bermuda | 0 (0-0) | 0.01 (0-0.01) | 0 (0-0) | 0 (0-0) | -2.32 (-3.14--1.48) |
| Bhutan | 0 (0-0) | 0.01 (0-0.02) | 0 (0-0) | 0.01 (0-0.02) | -0.2 (-1.21-0.83) |
| Bolivia (Plurinational State of) | 0 (0-0) | 0.01 (0-0.02) | 0 (0-0) | 0 (0-0.01) | -0.95 (-1.81--0.09) |
| Bosnia and Herzegovina | 1 (0-1) | 0.03 (0.01-0.06) | 1 (0-1) | 0.02 (0-0.04) | -0.24 (-0.95-0.48) |
| Botswana | 0 (0-0) | 0.02 (0-0.04) | 0 (0-0) | 0.01 (0-0.02) | -0.12 (-1.41-1.18) |
| Brazil | 2 (1-5) | 0.01 (0-0.01) | 3 (1-7) | 0 (0-0) | -1.86 (-2.35--1.37) |
| Brunei Darussalam | 0 (0-0) | 0.02 (0-0.03) | 0 (0-0) | 0.01 (0-0.02) | -1.52 (-3.03-0.02) |
| Bulgaria | 2 (0-3) | 0.03 (0.01-0.06) | 2 (0-4) | 0.02 (0.01-0.04) | -0.03 (-0.9-0.86) |
| Burkina Faso | 0 (0-0) | 0.01 (0-0.02) | 0 (0-1) | 0.01 (0-0.02) | 0.63 (-0.92-2.2) |
| Burundi | 0 (0-1) | 0.03 (0.01-0.06) | 0 (0-1) | 0.02 (0-0.03) | -3.08 (-4.72--1.42) |
| Cabo Verde | 0 (0-0) | 0.01 (0-0.02) | 0 (0-0) | 0.01 (0-0.02) | 0.1 (-0.26-0.46) |
| Cambodia | 0 (0-1) | 0.02 (0.01-0.04) | 1 (0-2) | 0.02 (0-0.04) | 0.38 (-0.75-1.53) |
| Cameroon | 0 (0-0) | 0.01 (0-0.03) | 1 (0-2) | 0.02 (0-0.03) | 0.27 (-1.38-1.95) |
| Canada | 1 (0-2) | 0.01 (0-0.01) | 1 (0-3) | 0 (0-0) | -2.46 (-3.47--1.43) |
| Central African Republic | 0 (0-0) | 0.02 (0.01-0.05) | 0 (0-0) | 0.02 (0-0.04) | -0.67 (-2.45-1.13) |
| Chad | 0 (0-0) | 0.02 (0-0.04) | 0 (0-1) | 0.02 (0-0.04) | -0.63 (-2.31-1.07) |
| Chile | 0 (0-1) | 0.01 (0-0.02) | 1 (0-1) | 0 (0-0.01) | -1.83 (-2.22--1.44) |
| China | 57 (15-115) | 0.02 (0-0.03) | 108 (28-229) | 0.01 (0-0.02) | -0.18 (-0.6-0.25) |
| Colombia | 1 (0-1) | 0.01 (0-0.02) | 1 (0-2) | 0 (0-0) | -2.5 (-3.08--1.91) |
| Comoros | 0 (0-0) | 0.02 (0-0.04) | 0 (0-0) | 0.01 (0-0.03) | -0.5 (-1.71-0.72) |
| Congo | 0 (0-0) | 0.02 (0.01-0.04) | 0 (0-0) | 0.02 (0-0.04) | -0.76 (-2.21-0.71) |
| Cook Islands | 0 (0-0) | 0.01 (0-0.02) | 0 (0-0) | 0 (0-0.01) | -0.56 (-1.1--0.03) |
| Costa Rica | 0 (0-0) | 0.01 (0-0.01) | 0 (0-0) | 0 (0-0) | -1.73 (-2.31--1.16) |
| Côte d'Ivoire | 0 (0-0) | 0.02 (0-0.03) | 1 (0-1) | 0.02 (0-0.03) | 0.94 (-1.03-2.96) |
| Croatia | 1 (0-1) | 0.02 (0.01-0.04) | 0 (0-1) | 0.01 (0-0.01) | -2.82 (-3.83--1.8) |
| Cuba | 0 (0-1) | 0.01 (0-0.02) | 1 (0-1) | 0.01 (0-0.01) | -0.38 (-0.96-0.21) |
| Cyprus | 0 (0-0) | 0.01 (0-0.03) | 0 (0-0) | 0 (0-0.01) | -3.4 (-3.79--3.02) |
| Czechia | 3 (1-5) | 0.03 (0.01-0.06) | 1 (0-2) | 0.01 (0-0.01) | -5.16 (-6.15--4.15) |
| Democratic People's Republic of Korea | 1 (0-2) | 0.01 (0-0.02) | 2 (0-4) | 0.01 (0-0.02) | 0.37 (0.06-0.68) |
| Democratic Republic of the Congo | 1 (0-2) | 0.02 (0-0.04) | 2 (0-5) | 0.02 (0-0.04) | -0.45 (-2-1.14) |
| Denmark | 0 (0-1) | 0.01 (0-0.01) | 0 (0-1) | 0 (0-0.01) | -2.98 (-4.22--1.73) |
| Djibouti | 0 (0-0) | 0.01 (0-0.02) | 0 (0-0) | 0.01 (0-0.02) | 0.08 (-1.77-1.96) |
| Dominica | 0 (0-0) | 0.01 (0-0.03) | 0 (0-0) | 0.01 (0-0.02) | -0.29 (-0.6-0.03) |
| Dominican Republic | 0 (0-0) | 0.01 (0-0.01) | 0 (0-0) | 0 (0-0) | -0.29 (-1.01-0.43) |
| Ecuador | 0 (0-0) | 0.01 (0-0.01) | 0 (0-0) | 0 (0-0.01) | -1.7 (-2.38--1.02) |
| Egypt | 4 (1-9) | 0.06 (0.01-0.12) | 7 (2-13) | 0.04 (0.01-0.09) | -0.15 (-1.8-1.53) |
| El Salvador | 0 (0-0) | 0.01 (0-0.01) | 0 (0-0) | 0 (0-0.01) | -0.61 (-1.06--0.15) |
| Equatorial Guinea | 0 (0-0) | 0.02 (0-0.04) | 0 (0-0) | 0.01 (0-0.03) | -1.41 (-3.05-0.25) |
| Eritrea | 0 (0-0) | 0.02 (0-0.04) | 0 (0-0) | 0.01 (0-0.03) | -0.14 (-1.92-1.68) |
| Estonia | 0 (0-1) | 0.03 (0.01-0.05) | 0 (0-0) | 0 (0-0.01) | -6.77 (-7.93--5.59) |
| Eswatini | 0 (0-0) | 0.02 (0-0.04) | 0 (0-0) | 0.02 (0-0.04) | 1.19 (-0.46-2.86) |
| Ethiopia | 1 (0-2) | 0.01 (0-0.02) | 1 (0-3) | 0.01 (0-0.02) | -1.31 (-3.13-0.54) |
| Fiji | 0 (0-0) | 0 (0-0.01) | 0 (0-0) | 0.01 (0-0.01) | 1.46 (0.31-2.62) |
| Finland | 1 (0-1) | 0.01 (0-0.02) | 0 (0-1) | 0 (0-0.01) | -2.68 (-4.02--1.32) |
| France | 4 (1-9) | 0.01 (0-0.01) | 3 (1-6) | 0 (0-0) | -3.11 (-4.51--1.69) |
| Gabon | 0 (0-0) | 0.02 (0-0.03) | 0 (0-0) | 0.01 (0-0.03) | -0.92 (-1.93-0.11) |
| Gambia | 0 (0-0) | 0.01 (0-0.03) | 0 (0-0) | 0.02 (0-0.04) | 1.24 (-0.35-2.85) |
| Georgia | 1 (0-1) | 0.02 (0-0.03) | 1 (0-2) | 0.02 (0-0.03) | 0.95 (-0.07-1.99) |
| Germany | 9 (2-19) | 0.01 (0-0.02) | 4 (1-8) | 0 (0-0) | -3.8 (-5.19--2.4) |
| Ghana | 0 (0-1) | 0.02 (0.01-0.04) | 1 (0-3) | 0.02 (0-0.04) | 0.24 (-1.19-1.69) |
| Greece | 2 (0-3) | 0.02 (0-0.04) | 1 (0-2) | 0.01 (0-0.01) | -3.35 (-4.43--2.26) |
| Greenland | 0 (0-0) | 0.01 (0-0.03) | 0 (0-0) | 0 (0-0.01) | -3.8 (-4.63--2.97) |
| Grenada | 0 (0-0) | 0.01 (0-0.03) | 0 (0-0) | 0.01 (0-0.01) | -2.51 (-2.85--2.16) |
| Guam | 0 (0-0) | 0.01 (0-0.02) | 0 (0-0) | 0 (0-0) | -1.89 (-2.65--1.12) |
| Guatemala | 0 (0-0) | 0.01 (0-0.01) | 0 (0-0) | 0 (0-0.01) | -1.92 (-3.31--0.51) |
| Guinea | 0 (0-1) | 0.02 (0-0.04) | 0 (0-1) | 0.02 (0-0.04) | 0.01 (-1.17-1.21) |
| Guinea-Bissau | 0 (0-0) | 0.02 (0-0.04) | 0 (0-0) | 0.02 (0-0.04) | 0.23 (-1.57-2.06) |
| Guyana | 0 (0-0) | 0.03 (0.01-0.05) | 0 (0-0) | 0.02 (0-0.03) | 0 (-0.93-0.93) |
| Haiti | 0 (0-1) | 0.03 (0.01-0.05) | 0 (0-1) | 0.02 (0-0.04) | -1.01 (-2.31-0.31) |
| Honduras | 0 (0-0) | 0.01 (0-0.02) | 0 (0-1) | 0.01 (0-0.03) | 1.18 (-0.04-2.41) |
| Hungary | 2 (1-4) | 0.02 (0.01-0.05) | 1 (0-2) | 0.01 (0-0.01) | -3.69 (-4.69--2.67) |
| Iceland | 0 (0-0) | 0.01 (0-0.01) | 0 (0-0) | 0 (0-0) | -2.84 (-3.71--1.97) |
| India | 17 (5-37) | 0.01 (0-0.02) | 39 (11-79) | 0.01 (0-0.01) | -0.18 (-0.99-0.65) |
| Indonesia | 7 (2-15) | 0.02 (0.01-0.04) | 19 (5-38) | 0.02 (0.01-0.04) | 1.16 (0.23-2.1) |
| Iran (Islamic Republic of) | 1 (0-3) | 0.02 (0-0.03) | 3 (1-5) | 0.01 (0-0.02) | -1.01 (-2.04-0.04) |
| Iraq | 1 (0-2) | 0.03 (0.01-0.05) | 2 (1-4) | 0.02 (0.01-0.04) | -1.38 (-2.5--0.24) |
| Ireland | 0 (0-1) | 0.01 (0-0.02) | 0 (0-0) | 0 (0-0) | -4.32 (-4.98--3.65) |
| Israel | 0 (0-0) | 0 (0-0.01) | 0 (0-0) | 0 (0-0) | -3.65 (-4.11--3.2) |
| Italy | 5 (1-11) | 0.01 (0-0.02) | 4 (1-8) | 0 (0-0.01) | -2.99 (-4.5--1.46) |
| Jamaica | 0 (0-0) | 0.01 (0-0.02) | 0 (0-0) | 0.01 (0-0.02) | -0.73 (-1.12--0.35) |
| Japan | 8 (2-16) | 0.01 (0-0.02) | 7 (1-16) | 0 (0-0) | -2.92 (-4.57--1.24) |
| Jordan | 0 (0-0) | 0.03 (0.01-0.05) | 0 (0-0) | 0.01 (0-0.02) | -3.34 (-5.01--1.63) |
| Kazakhstan | 2 (0-3) | 0.02 (0.01-0.05) | 1 (0-3) | 0.02 (0-0.03) | -1.84 (-2.22--1.46) |
| Kenya | 0 (0-1) | 0.01 (0-0.02) | 1 (0-2) | 0.01 (0-0.02) | 0.81 (-0.79-2.44) |
| Kiribati | 0 (0-0) | 0.01 (0-0.02) | 0 (0-0) | 0.01 (0-0.02) | 0.26 (-0.95-1.49) |
| Kuwait | 0 (0-0) | 0.01 (0-0.01) | 0 (0-0) | 0 (0-0) | -2.74 (-4.27--1.2) |
| Kyrgyzstan | 0 (0-1) | 0.02 (0.01-0.05) | 0 (0-1) | 0.01 (0-0.02) | -3.3 (-3.84--2.76) |
| Lao People's Democratic Republic | 0 (0-0) | 0.03 (0.01-0.06) | 0 (0-1) | 0.02 (0-0.04) | -1.4 (-2.55--0.24) |
| Latvia | 1 (0-1) | 0.03 (0.01-0.05) | 0 (0-1) | 0.01 (0-0.03) | -1.88 (-3.07--0.68) |
| Lebanon | 0 (0-0) | 0.01 (0-0.02) | 0 (0-0) | 0 (0-0.01) | -2.05 (-2.46--1.64) |
| Lesotho | 0 (0-0) | 0.01 (0-0.03) | 0 (0-0) | 0.02 (0.01-0.04) | 2.68 (1.77-3.6) |
| Liberia | 0 (0-0) | 0.02 (0-0.04) | 0 (0-0) | 0.02 (0-0.04) | -0.74 (-2.27-0.81) |
| Libya | 0 (0-0) | 0.01 (0-0.02) | 0 (0-0) | 0.01 (0-0.02) | 1.29 (0.24-2.36) |
| Lithuania | 0 (0-1) | 0.01 (0-0.03) | 0 (0-1) | 0.01 (0-0.02) | -1.1 (-2.19-0.01) |
| Luxembourg | 0 (0-0) | 0.01 (0-0.03) | 0 (0-0) | 0 (0-0) | -5.26 (-6.26--4.24) |
| Madagascar | 0 (0-1) | 0.02 (0.01-0.04) | 1 (0-1) | 0.02 (0-0.04) | -0.8 (-2.54-0.96) |
| Malawi | 0 (0-0) | 0.02 (0-0.03) | 0 (0-1) | 0.02 (0-0.03) | -0.35 (-1.94-1.27) |
| Malaysia | 0 (0-1) | 0.01 (0-0.02) | 1 (0-2) | 0.01 (0-0.02) | 0.27 (-0.68-1.23) |
| Maldives | 0 (0-0) | 0.02 (0.01-0.04) | 0 (0-0) | 0.01 (0-0.02) | -1.91 (-3.39--0.42) |
| Mali | 0 (0-1) | 0.02 (0-0.04) | 0 (0-1) | 0.01 (0-0.03) | -0.72 (-2.42-1.02) |
| Malta | 0 (0-0) | 0.01 (0-0.01) | 0 (0-0) | 0 (0-0) | -2.93 (-3.9--1.94) |
| Marshall Islands | 0 (0-0) | 0.01 (0-0.03) | 0 (0-0) | 0.01 (0-0.03) | 0.01 (-1.71-1.77) |
| Mauritania | 0 (0-0) | 0.02 (0-0.04) | 0 (0-0) | 0.01 (0-0.03) | -1.05 (-2.33-0.25) |
| Mauritius | 0 (0-0) | 0.01 (0-0.01) | 0 (0-0) | 0 (0-0) | -2.96 (-3.45--2.47) |
| Mexico | 1 (0-3) | 0.01 (0-0.02) | 2 (0-4) | 0 (0-0.01) | -1.76 (-2.59--0.92) |
| Micronesia (Federated States of) | 0 (0-0) | 0.02 (0-0.03) | 0 (0-0) | 0.01 (0-0.03) | -0.3 (-1.4-0.82) |
| Monaco | 0 (0-0) | 0.01 (0-0.02) | 0 (0-0) | 0 (0-0.01) | -3.2 (-5.01--1.37) |
| Mongolia | 0 (0-0) | 0.01 (0-0.01) | 0 (0-0) | 0 (0-0.01) | -0.89 (-1.94-0.16) |
| Montenegro | 0 (0-0) | 0.01 (0-0.01) | 0 (0-0) | 0.01 (0-0.02) | 3.02 (2.5-3.54) |
| Morocco | 1 (0-3) | 0.02 (0.01-0.05) | 3 (1-6) | 0.02 (0-0.04) | 0.37 (-0.32-1.06) |
| Mozambique | 0 (0-1) | 0.02 (0-0.03) | 1 (0-1) | 0.02 (0-0.03) | -0.04 (-1.6-1.54) |
| Myanmar | 2 (1-4) | 0.02 (0.01-0.05) | 3 (1-7) | 0.01 (0-0.03) | -1.14 (-1.84--0.44) |
| Namibia | 0 (0-0) | 0.02 (0.01-0.04) | 0 (0-0) | 0.02 (0-0.03) | -0.51 (-1.77-0.76) |
| Nauru | 0 (0-0) | 0.02 (0-0.04) | 0 (0-0) | 0.02 (0-0.04) | 0.47 (-0.98-1.93) |
| Nepal | 1 (0-1) | 0.01 (0-0.03) | 1 (0-2) | 0.01 (0-0.02) | -1.61 (-2.7--0.52) |
| Netherlands | 1 (0-2) | 0.01 (0-0.01) | 1 (0-1) | 0 (0-0.01) | -2.28 (-3.41--1.13) |
| New Zealand | 0 (0-0) | 0.01 (0-0.02) | 0 (0-0) | 0 (0-0.01) | -2.25 (-3.06--1.44) |
| Nicaragua | 0 (0-0) | 0 (0-0.01) | 0 (0-0) | 0 (0-0.01) | -0.65 (-1.55-0.25) |
| Niger | 0 (0-0) | 0.01 (0-0.03) | 0 (0-1) | 0.01 (0-0.03) | -0.08 (-2.13-2) |
| Nigeria | 3 (1-7) | 0.02 (0-0.04) | 4 (1-8) | 0.01 (0-0.02) | -1.85 (-3.38--0.29) |
| Niue | 0 (0-0) | 0.01 (0-0.02) | 0 (0-0) | 0.01 (0-0.02) | -0.92 (-1.39--0.45) |
| North Macedonia | 0 (0-1) | 0.04 (0.01-0.08) | 1 (0-1) | 0.04 (0.01-0.08) | 0.4 (0.03-0.77) |
| Northern Mariana Islands | 0 (0-0) | 0.01 (0-0.02) | 0 (0-0) | 0.01 (0-0.01) | 0.42 (-1.29-2.16) |
| Norway | 0 (0-1) | 0 (0-0.01) | 0 (0-0) | 0 (0-0) | -4.27 (-5.6--2.93) |
| Oman | 0 (0-0) | 0.02 (0-0.03) | 0 (0-0) | 0.01 (0-0.02) | -0.91 (-2.34-0.53) |
| Pakistan | 3 (1-6) | 0.01 (0-0.03) | 6 (2-11) | 0.01 (0-0.03) | -0.7 (-2.02-0.64) |
| Palau | 0 (0-0) | 0.01 (0-0.03) | 0 (0-0) | 0.01 (0-0.03) | 1.04 (0.27-1.82) |
| Palestine | 0 (0-0) | 0.03 (0.01-0.07) | 0 (0-0) | 0.02 (0-0.04) | -2.05 (-3.47--0.62) |
| Panama | 0 (0-0) | 0 (0-0.01) | 0 (0-0) | 0 (0-0) | -1.13 (-1.54--0.71) |
| Papua New Guinea | 0 (0-0) | 0.01 (0-0.03) | 0 (0-0) | 0.01 (0-0.03) | -0.51 (-2.18-1.19) |
| Paraguay | 0 (0-0) | 0.01 (0-0.02) | 0 (0-0) | 0 (0-0.01) | -2.09 (-2.67--1.51) |
| Peru | 0 (0-1) | 0 (0-0.01) | 0 (0-1) | 0 (0-0.01) | -1.63 (-2.23--1.02) |
| Philippines | 1 (0-3) | 0.01 (0-0.03) | 4 (1-7) | 0.01 (0-0.02) | 0.37 (-0.77-1.52) |
| Poland | 6 (2-13) | 0.02 (0.01-0.05) | 4 (1-8) | 0.01 (0-0.02) | -3.02 (-3.85--2.17) |
| Portugal | 2 (0-4) | 0.02 (0.01-0.05) | 1 (0-2) | 0 (0-0.01) | -4.73 (-5.89--3.57) |
| Puerto Rico | 0 (0-0) | 0 (0-0.01) | 0 (0-0) | 0 (0-0) | -3.05 (-3.94--2.14) |
| Qatar | 0 (0-0) | 0.01 (0-0.03) | 0 (0-0) | 0 (0-0.01) | -3.88 (-6.4--1.29) |
| Republic of Korea | 2 (1-5) | 0.02 (0-0.04) | 1 (0-3) | 0 (0-0.01) | -5.11 (-5.69--4.51) |
| Republic of Moldova | 0 (0-1) | 0.02 (0-0.04) | 0 (0-1) | 0.01 (0-0.02) | -0.67 (-1.31--0.02) |
| Romania | 4 (1-8) | 0.03 (0.01-0.06) | 4 (1-8) | 0.01 (0-0.03) | -1.17 (-1.98--0.35) |
| Russian Federation | 41 (11-80) | 0.04 (0.01-0.07) | 30 (7-61) | 0.02 (0-0.04) | -2.56 (-3.36--1.75) |
| Rwanda | 0 (0-1) | 0.03 (0.01-0.06) | 0 (0-1) | 0.01 (0-0.03) | -3.17 (-4.73--1.58) |
| Saint Kitts and Nevis | 0 (0-0) | 0.02 (0.01-0.05) | 0 (0-0) | 0.01 (0-0.02) | -2.45 (-2.61--2.28) |
| Saint Lucia | 0 (0-0) | 0.03 (0.01-0.05) | 0 (0-0) | 0.01 (0-0.02) | -2.41 (-2.98--1.84) |
| Saint Vincent and the Grenadines | 0 (0-0) | 0.02 (0-0.04) | 0 (0-0) | 0.01 (0-0.02) | -1.21 (-1.56--0.85) |
| Samoa | 0 (0-0) | 0.01 (0-0.02) | 0 (0-0) | 0.01 (0-0.02) | -0.04 (-0.92-0.84) |
| San Marino | 0 (0-0) | 0.01 (0-0.01) | 0 (0-0) | 0 (0-0.01) | -2.62 (-4.09--1.12) |
| Sao Tome and Principe | 0 (0-0) | 0.01 (0-0.03) | 0 (0-0) | 0.02 (0-0.03) | -0.02 (-1.1-1.07) |
| Saudi Arabia | 0 (0-1) | 0.02 (0.01-0.05) | 1 (0-2) | 0.02 (0-0.03) | -1.32 (-2.86-0.24) |
| Senegal | 0 (0-0) | 0.01 (0-0.03) | 0 (0-1) | 0.01 (0-0.03) | -0.09 (-1.5-1.34) |
| Serbia | 2 (1-4) | 0.04 (0.01-0.09) | 2 (1-5) | 0.02 (0.01-0.05) | -1.23 (-1.93--0.52) |
| Seychelles | 0 (0-0) | 0.01 (0-0.02) | 0 (0-0) | 0.01 (0-0.02) | 0.08 (-0.1-0.25) |
| Sierra Leone | 0 (0-0) | 0.02 (0-0.04) | 0 (0-1) | 0.02 (0-0.04) | 0.05 (-1.3-1.41) |
| Singapore | 0 (0-0) | 0.01 (0-0.02) | 0 (0-0) | 0 (0-0) | -5.81 (-6.24--5.38) |
| Slovakia | 1 (0-1) | 0.02 (0-0.04) | 0 (0-1) | 0.01 (0-0.02) | -2.11 (-2.83--1.38) |
| Slovenia | 0 (0-0) | 0.01 (0-0.03) | 0 (0-0) | 0 (0-0.01) | -3.22 (-4.39--2.03) |
| Solomon Islands | 0 (0-0) | 0.02 (0-0.04) | 0 (0-0) | 0.02 (0-0.03) | 0.47 (-1.34-2.31) |
| Somalia | 0 (0-0) | 0.01 (0-0.03) | 0 (0-1) | 0.01 (0-0.03) | -0.78 (-2.84-1.32) |
| South Africa | 1 (0-2) | 0.01 (0-0.02) | 2 (1-4) | 0.01 (0-0.02) | 0.92 (0.23-1.61) |
| South Sudan | 0 (0-0) | 0.01 (0-0.03) | 0 (0-0) | 0.01 (0-0.02) | -1.17 (-3.03-0.72) |
| Spain | 4 (1-8) | 0.01 (0-0.02) | 2 (0-4) | 0 (0-0) | -4.31 (-5.63--2.97) |
| Sri Lanka | 1 (0-2) | 0.02 (0.01-0.05) | 2 (0-4) | 0.01 (0-0.03) | 0.93 (0.28-1.59) |
| Sudan | 1 (0-2) | 0.03 (0.01-0.06) | 2 (0-3) | 0.02 (0-0.05) | -1.51 (-2.85--0.15) |
| Suriname | 0 (0-0) | 0.01 (0-0.02) | 0 (0-0) | 0 (0-0.01) | -2.05 (-2.53--1.57) |
| Sweden | 1 (0-2) | 0.01 (0-0.02) | 0 (0-1) | 0 (0-0.01) | -3.14 (-4.65--1.61) |
| Switzerland | 1 (0-1) | 0.01 (0-0.01) | 0 (0-1) | 0 (0-0) | -3.54 (-4.95--2.11) |
| Syrian Arab Republic | 0 (0-1) | 0.02 (0.01-0.04) | 1 (0-1) | 0.02 (0-0.03) | -0.64 (-2.3-1.05) |
| Taiwan (Province of China) | 0 (0-0) | 0 (0-0.01) | 0 (0-0) | 0 (0-0) | -4.1 (-4.68--3.52) |
| Tajikistan | 0 (0-1) | 0.02 (0.01-0.04) | 0 (0-1) | 0.02 (0-0.03) | -1.29 (-2.41--0.17) |
| Thailand | 1 (0-3) | 0.01 (0-0.02) | 3 (1-6) | 0 (0-0.01) | -0.85 (-1.38--0.31) |
| Timor-Leste | 0 (0-0) | 0.02 (0-0.03) | 0 (0-0) | 0.02 (0-0.03) | 1.35 (-0.16-2.88) |
| Togo | 0 (0-0) | 0.02 (0-0.04) | 0 (0-1) | 0.02 (0-0.04) | 0.32 (-1.39-2.05) |
| Tokelau | 0 (0-0) | 0.01 (0-0.03) | 0 (0-0) | 0.01 (0-0.02) | -0.6 (-0.85--0.36) |
| Tonga | 0 (0-0) | 0.01 (0-0.01) | 0 (0-0) | 0.01 (0-0.01) | 1.24 (0.61-1.87) |
| Trinidad and Tobago | 0 (0-0) | 0.01 (0-0.02) | 0 (0-0) | 0 (0-0.01) | -2.46 (-2.86--2.07) |
| Tunisia | 0 (0-0) | 0.01 (0-0.03) | 1 (0-1) | 0.01 (0-0.02) | 0.24 (-0.37-0.85) |
| Turkey | 3 (1-5) | 0.02 (0-0.03) | 4 (1-9) | 0.01 (0-0.02) | -0.36 (-0.87-0.14) |
| Turkmenistan | 0 (0-0) | 0.02 (0.01-0.04) | 0 (0-1) | 0.02 (0.01-0.04) | 0.51 (-0.22-1.24) |
| Tuvalu | 0 (0-0) | 0.02 (0-0.04) | 0 (0-0) | 0.01 (0-0.03) | -0.46 (-0.98-0.06) |
| Uganda | 0 (0-1) | 0.01 (0-0.03) | 1 (0-1) | 0.01 (0-0.02) | -1.98 (-3.74--0.19) |
| Ukraine | 14 (3-27) | 0.03 (0.01-0.06) | 8 (2-16) | 0.01 (0-0.03) | -2.6 (-3.45--1.75) |
| United Arab Emirates | 0 (0-0) | 0.01 (0-0.03) | 0 (0-0) | 0.03 (0.01-0.06) | 2.15 (-0.96-5.36) |
| United Kingdom | 6 (1-13) | 0.01 (0-0.02) | 2 (0-5) | 0 (0-0) | -4.39 (-5.64--3.13) |
| United Republic of Tanzania | 0 (0-1) | 0.01 (0-0.02) | 1 (0-2) | 0.01 (0-0.02) | 0.54 (-0.95-2.06) |
| United States of America | 6 (1-12) | 0 (0-0.01) | 5 (1-10) | 0 (0-0) | -2.52 (-3.52--1.51) |
| United States Virgin Islands | 0 (0-0) | 0.01 (0-0.01) | 0 (0-0) | 0 (0-0) | -1.96 (-2.53--1.38) |
| Uruguay | 0 (0-1) | 0.01 (0-0.03) | 0 (0-0) | 0 (0-0.01) | -3 (-3.93--2.06) |
| Uzbekistan | 1 (0-2) | 0.01 (0-0.03) | 1 (0-3) | 0.01 (0-0.02) | -1.05 (-1.76--0.33) |
| Vanuatu | 0 (0-0) | 0.01 (0-0.03) | 0 (0-0) | 0.01 (0-0.02) | -0.08 (-1.71-1.58) |
| Venezuela (Bolivarian Republic of) | 0 (0-0) | 0 (0-0.01) | 1 (0-1) | 0 (0-0.01) | 0.13 (-0.55-0.81) |
| Viet Nam | 4 (1-9) | 0.02 (0-0.04) | 8 (2-17) | 0.02 (0-0.03) | 0.67 (0.23-1.11) |
| Yemen | 1 (0-1) | 0.03 (0.01-0.07) | 2 (0-3) | 0.03 (0.01-0.06) | -0.59 (-2.12-0.96) |
| Zambia | 0 (0-0) | 0.02 (0-0.03) | 0 (0-1) | 0.02 (0-0.03) | 0.05 (-1.87-2.02) |

Abbreviations: IS,Ischemic stroke,IIPUFAs, insufficient intake of polyunsaturated fatty acids,EAPC, estimated annual percentage change, SDl, Sociodemographic Index; Ul,uncertainty interval. “ EAPC is expressed as 95% CIs.

Table 3 The YLDs cases and age-standardized YLDs rate of ischemic stroke attributed to IIPUFAs disease in 1990 and 2021, along with their temporal trend.

|  | Rate per 100 000(95%UI) | | 2021 | | 1990-2021 |
| --- | --- | --- | --- | --- | --- |
|  | 1990 | |  | |  |
|  | YLDs cases | The age-standardized YLDs rate | YLDs cases | The age-standardized YLDs rate | EAPC |

| **Global** | 894 (283-1674) | 0.04 (0.01-0.08) | 1631 (490-3069) | 0.04 (0.01-0.07) | 0.07 (-0.07-0.22) |
| --- | --- | --- | --- | --- | --- |
| **Age** |  |  |  |  |  |
| 50-54 years | 87 (29-165) | 0.08 (0.03-0.16) | 163 (54-307) | 0.07 (0.02-0.14) | -0.41 (-0.47--0.35) |
| 55-59 years | 98 (31-190) | 0.11 (0.03-0.21) | 189 (59-369) | 0.09 (0.03-0.18) | -0.49 (-0.57--0.41) |
| 60-64 years | 106 (30-204) | 0.13 (0.04-0.25) | 181 (51-351) | 0.11 (0.03-0.21) | -0.57 (-0.67--0.46) |
| 65-69 years | 96 (26-191) | 0.15 (0.04-0.29) | 190 (51-376) | 0.13 (0.04-0.26) | -0.55 (-0.65--0.44) |
| 70-74 years | 75 (19-155) | 0.16 (0.04-0.33) | 158 (41-326) | 0.14 (0.04-0.3) | -0.47 (-0.53--0.42) |
| 75-79 years | 62 (15-130) | 0.17 (0.04-0.36) | 118 (28-245) | 0.16 (0.04-0.34) | -0.22 (-0.3--0.15) |
| 80-84 years | 40 (9-88) | 0.18 (0.04-0.4) | 95 (21-206) | 0.19 (0.04-0.4) | 0.03 (-0.08-0.14) |
| 85-89 years | 18 (4-40) | 0.18 (0.04-0.4) | 59 (12-129) | 0.21 (0.04-0.45) | 0.3 (0.2-0.41) |
| 90-94 years | 6 (1-13) | 0.19 (0.04-0.43) | 26 (6-58) | 0.22 (0.05-0.48) | 0.28 (0.19-0.37) |
| 95+ years | 1 (0-3) | 0.2 (0.04-0.45) | 9 (2-20) | 0.23 (0.05-0.51) | 0.33 (0.24-0.42) |
| **SDI region** |  |  |  |  |  |
| High SDI | 157 (45-298) | 0.03 (0.01-0.05) | 194 (54-381) | 0.02 (0.01-0.04) | -1.1 (-1.16--1.04) |
| High-middle SDI | 273 (86-517) | 0.05 (0.02-0.09) | 426 (125-807) | 0.04 (0.01-0.08) | -0.58 (-0.64--0.51) |
| Middle SDI | 269 (87-498) | 0.04 (0.01-0.08) | 599 (182-1136) | 0.04 (0.01-0.08) | -0.25 (-0.31--0.19) |
| Low-middle SDI | 131 (43-241) | 0.04 (0.01-0.07) | 279 (91-518) | 0.03 (0.01-0.06) | -0.36 (-0.39--0.32) |
| Low SDI | 63 (21-117) | 0.05 (0.02-0.09) | 131 (43-247) | 0.04 (0.01-0.08) | -0.54 (-0.58--0.49) |
| **GBD region** |  |  |  |  |  |
| Advanced Health System | 289 (85-549) | 0.03 (0.01-0.06) | 309 (87-595) | 0.02 (0.01-0.04) | -0.83 (-1.38--0.27) |
| Africa | 94 (31-175) | 0.06 (0.02-0.11) | 203 (66-381) | 0.05 (0.02-0.09) | -0.31 (-1.16-0.54) |
| African Region | 79 (25-146) | 0.06 (0.02-0.12) | 169 (55-318) | 0.05 (0.02-0.1) | -0.42 (-1.35-0.51) |
| America | 61 (18-116) | 0.02 (0.01-0.03) | 84 (24-162) | 0.01 (0-0.02) | -0.98 (-1.17--0.78) |
| Andean Latin America | 2 (1-4) | 0.02 (0.01-0.03) | 4 (1-8) | 0.01 (0-0.02) | -0.2 (-0.58-0.19) |
| Asia | 523 (170-969) | 0.05 (0.01-0.08) | 1126 (339-2136) | 0.04 (0.01-0.08) | 0.47 (0.24-0.69) |
| Australasia | 3 (1-6) | 0.02 (0.01-0.05) | 5 (1-9) | 0.02 (0.01-0.03) | -0.73 (-1.23--0.22) |
| Basic Health System | 417 (135-774) | 0.05 (0.02-0.09) | 910 (270-1743) | 0.05 (0.01-0.09) | 0.63 (0.41-0.85) |
| Caribbean | 2 (1-4) | 0.02 (0.01-0.03) | 3 (1-6) | 0.01 (0-0.02) | -0.38 (-0.55--0.21) |
| Central Africa | 11 (4-21) | 0.07 (0.02-0.13) | 25 (8-46) | 0.06 (0.02-0.11) | -0.44 (-1.4-0.54) |
| Central Asia | 22 (7-40) | 0.08 (0.02-0.14) | 31 (10-57) | 0.06 (0.02-0.12) | -0.34 (-0.55--0.13) |
| Central Europe | 37 (11-69) | 0.05 (0.01-0.09) | 36 (10-70) | 0.03 (0.01-0.06) | -0.5 (-1.09-0.08) |
| Central Latin America | 9 (3-18) | 0.02 (0.01-0.04) | 18 (6-34) | 0.01 (0-0.02) | -0.42 (-0.82--0.01) |
| Central Sub-Saharan Africa | 8 (3-16) | 0.06 (0.02-0.12) | 19 (6-36) | 0.06 (0.02-0.1) | -0.44 (-1.42-0.55) |
| Commonwealth High Income | 21 (6-40) | 0.03 (0.01-0.05) | 26 (7-51) | 0.02 (0.01-0.04) | -0.6 (-1.2-0.01) |
| Commonwealth Low Income | 22 (7-41) | 0.05 (0.01-0.09) | 53 (17-99) | 0.04 (0.01-0.07) | 0.14 (-0.75-1.03) |
| Commonwealth Middle Income | 123 (38-226) | 0.03 (0.01-0.06) | 266 (87-491) | 0.03 (0.01-0.05) | -0.05 (-0.5-0.41) |
| East Asia | 253 (82-470) | 0.05 (0.02-0.1) | 586 (169-1121) | 0.05 (0.02-0.1) | 1.06 (0.77-1.35) |
| East Asia & Pacific - WB | 394 (127-734) | 0.05 (0.02-0.1) | 851 (249-1631) | 0.05 (0.02-0.1) | 0.78 (0.53-1.03) |
| Eastern Africa | 24 (8-44) | 0.06 (0.02-0.11) | 50 (16-94) | 0.05 (0.02-0.09) | -0.34 (-1.38-0.72) |
| Eastern Europe | 95 (30-184) | 0.06 (0.02-0.11) | 88 (26-168) | 0.05 (0.02-0.09) | -0.44 (-0.99-0.11) |
| Eastern Mediterranean Region | 41 (14-75) | 0.04 (0.01-0.07) | 98 (33-184) | 0.04 (0.01-0.07) | 0.08 (-0.51-0.67) |
| Eastern Sub-Saharan Africa | 27 (9-50) | 0.06 (0.02-0.12) | 55 (18-104) | 0.05 (0.02-0.1) | -0.44 (-1.53-0.66) |
| Europe | 215 (64-407) | 0.04 (0.01-0.07) | 215 (61-405) | 0.03 (0.01-0.05) | -0.66 (-1.23--0.08) |
| Europe & Central Asia - WB | 231 (69-436) | 0.04 (0.01-0.07) | 238 (69-448) | 0.03 (0.01-0.06) | -0.59 (-1.12--0.07) |
| European Region | 232 (69-438) | 0.04 (0.01-0.07) | 240 (69-451) | 0.03 (0.01-0.06) | -0.6 (-1.12--0.08) |
| High-income Asia Pacific | 37 (11-70) | 0.03 (0.01-0.06) | 43 (12-88) | 0.02 (0.01-0.04) | -0.8 (-1.58--0.02) |
| High-income North America | 36 (10-69) | 0.02 (0.01-0.04) | 42 (12-83) | 0.01 (0-0.03) | -1.17 (-1.67--0.66) |
| Latin America & Caribbean - WB | 25 (8-48) | 0.02 (0.01-0.03) | 43 (13-82) | 0.01 (0-0.02) | -0.57 (-0.85--0.29) |
| Limited Health System | 166 (53-305) | 0.04 (0.01-0.07) | 366 (120-680) | 0.03 (0.01-0.06) | -0.02 (-0.56-0.52) |
| Middle East & North Africa - WB | 27 (9-51) | 0.04 (0.01-0.07) | 64 (21-119) | 0.03 (0.01-0.06) | 0.1 (-0.38-0.58) |
| Minimal Health System | 21 (7-40) | 0.06 (0.02-0.11) | 44 (15-82) | 0.06 (0.02-0.1) | -0.49 (-1.47-0.5) |
| North Africa and Middle East | 41 (14-76) | 0.04 (0.01-0.07) | 87 (29-164) | 0.03 (0.01-0.06) | -0.13 (-0.59-0.34) |
| North America | 36 (10-69) | 0.02 (0.01-0.04) | 42 (12-83) | 0.01 (0-0.03) | -1.17 (-1.67--0.66) |
| Northern Africa | 15 (5-29) | 0.04 (0.01-0.08) | 33 (11-62) | 0.04 (0.01-0.07) | 0.22 (-0.21-0.65) |
| Oceania | 1 (0-2) | 0.05 (0.01-0.09) | 2 (1-4) | 0.04 (0.01-0.08) | 0.18 (-0.53-0.89) |
| Region of the Americas | 61 (18-116) | 0.02 (0.01-0.03) | 84 (24-162) | 0.01 (0-0.02) | -0.98 (-1.17--0.78) |
| South-East Asia Region | 165 (53-306) | 0.04 (0.01-0.07) | 343 (110-639) | 0.03 (0.01-0.06) | 0.09 (-0.27-0.46) |
| South Asia | 96 (31-177) | 0.03 (0.01-0.05) | 210 (69-390) | 0.03 (0.01-0.05) | 0.14 (-0.28-0.56) |
| South Asia - WB | 101 (32-186) | 0.03 (0.01-0.05) | 221 (72-409) | 0.03 (0.01-0.05) | 0.13 (-0.29-0.55) |
| Southeast Asia | 104 (34-192) | 0.07 (0.02-0.12) | 222 (70-421) | 0.06 (0.02-0.11) | 0.4 (0.09-0.72) |
| Southern Africa | 15 (5-29) | 0.06 (0.02-0.11) | 27 (8-51) | 0.05 (0.01-0.09) | -0.83 (-1.56--0.09) |
| Southern Latin America | 7 (2-14) | 0.03 (0.01-0.05) | 9 (3-18) | 0.02 (0.01-0.04) | -0.85 (-1.07--0.63) |
| Southern Sub-Saharan Africa | 10 (3-18) | 0.06 (0.02-0.11) | 14 (4-27) | 0.04 (0.01-0.08) | -0.97 (-1.45--0.5) |
| Sub-Saharan Africa - WB | 79 (26-147) | 0.06 (0.02-0.12) | 170 (55-319) | 0.05 (0.02-0.1) | -0.42 (-1.36-0.54) |
| Tropical Latin America | 5 (2-9) | 0.01 (0-0.02) | 9 (3-17) | 0.01 (0-0.01) | -0.31 (-0.59--0.04) |
| Western Africa | 28 (9-52) | 0.06 (0.02-0.12) | 68 (22-124) | 0.06 (0.02-0.11) | -0.28 (-1.24-0.68) |
| Western Europe | 69 (19-134) | 0.02 (0.01-0.04) | 72 (20-142) | 0.02 (0-0.03) | -0.73 (-1.53-0.08) |
| Western Pacific Region | 314 (101-587) | 0.05 (0.02-0.09) | 693 (200-1322) | 0.05 (0.01-0.09) | 0.88 (0.6-1.16) |
| Western Sub-Saharan Africa | 31 (10-58) | 0.06 (0.02-0.12) | 76 (25-140) | 0.06 (0.02-0.11) | -0.3 (-1.25-0.67) |
| World Bank High Income | 180 (51-341) | 0.03 (0.01-0.05) | 203 (57-401) | 0.02 (0.01-0.03) | -0.84 (-1.47--0.22) |
| World Bank Low Income | 47 (15-87) | 0.05 (0.02-0.1) | 93 (31-175) | 0.05 (0.01-0.09) | -0.46 (-1.34-0.43) |
| World Bank Lower Middle Income | 272 (89-503) | 0.05 (0.01-0.08) | 567 (183-1058) | 0.04 (0.01-0.07) | 0.01 (-0.43-0.46) |
| World Bank Upper Middle Income | 394 (127-734) | 0.05 (0.01-0.09) | 766 (224-1461) | 0.04 (0.01-0.08) | 0.46 (0.24-0.69) |
| **Countries** |  |  |  |  |  |
| Afghanistan | 2 (1-3) | 0.04 (0.01-0.08) | 3 (1-6) | 0.04 (0.01-0.07) | -0.82 (-1.62-0) |
| Albania | 0 (0-1) | 0.04 (0.01-0.07) | 1 (0-1) | 0.03 (0.01-0.06) | 0.77 (0.45-1.08) |
| Algeria | 3 (1-6) | 0.04 (0.01-0.08) | 7 (2-13) | 0.04 (0.01-0.07) | 0.04 (-0.43-0.51) |
| American Samoa | 0 (0-0) | 0.05 (0.02-0.1) | 0 (0-0) | 0.05 (0.02-0.1) | 0.66 (0.17-1.16) |
| Andorra | 0 (0-0) | 0.01 (0-0.03) | 0 (0-0) | 0.01 (0-0.02) | -0.04 (-0.54-0.46) |
| Angola | 1 (0-2) | 0.05 (0.02-0.1) | 4 (1-7) | 0.05 (0.01-0.09) | -0.5 (-1.63-0.65) |
| Antigua and Barbuda | 0 (0-0) | 0.01 (0-0.02) | 0 (0-0) | 0.01 (0-0.02) | 1.06 (0.89-1.23) |
| Argentina | 5 (1-9) | 0.03 (0.01-0.05) | 6 (2-12) | 0.02 (0.01-0.04) | -0.81 (-1.01--0.6) |
| Armenia | 1 (0-2) | 0.07 (0.02-0.13) | 1 (0-2) | 0.05 (0.02-0.09) | -0.73 (-1.02--0.43) |
| Australia | 3 (1-5) | 0.02 (0.01-0.05) | 4 (1-7) | 0.02 (0.01-0.03) | -0.77 (-1.29--0.25) |
| Austria | 1 (0-3) | 0.02 (0.01-0.04) | 2 (1-5) | 0.03 (0.01-0.05) | 1.09 (0.2-1.98) |
| Azerbaijan | 2 (1-3) | 0.06 (0.02-0.11) | 3 (1-6) | 0.05 (0.02-0.1) | 0.23 (0.04-0.41) |
| Bahamas | 0 (0-0) | 0.01 (0-0.02) | 0 (0-0) | 0.01 (0-0.02) | 1.05 (0.84-1.26) |
| Bahrain | 0 (0-0) | 0.02 (0.01-0.04) | 0 (0-0) | 0.02 (0.01-0.03) | -0.36 (-0.76-0.04) |
| Bangladesh | 8 (3-15) | 0.03 (0.01-0.06) | 22 (7-42) | 0.03 (0.01-0.06) | 0.95 (0.23-1.68) |
| Barbados | 0 (0-0) | 0.01 (0.01-0.03) | 0 (0-0) | 0.01 (0-0.03) | 0.41 (-0.02-0.83) |
| Belarus | 5 (1-9) | 0.06 (0.02-0.12) | 4 (1-8) | 0.05 (0.02-0.09) | -0.64 (-1.23--0.05) |
| Belgium | 1 (0-3) | 0.02 (0-0.03) | 1 (0-2) | 0.01 (0-0.02) | -0.57 (-1.35-0.21) |
| Belize | 0 (0-0) | 0.01 (0-0.03) | 0 (0-0) | 0.01 (0-0.02) | -0.69 (-1.31--0.06) |
| Benin | 1 (0-2) | 0.07 (0.02-0.13) | 2 (1-4) | 0.06 (0.02-0.11) | -0.48 (-1.49-0.54) |
| Bermuda | 0 (0-0) | 0.01 (0-0.03) | 0 (0-0) | 0.01 (0-0.02) | -0.05 (-0.59-0.5) |
| Bhutan | 0 (0-0) | 0.03 (0.01-0.05) | 0 (0-0) | 0.02 (0.01-0.04) | 0.25 (-0.35-0.86) |
| Bolivia (Plurinational State of) | 0 (0-0) | 0.01 (0-0.03) | 1 (0-1) | 0.01 (0-0.02) | -0.3 (-0.79-0.18) |
| Bosnia and Herzegovina | 2 (1-3) | 0.06 (0.02-0.12) | 2 (0-3) | 0.06 (0.02-0.1) | 0.22 (-0.28-0.72) |
| Botswana | 0 (0-0) | 0.06 (0.02-0.12) | 1 (0-1) | 0.06 (0.02-0.12) | 0.74 (-0.01-1.51) |
| Brazil | 5 (1-9) | 0.01 (0-0.02) | 9 (3-17) | 0.01 (0-0.01) | -0.26 (-0.53-0.01) |
| Brunei Darussalam | 0 (0-0) | 0.06 (0.02-0.12) | 0 (0-0) | 0.04 (0.01-0.07) | -0.83 (-1.52--0.14) |
| Bulgaria | 3 (1-6) | 0.05 (0.01-0.09) | 3 (1-5) | 0.04 (0.01-0.07) | -0.37 (-1.08-0.34) |
| Burkina Faso | 1 (0-3) | 0.06 (0.02-0.1) | 3 (1-6) | 0.05 (0.02-0.1) | -0.32 (-1.22-0.59) |
| Burundi | 1 (0-2) | 0.08 (0.03-0.16) | 2 (1-3) | 0.06 (0.02-0.11) | -1.37 (-2.47--0.27) |
| Cabo Verde | 0 (0-0) | 0.05 (0.02-0.09) | 0 (0-0) | 0.04 (0.01-0.07) | -0.65 (-1.07--0.24) |
| Cambodia | 2 (1-3) | 0.05 (0.02-0.1) | 4 (1-7) | 0.05 (0.02-0.09) | 0.77 (0.22-1.32) |
| Cameroon | 2 (1-4) | 0.07 (0.02-0.13) | 5 (2-10) | 0.07 (0.02-0.12) | -0.18 (-1.12-0.76) |
| Canada | 6 (2-11) | 0.03 (0.01-0.06) | 10 (3-19) | 0.03 (0.01-0.06) | 0.1 (-0.46-0.67) |
| Central African Republic | 0 (0-1) | 0.06 (0.02-0.12) | 1 (0-2) | 0.06 (0.02-0.11) | -0.29 (-1.29-0.72) |
| Chad | 1 (0-2) | 0.07 (0.02-0.13) | 2 (1-4) | 0.06 (0.02-0.12) | -0.71 (-1.82-0.41) |
| Chile | 2 (0-3) | 0.03 (0.01-0.05) | 3 (1-5) | 0.02 (0.01-0.04) | -0.23 (-0.46-0.01) |
| China | 246 (80-459) | 0.05 (0.02-0.1) | 576 (166-1101) | 0.05 (0.02-0.1) | 1.08 (0.8-1.37) |
| Colombia | 2 (1-4) | 0.02 (0.01-0.04) | 4 (1-7) | 0.01 (0-0.02) | -0.81 (-1.15--0.47) |
| Comoros | 0 (0-0) | 0.08 (0.03-0.15) | 0 (0-0) | 0.07 (0.02-0.12) | 0.07 (-0.66-0.81) |
| Congo | 0 (0-1) | 0.08 (0.02-0.15) | 1 (0-2) | 0.07 (0.02-0.13) | -0.33 (-1.2-0.55) |
| Cook Islands | 0 (0-0) | 0.05 (0.02-0.1) | 0 (0-0) | 0.05 (0.01-0.09) | 0.63 (0.36-0.89) |
| Costa Rica | 0 (0-0) | 0.02 (0.01-0.03) | 0 (0-1) | 0.01 (0-0.02) | -0.74 (-1.06--0.42) |
| C么te d'Ivoire | 2 (1-3) | 0.07 (0.02-0.13) | 4 (1-8) | 0.06 (0.02-0.11) | -0.09 (-1.15-0.98) |
| Croatia | 1 (0-2) | 0.03 (0.01-0.06) | 1 (0-2) | 0.02 (0.01-0.04) | -0.99 (-1.73--0.25) |
| Cuba | 1 (0-2) | 0.02 (0.01-0.03) | 1 (0-2) | 0.01 (0-0.02) | -0.36 (-0.74-0.03) |
| Cyprus | 0 (0-0) | 0.01 (0-0.02) | 0 (0-0) | 0.01 (0-0.01) | -1.02 (-1.42--0.61) |
| Czechia | 4 (1-7) | 0.05 (0.01-0.09) | 3 (1-7) | 0.03 (0.01-0.06) | -0.69 (-1.36-0) |
| Democratic People's Republic of Korea | 5 (1-9) | 0.05 (0.01-0.09) | 8 (2-16) | 0.04 (0.01-0.08) | 0.32 (0.09-0.55) |
| Democratic Republic of the Congo | 6 (2-11) | 0.06 (0.02-0.12) | 13 (4-24) | 0.06 (0.02-0.11) | -0.35 (-1.3-0.6) |
| Denmark | 1 (0-2) | 0.02 (0.01-0.04) | 1 (0-2) | 0.02 (0-0.03) | -0.93 (-1.67--0.18) |
| Djibouti | 0 (0-0) | 0.06 (0.02-0.11) | 0 (0-0) | 0.06 (0.02-0.11) | 0.55 (-0.43-1.54) |
| Dominica | 0 (0-0) | 0.02 (0.01-0.04) | 0 (0-0) | 0.02 (0.01-0.03) | -0.22 (-0.38--0.07) |
| Dominican Republic | 0 (0-0) | 0.01 (0-0.02) | 0 (0-1) | 0.01 (0-0.01) | -0.04 (-0.45-0.38) |
| Ecuador | 1 (0-1) | 0.02 (0.01-0.03) | 1 (0-2) | 0.01 (0-0.02) | -0.65 (-1.05--0.24) |
| Egypt | 8 (3-15) | 0.04 (0.01-0.08) | 17 (6-32) | 0.04 (0.01-0.08) | 0.11 (-0.36-0.58) |
| El Salvador | 0 (0-1) | 0.02 (0.01-0.03) | 0 (0-1) | 0.01 (0-0.02) | -0.35 (-0.7--0.01) |
| Equatorial Guinea | 0 (0-0) | 0.07 (0.02-0.13) | 0 (0-0) | 0.05 (0.02-0.1) | -1.15 (-2.16--0.13) |
| Eritrea | 1 (0-1) | 0.07 (0.02-0.13) | 1 (0-2) | 0.06 (0.02-0.11) | 0.18 (-0.77-1.14) |
| Estonia | 1 (0-1) | 0.04 (0.01-0.08) | 0 (0-1) | 0.03 (0.01-0.06) | -0.86 (-1.51--0.21) |
| Eswatini | 0 (0-0) | 0.06 (0.02-0.11) | 0 (0-0) | 0.06 (0.02-0.11) | 0.38 (-0.58-1.34) |
| Ethiopia | 6 (2-12) | 0.06 (0.02-0.1) | 11 (3-20) | 0.04 (0.01-0.08) | -1.01 (-2.14-0.13) |
| Fiji | 0 (0-0) | 0.04 (0.01-0.07) | 0 (0-0) | 0.04 (0.01-0.08) | 1.18 (0.74-1.61) |
| Finland | 1 (0-3) | 0.03 (0.01-0.06) | 2 (0-3) | 0.03 (0.01-0.05) | -0.03 (-0.92-0.87) |
| France | 7 (2-15) | 0.02 (0-0.03) | 9 (3-19) | 0.01 (0-0.03) | -0.3 (-1.07-0.48) |
| Gabon | 0 (0-0) | 0.07 (0.02-0.14) | 0 (0-1) | 0.06 (0.02-0.12) | -0.68 (-1.37-0.02) |
| Gambia | 0 (0-0) | 0.06 (0.02-0.11) | 0 (0-1) | 0.05 (0.02-0.1) | 0.03 (-1.01-1.09) |
| Georgia | 2 (1-4) | 0.07 (0.02-0.12) | 2 (1-3) | 0.06 (0.02-0.11) | 0.01 (-0.44-0.46) |
| Germany | 21 (6-42) | 0.03 (0.01-0.06) | 22 (6-43) | 0.02 (0.01-0.04) | -0.34 (-1.27-0.6) |
| Ghana | 4 (1-7) | 0.09 (0.03-0.17) | 9 (3-17) | 0.08 (0.03-0.16) | -0.13 (-0.9-0.64) |
| Greece | 2 (1-4) | 0.03 (0.01-0.05) | 2 (1-4) | 0.02 (0.01-0.04) | -0.72 (-1.5-0.07) |
| Greenland | 0 (0-0) | 0.04 (0.01-0.08) | 0 (0-0) | 0.02 (0.01-0.04) | -1.97 (-2.24--1.7) |
| Grenada | 0 (0-0) | 0.02 (0.01-0.04) | 0 (0-0) | 0.01 (0-0.02) | -0.96 (-1.18--0.74) |
| Guam | 0 (0-0) | 0.05 (0.02-0.09) | 0 (0-0) | 0.05 (0.02-0.1) | 1.14 (0.85-1.43) |
| Guatemala | 0 (0-1) | 0.02 (0.01-0.03) | 1 (0-1) | 0.01 (0-0.02) | -0.94 (-1.68--0.2) |
| Guinea | 1 (0-2) | 0.06 (0.02-0.12) | 2 (1-4) | 0.06 (0.02-0.11) | -0.45 (-1.27-0.38) |
| Guinea-Bissau | 0 (0-0) | 0.07 (0.02-0.12) | 0 (0-1) | 0.06 (0.02-0.12) | -0.27 (-1.29-0.76) |
| Guyana | 0 (0-0) | 0.03 (0.01-0.05) | 0 (0-0) | 0.02 (0.01-0.04) | -0.26 (-0.71-0.18) |
| Haiti | 0 (0-1) | 0.02 (0.01-0.04) | 1 (0-1) | 0.02 (0.01-0.03) | -0.41 (-1.03-0.22) |
| Honduras | 0 (0-0) | 0.02 (0.01-0.04) | 1 (0-1) | 0.02 (0.01-0.03) | -0.16 (-0.86-0.54) |
| Hungary | 4 (1-8) | 0.06 (0.02-0.11) | 3 (1-6) | 0.03 (0.01-0.06) | -1.71 (-2.4--1.02) |
| Iceland | 0 (0-0) | 0.02 (0.01-0.05) | 0 (0-0) | 0.01 (0-0.03) | -1 (-1.44--0.56) |
| India | 74 (24-137) | 0.03 (0.01-0.05) | 155 (50-288) | 0.02 (0.01-0.04) | 0.04 (-0.31-0.39) |
| Indonesia | 53 (17-103) | 0.09 (0.03-0.17) | 111 (35-212) | 0.08 (0.02-0.15) | 0.4 (0.07-0.73) |
| Iran (Islamic Republic of) | 5 (2-9) | 0.03 (0.01-0.06) | 10 (3-18) | 0.02 (0.01-0.04) | -0.01 (-0.43-0.42) |
| Iraq | 2 (1-4) | 0.05 (0.02-0.09) | 6 (2-10) | 0.04 (0.01-0.07) | -0.39 (-1.04-0.26) |
| Ireland | 0 (0-1) | 0.02 (0.01-0.04) | 0 (0-1) | 0.01 (0-0.02) | -1.98 (-2.34--1.62) |
| Israel | 0 (0-1) | 0.01 (0-0.03) | 1 (0-1) | 0.01 (0-0.02) | -0.67 (-0.89--0.45) |
| Italy | 7 (2-13) | 0.02 (0-0.03) | 7 (2-13) | 0.01 (0-0.02) | -1.11 (-1.96--0.25) |
| Jamaica | 0 (0-0) | 0.02 (0-0.03) | 0 (0-0) | 0.01 (0-0.02) | -0.01 (-0.21-0.18) |
| Japan | 26 (8-49) | 0.03 (0.01-0.05) | 35 (9-71) | 0.02 (0.01-0.04) | -0.17 (-1.09-0.76) |
| Jordan | 0 (0-1) | 0.04 (0.01-0.08) | 1 (0-2) | 0.03 (0.01-0.05) | -0.95 (-1.73--0.17) |
| Kazakhstan | 8 (2-14) | 0.09 (0.03-0.17) | 7 (2-14) | 0.07 (0.02-0.13) | -1.15 (-1.28--1.02) |
| Kenya | 3 (1-5) | 0.06 (0.02-0.11) | 7 (2-14) | 0.05 (0.02-0.1) | -0.04 (-1.07-1.01) |
| Kiribati | 0 (0-0) | 0.09 (0.03-0.17) | 0 (0-0) | 0.08 (0.03-0.15) | -0.02 (-0.54-0.5) |
| Kuwait | 0 (0-0) | 0.03 (0.01-0.05) | 0 (0-1) | 0.02 (0.01-0.04) | -0.25 (-0.7-0.2) |
| Kyrgyzstan | 1 (0-3) | 0.08 (0.02-0.14) | 2 (1-3) | 0.05 (0.02-0.1) | -1.16 (-1.49--0.84) |
| Lao People's Democratic Republic | 1 (0-1) | 0.06 (0.02-0.12) | 2 (1-3) | 0.06 (0.02-0.11) | 0.26 (-0.4-0.92) |
| Latvia | 1 (0-2) | 0.05 (0.01-0.09) | 1 (0-1) | 0.03 (0.01-0.06) | -0.78 (-1.52--0.04) |
| Lebanon | 0 (0-0) | 0.01 (0-0.03) | 1 (0-1) | 0.02 (0.01-0.03) | 1.47 (1.32-1.63) |
| Lesotho | 0 (0-0) | 0.05 (0.01-0.09) | 0 (0-1) | 0.05 (0.02-0.1) | 0.72 (0.24-1.2) |
| Liberia | 0 (0-1) | 0.06 (0.02-0.12) | 1 (0-1) | 0.05 (0.02-0.1) | -0.81 (-1.74-0.14) |
| Libya | 0 (0-0) | 0.02 (0.01-0.04) | 1 (0-2) | 0.03 (0.01-0.05) | 1.15 (0.46-1.85) |
| Lithuania | 1 (0-2) | 0.05 (0.02-0.09) | 1 (0-2) | 0.04 (0.01-0.07) | 0.79 (-0.02-1.61) |
| Luxembourg | 0 (0-0) | 0.02 (0.01-0.04) | 0 (0-0) | 0.01 (0-0.02) | -2.12 (-2.7--1.54) |
| Madagascar | 3 (1-5) | 0.09 (0.03-0.17) | 6 (2-11) | 0.08 (0.02-0.15) | -0.28 (-1.34-0.78) |
| Malawi | 1 (0-3) | 0.06 (0.02-0.12) | 3 (1-5) | 0.05 (0.02-0.1) | -0.61 (-1.67-0.47) |
| Malaysia | 3 (1-5) | 0.05 (0.02-0.09) | 7 (2-13) | 0.05 (0.01-0.09) | 0.47 (0.01-0.92) |
| Maldives | 0 (0-0) | 0.07 (0.02-0.14) | 0 (0-0) | 0.04 (0.01-0.07) | -0.9 (-1.65--0.13) |
| Mali | 1 (0-3) | 0.06 (0.02-0.11) | 3 (1-5) | 0.05 (0.02-0.1) | -1 (-1.98-0) |
| Malta | 0 (0-0) | 0.01 (0-0.03) | 0 (0-0) | 0.01 (0-0.02) | -0.73 (-1.33--0.13) |
| Marshall Islands | 0 (0-0) | 0.07 (0.02-0.13) | 0 (0-0) | 0.07 (0.02-0.12) | 0.85 (0.02-1.69) |
| Mauritania | 0 (0-1) | 0.06 (0.02-0.12) | 1 (0-1) | 0.05 (0.01-0.09) | -1.12 (-1.96--0.28) |
| Mauritius | 0 (0-0) | 0.02 (0.01-0.04) | 0 (0-0) | 0.01 (0-0.03) | -0.06 (-0.35-0.23) |
| Mexico | 5 (2-10) | 0.02 (0.01-0.04) | 10 (3-19) | 0.01 (0-0.03) | -0.31 (-0.72-0.1) |
| Micronesia (Federated States of) | 0 (0-0) | 0.07 (0.02-0.13) | 0 (0-0) | 0.07 (0.02-0.13) | 0.59 (-0.02-1.2) |
| Monaco | 0 (0-0) | 0.02 (0-0.03) | 0 (0-0) | 0.01 (0-0.02) | -1.67 (-2.79--0.55) |
| Mongolia | 0 (0-1) | 0.06 (0.02-0.11) | 1 (0-2) | 0.05 (0.02-0.1) | 0.15 (-0.31-0.62) |
| Montenegro | 0 (0-0) | 0.03 (0.01-0.06) | 0 (0-0) | 0.03 (0.01-0.06) | 0.59 (0.19-1) |
| Morocco | 3 (1-5) | 0.03 (0.01-0.06) | 6 (2-11) | 0.03 (0.01-0.06) | 0.47 (0.14-0.81) |
| Mozambique | 2 (1-4) | 0.05 (0.02-0.1) | 4 (1-7) | 0.05 (0.02-0.1) | -0.42 (-1.44-0.61) |
| Myanmar | 9 (3-16) | 0.06 (0.02-0.11) | 15 (5-28) | 0.05 (0.02-0.1) | -0.14 (-0.46-0.17) |
| Namibia | 0 (0-0) | 0.07 (0.02-0.14) | 0 (0-1) | 0.06 (0.02-0.11) | -0.58 (-1.35-0.19) |
| Nauru | 0 (0-0) | 0.08 (0.03-0.16) | 0 (0-0) | 0.09 (0.03-0.16) | 0.52 (-0.11-1.16) |
| Nepal | 1 (0-3) | 0.03 (0.01-0.05) | 3 (1-5) | 0.02 (0.01-0.03) | -0.52 (-1.08-0.04) |
| Netherlands | 2 (1-5) | 0.02 (0.01-0.04) | 2 (1-4) | 0.01 (0-0.03) | -0.93 (-1.6--0.25) |
| New Zealand | 0 (0-1) | 0.02 (0.01-0.04) | 1 (0-1) | 0.02 (0.01-0.03) | -0.47 (-0.93--0.01) |
| Nicaragua | 0 (0-0) | 0.02 (0.01-0.04) | 0 (0-1) | 0.01 (0-0.03) | -0.19 (-0.85-0.47) |
| Niger | 1 (0-2) | 0.07 (0.02-0.13) | 3 (1-6) | 0.06 (0.02-0.11) | -0.77 (-1.93-0.41) |
| Nigeria | 13 (4-25) | 0.06 (0.02-0.11) | 34 (11-64) | 0.06 (0.02-0.11) | -0.16 (-1.14-0.83) |
| Niue | 0 (0-0) | 0.07 (0.02-0.14) | 0 (0-0) | 0.07 (0.02-0.12) | -0.02 (-0.29-0.24) |
| North Macedonia | 1 (0-1) | 0.06 (0.02-0.12) | 1 (0-1) | 0.04 (0.01-0.09) | -0.56 (-0.91--0.21) |
| Northern Mariana Islands | 0 (0-0) | 0.04 (0.01-0.08) | 0 (0-0) | 0.04 (0.01-0.07) | 1.15 (0.61-1.69) |
| Norway | 1 (0-1) | 0.01 (0-0.03) | 1 (0-1) | 0.01 (0-0.02) | -1.04 (-1.8--0.28) |
| Oman | 0 (0-0) | 0.04 (0.01-0.08) | 1 (0-1) | 0.04 (0.01-0.07) | 0.58 (-0.18-1.34) |
| Pakistan | 12 (4-23) | 0.04 (0.01-0.08) | 31 (10-57) | 0.04 (0.01-0.08) | 0.18 (-0.54-0.91) |
| Palau | 0 (0-0) | 0.07 (0.02-0.14) | 0 (0-0) | 0.07 (0.02-0.13) | 0.92 (0.66-1.17) |
| Palestine | 0 (0-0) | 0.04 (0.01-0.07) | 1 (0-1) | 0.03 (0.01-0.06) | -0.65 (-1.44-0.14) |
| Panama | 0 (0-0) | 0.01 (0-0.02) | 0 (0-0) | 0.01 (0-0.02) | 0.06 (-0.25-0.36) |
| Papua New Guinea | 0 (0-1) | 0.04 (0.01-0.08) | 1 (0-2) | 0.04 (0.01-0.07) | 0.04 (-0.74-0.83) |
| Paraguay | 0 (0-0) | 0.01 (0-0.03) | 0 (0-0) | 0.01 (0-0.01) | -2.01 (-2.47--1.54) |
| Peru | 1 (0-2) | 0.02 (0.01-0.03) | 2 (1-5) | 0.01 (0-0.03) | 0.05 (-0.3-0.4) |
| Philippines | 8 (3-15) | 0.05 (0.01-0.09) | 26 (8-48) | 0.05 (0.02-0.1) | 1.26 (0.73-1.79) |
| Poland | 9 (3-16) | 0.04 (0.01-0.07) | 10 (3-20) | 0.03 (0.01-0.06) | -0.16 (-0.7-0.39) |
| Portugal | 2 (1-4) | 0.03 (0.01-0.05) | 1 (0-2) | 0.01 (0-0.02) | -2.5 (-3.25--1.74) |
| Puerto Rico | 0 (0-0) | 0.01 (0-0.02) | 0 (0-1) | 0.01 (0-0.02) | 0.25 (-0.22-0.73) |
| Qatar | 0 (0-0) | 0.04 (0.01-0.07) | 0 (0-0) | 0.02 (0.01-0.04) | -1.49 (-2.11--0.87) |
| Republic of Korea | 10 (3-19) | 0.06 (0.02-0.11) | 8 (2-16) | 0.02 (0.01-0.03) | -2.85 (-3.28--2.41) |
| Republic of Moldova | 1 (0-2) | 0.04 (0.01-0.08) | 1 (0-2) | 0.04 (0.01-0.07) | 0.07 (-0.36-0.51) |
| Romania | 7 (2-14) | 0.05 (0.02-0.09) | 7 (2-14) | 0.04 (0.01-0.07) | -0.14 (-0.75-0.47) |
| Russian Federation | 62 (19-118) | 0.06 (0.02-0.11) | 60 (18-115) | 0.05 (0.01-0.09) | -0.47 (-0.99-0.05) |
| Rwanda | 2 (0-3) | 0.09 (0.03-0.17) | 2 (1-4) | 0.06 (0.02-0.11) | -1.1 (-2.02--0.16) |
| Saint Kitts and Nevis | 0 (0-0) | 0.03 (0.01-0.05) | 0 (0-0) | 0.01 (0-0.03) | -1.69 (-1.86--1.52) |
| Saint Lucia | 0 (0-0) | 0.03 (0.01-0.05) | 0 (0-0) | 0.02 (0.01-0.03) | -0.21 (-0.51-0.09) |
| Saint Vincent and the Grenadines | 0 (0-0) | 0.03 (0.01-0.05) | 0 (0-0) | 0.02 (0.01-0.03) | -0.78 (-1.04--0.52) |
| Samoa | 0 (0-0) | 0.07 (0.02-0.14) | 0 (0-0) | 0.07 (0.02-0.14) | 0.45 (-0.08-0.98) |
| San Marino | 0 (0-0) | 0.02 (0.01-0.03) | 0 (0-0) | 0.01 (0-0.03) | -0.39 (-1.19-0.42) |
| Sao Tome and Principe | 0 (0-0) | 0.08 (0.02-0.15) | 0 (0-0) | 0.08 (0.02-0.15) | 0.05 (-0.72-0.84) |
| Saudi Arabia | 1 (0-2) | 0.03 (0.01-0.06) | 4 (2-8) | 0.03 (0.01-0.06) | 0.59 (-0.05-1.23) |
| Senegal | 1 (0-2) | 0.06 (0.02-0.11) | 2 (1-4) | 0.05 (0.01-0.09) | -0.61 (-1.48-0.26) |
| Serbia | 3 (1-6) | 0.05 (0.02-0.1) | 3 (1-5) | 0.04 (0.01-0.07) | -0.7 (-1.25--0.15) |
| Seychelles | 0 (0-0) | 0.05 (0.01-0.09) | 0 (0-0) | 0.04 (0.01-0.08) | 0.34 (0.2-0.48) |
| Sierra Leone | 1 (0-2) | 0.07 (0.02-0.14) | 2 (1-3) | 0.07 (0.02-0.13) | -0.41 (-1.27-0.46) |
| Singapore | 1 (0-1) | 0.05 (0.01-0.09) | 1 (0-2) | 0.02 (0.01-0.04) | -1.89 (-2.17--1.61) |
| Slovakia | 2 (1-4) | 0.06 (0.02-0.11) | 2 (1-4) | 0.04 (0.01-0.08) | -0.78 (-1.34--0.22) |
| Slovenia | 0 (0-1) | 0.03 (0.01-0.06) | 0 (0-1) | 0.02 (0.01-0.04) | -1.31 (-2--0.61) |
| Solomon Islands | 0 (0-0) | 0.09 (0.03-0.17) | 0 (0-0) | 0.09 (0.03-0.17) | 0.54 (-0.32-1.42) |
| Somalia | 1 (0-2) | 0.06 (0.02-0.11) | 2 (1-5) | 0.05 (0.02-0.1) | -0.27 (-1.36-0.83) |
| South Africa | 8 (2-15) | 0.06 (0.02-0.12) | 10 (3-20) | 0.04 (0.01-0.07) | -1.38 (-1.74--1.02) |
| South Sudan | 1 (0-1) | 0.06 (0.02-0.11) | 1 (0-2) | 0.05 (0.02-0.09) | -0.27 (-1.46-0.93) |
| Spain | 6 (2-12) | 0.02 (0.01-0.04) | 6 (2-13) | 0.01 (0-0.03) | -1 (-1.78--0.21) |
| Sri Lanka | 3 (1-6) | 0.06 (0.02-0.1) | 7 (2-14) | 0.05 (0.02-0.09) | 0.43 (0.2-0.66) |
| Sudan | 2 (1-4) | 0.04 (0.01-0.07) | 5 (2-10) | 0.04 (0.01-0.08) | 0.11 (-0.67-0.89) |
| Suriname | 0 (0-0) | 0.01 (0-0.03) | 0 (0-0) | 0.01 (0-0.01) | -1.96 (-2.29--1.63) |
| Sweden | 2 (0-4) | 0.02 (0.01-0.04) | 2 (1-4) | 0.02 (0.01-0.04) | -0.53 (-1.4-0.35) |
| Switzerland | 1 (0-2) | 0.02 (0.01-0.03) | 1 (0-2) | 0.01 (0-0.03) | -0.71 (-1.48-0.07) |
| Syrian Arab Republic | 1 (1-3) | 0.04 (0.01-0.08) | 2 (1-4) | 0.03 (0.01-0.05) | -0.42 (-1.07-0.23) |
| Taiwan (Province of China) | 1 (0-3) | 0.02 (0.01-0.04) | 2 (1-4) | 0.01 (0-0.02) | -0.56 (-0.97--0.15) |
| Tajikistan | 1 (0-2) | 0.06 (0.02-0.11) | 2 (1-4) | 0.06 (0.02-0.11) | 0.06 (-0.63-0.75) |
| Thailand | 11 (4-21) | 0.05 (0.02-0.1) | 22 (6-42) | 0.04 (0.01-0.07) | 0.2 (-0.14-0.53) |
| Timor-Leste | 0 (0-0) | 0.05 (0.01-0.09) | 0 (0-0) | 0.05 (0.02-0.09) | 0.71 (-0.01-1.44) |
| Togo | 1 (0-1) | 0.07 (0.02-0.13) | 2 (1-3) | 0.06 (0.02-0.11) | -0.05 (-0.96-0.88) |
| Tokelau | 0 (0-0) | 0.07 (0.02-0.13) | 0 (0-0) | 0.06 (0.02-0.12) | 0.38 (0.23-0.53) |
| Tonga | 0 (0-0) | 0.05 (0.02-0.1) | 0 (0-0) | 0.05 (0.02-0.09) | 0.14 (-0.3-0.58) |
| Trinidad and Tobago | 0 (0-0) | 0.01 (0-0.02) | 0 (0-0) | 0.01 (0-0.01) | -1.19 (-1.43--0.94) |
| Tunisia | 1 (0-1) | 0.02 (0.01-0.04) | 2 (1-3) | 0.02 (0.01-0.05) | 1.47 (1.2-1.75) |
| Turkey | 10 (3-18) | 0.05 (0.01-0.09) | 15 (5-29) | 0.03 (0.01-0.06) | -0.67 (-0.89--0.44) |
| Turkmenistan | 1 (0-2) | 0.08 (0.02-0.14) | 2 (1-4) | 0.09 (0.03-0.17) | 1.38 (0.98-1.79) |
| Tuvalu | 0 (0-0) | 0.07 (0.02-0.13) | 0 (0-0) | 0.07 (0.02-0.12) | 0.05 (-0.1-0.19) |
| Uganda | 2 (1-5) | 0.07 (0.02-0.13) | 5 (2-10) | 0.06 (0.02-0.11) | -0.64 (-1.89-0.62) |
| Ukraine | 25 (8-49) | 0.06 (0.02-0.12) | 20 (6-38) | 0.05 (0.02-0.09) | -0.38 (-1-0.25) |
| United Arab Emirates | 0 (0-0) | 0.04 (0.01-0.07) | 1 (0-2) | 0.04 (0.01-0.07) | 1.16 (0.43-1.9) |
| United Kingdom | 11 (3-22) | 0.02 (0.01-0.04) | 10 (3-21) | 0.02 (0-0.03) | -1.05 (-1.77--0.33) |
| United Republic of Tanzania | 3 (1-6) | 0.05 (0.02-0.1) | 8 (3-15) | 0.05 (0.02-0.1) | 0.23 (-0.76-1.24) |
| United States of America | 30 (8-58) | 0.02 (0.01-0.03) | 32 (9-64) | 0.01 (0-0.02) | -1.5 (-2--0.99) |
| United States Virgin Islands | 0 (0-0) | 0.01 (0-0.02) | 0 (0-0) | 0.01 (0-0.01) | -0.18 (-0.63-0.28) |
| Uruguay | 1 (0-2) | 0.04 (0.01-0.08) | 0 (0-1) | 0.02 (0.01-0.03) | -2.92 (-3.39--2.44) |
| Uzbekistan | 5 (2-10) | 0.07 (0.02-0.14) | 11 (3-20) | 0.07 (0.02-0.13) | 0.26 (-0.17-0.69) |
| Vanuatu | 0 (0-0) | 0.08 (0.03-0.16) | 0 (0-0) | 0.09 (0.03-0.16) | 0.61 (-0.16-1.38) |
| Venezuela (Bolivarian Republic of) | 1 (0-1) | 0.01 (0-0.02) | 2 (0-3) | 0.01 (0-0.02) | 0.16 (-0.22-0.55) |
| Viet Nam | 13 (4-25) | 0.05 (0.02-0.1) | 29 (9-53) | 0.05 (0.02-0.09) | 0.52 (0.23-0.81) |
| Yemen | 1 (0-2) | 0.04 (0.01-0.07) | 4 (1-7) | 0.04 (0.01-0.07) | 0.42 (-0.5-1.35) |
| Zambia | 1 (0-2) | 0.07 (0.02-0.13) | 3 (1-5) | 0.06 (0.02-0.12) | -0.08 (-1.34-1.18) |

Abbreviations: IS,Ischemic stroke,IIPUFAs, insufficient intake of polyunsaturated fatty acids,EAPC, estimated annual percentage change, SDl, Sociodemographic Index; Ul,uncertainty interval. “ EAPC is expressed as 95% CIs.

Table 4 The YLLs cases and age-standardized YLLs rate of ischemic stroke attributed to IIPUFAs disease in 1990 and 2021, along with their temporal trend.

|  | Rate per 100 000(95%UI) | | 2021 | | 1990-2021 |
| --- | --- | --- | --- | --- | --- |
|  | 1990 | |  | |  |
|  | YLLs cases | The age-standardized YLLs rate | YLLs cases | The age-standardized YLLs rate | EAPC |

| **Global** | 5132 (1459-9815) | 0.25 (0.07-0.48) | 6286 (1773-12283) | 0.14 (0.04-0.26) | -1.61 (-1.82--1.4) |
| --- | --- | --- | --- | --- | --- |
| **Age** |  |  |  |  |  |
| 50-54 years | 280 (98-478) | 0.27 (0.09-0.46) | 313 (113-548) | 0.14 (0.05-0.25) | -2.27 (-2.43--2.11) |
| 55-59 years | 376 (124-672) | 0.41 (0.13-0.73) | 434 (148-782) | 0.22 (0.07-0.39) | -2.46 (-2.61--2.3) |
| 60-64 years | 609 (185-1124) | 0.74 (0.23-1.37) | 643 (199-1188) | 0.39 (0.12-0.72) | -2.44 (-2.6--2.28) |
| 65-69 years | 634 (181-1213) | 0.96 (0.27-1.83) | 772 (223-1490) | 0.54 (0.16-1.03) | -2.47 (-2.71--2.23) |
| 70-74 years | 743 (193-1484) | 1.58 (0.41-3.16) | 960 (252-1920) | 0.88 (0.23-1.75) | -2.32 (-2.55--2.09) |
| 75-79 years | 780 (194-1606) | 2.15 (0.53-4.42) | 800 (203-1649) | 1.11 (0.28-2.29) | -2.21 (-2.37--2.06) |
| 80-84 years | 673 (155-1428) | 3.05 (0.7-6.46) | 853 (190-1810) | 1.68 (0.37-3.55) | -2.13 (-2.21--2.05) |
| 85-89 years | 394 (87-846) | 3.92 (0.87-8.42) | 590 (130-1275) | 2.07 (0.46-4.48) | -2.25 (-2.37--2.13) |
| 90-94 years | 148 (32-314) | 4.88 (1.04-10.36) | 319 (68-687) | 2.64 (0.56-5.7) | -2.3 (-2.46--2.13) |
| 95+ years | 45 (9-94) | 5.88 (1.25-12.42) | 129 (27-278) | 3.29 (0.69-7.05) | -2.18 (-2.33--2.03) |
| **SDI region** |  |  |  |  |  |
| High SDI | 839 (218-1663) | 0.12 (0.03-0.24) | 491 (121-991) | 0.04 (0.01-0.07) | -4.23 (-4.39--4.08) |
| High-middle SDI | 1980 (557-3829) | 0.37 (0.1-0.71) | 1827 (483-3683) | 0.16 (0.04-0.32) | -3.2 (-3.49--2.9) |
| Middle SDI | 1284 (373-2494) | 0.26 (0.07-0.52) | 2106 (607-4156) | 0.15 (0.04-0.31) | -1.82 (-1.92--1.73) |
| Low-middle SDI | 747 (239-1445) | 0.27 (0.08-0.52) | 1377 (405-2688) | 0.19 (0.06-0.39) | -1.1 (-1.15--1.05) |
| Low SDI | 275 (84-554) | 0.27 (0.08-0.57) | 478 (135-970) | 0.2 (0.05-0.43) | -1.06 (-1.12--0.99) |
| **GBD region** |  |  |  |  |  |
| Advanced Health System | 2150 (571-4168) | 0.21 (0.06-0.41) | 1309 (322-2595) | 0.07 (0.02-0.14) | -3.42 (-4.26--2.57) |
| Africa | 430 (134-825) | 0.33 (0.1-0.64) | 758 (220-1440) | 0.25 (0.07-0.5) | -0.79 (-1.98-0.41) |
| African Region | 261 (80-531) | 0.26 (0.08-0.53) | 483 (139-958) | 0.21 (0.06-0.42) | -0.77 (-2.04-0.52) |
| America | 257 (68-488) | 0.07 (0.02-0.14) | 256 (66-490) | 0.03 (0.01-0.06) | -2.11 (-2.4--1.81) |
| Andean Latin America | 10 (3-19) | 0.1 (0.03-0.19) | 15 (4-28) | 0.05 (0.01-0.09) | -1.92 (-2.46--1.38) |
| Asia | 2543 (752-4795) | 0.27 (0.08-0.51) | 4140 (1184-8176) | 0.16 (0.05-0.32) | -0.81 (-1.17--0.44) |
| Australasia | 15 (4-31) | 0.11 (0.03-0.22) | 11 (2-23) | 0.03 (0.01-0.06) | -3.53 (-4.37--2.68) |
| Basic Health System | 2035 (592-3930) | 0.28 (0.08-0.56) | 3209 (926-6277) | 0.17 (0.05-0.33) | -0.72 (-1.08--0.36) |
| Caribbean | 23 (7-44) | 0.17 (0.05-0.34) | 29 (8-55) | 0.1 (0.03-0.19) | -1 (-1.23--0.77) |
| Central Africa | 41 (12-83) | 0.32 (0.09-0.64) | 78 (21-167) | 0.27 (0.07-0.58) | -0.88 (-2.21-0.47) |
| Central Asia | 92 (26-173) | 0.34 (0.1-0.63) | 102 (29-194) | 0.24 (0.07-0.45) | -1.63 (-2--1.26) |
| Central Europe | 341 (93-660) | 0.41 (0.11-0.78) | 227 (59-445) | 0.15 (0.04-0.3) | -2.46 (-3.28--1.65) |
| Central Latin America | 48 (14-88) | 0.12 (0.03-0.22) | 64 (18-123) | 0.05 (0.01-0.09) | -1.81 (-2.41--1.2) |
| Central Sub-Saharan Africa | 30 (8-59) | 0.31 (0.08-0.62) | 61 (16-133) | 0.25 (0.06-0.55) | -0.87 (-2.23-0.51) |
| Commonwealth High Income | 120 (30-242) | 0.12 (0.03-0.24) | 53 (12-110) | 0.03 (0.01-0.06) | -4.34 (-5.31--3.36) |
| Commonwealth Low Income | 112 (34-216) | 0.3 (0.09-0.6) | 240 (68-484) | 0.24 (0.06-0.49) | -0.05 (-1.33-1.24) |
| Commonwealth Middle Income | 584 (182-1179) | 0.21 (0.06-0.43) | 1137 (327-2204) | 0.15 (0.04-0.29) | -0.63 (-1.37-0.12) |
| East Asia | 1266 (356-2488) | 0.3 (0.08-0.61) | 1901 (527-3913) | 0.17 (0.05-0.35) | -0.75 (-1.1--0.4) |
| East Asia & Pacific - WB | 1787 (505-3446) | 0.27 (0.08-0.54) | 2730 (770-5465) | 0.16 (0.04-0.31) | -0.73 (-1.04--0.42) |
| Eastern Africa | 89 (28-177) | 0.28 (0.08-0.57) | 144 (42-294) | 0.19 (0.05-0.4) | -1.3 (-2.68-0.09) |
| Eastern Europe | 987 (268-1902) | 0.54 (0.15-1.04) | 605 (152-1203) | 0.25 (0.07-0.5) | -2.87 (-3.7--2.04) |
| Eastern Mediterranean Region | 353 (107-660) | 0.44 (0.13-0.84) | 609 (190-1137) | 0.3 (0.09-0.57) | -1.01 (-1.97--0.03) |
| Eastern Sub-Saharan Africa | 82 (23-165) | 0.25 (0.07-0.51) | 142 (40-291) | 0.18 (0.05-0.38) | -1.06 (-2.51-0.41) |
| Europe | 1893 (507-3667) | 0.29 (0.08-0.55) | 1125 (281-2231) | 0.11 (0.03-0.21) | -3.18 (-4.06--2.29) |
| Europe & Central Asia - WB | 1959 (526-3788) | 0.29 (0.08-0.56) | 1196 (304-2350) | 0.11 (0.03-0.22) | -3.12 (-3.94--2.29) |
| European Region | 1966 (528-3802) | 0.29 (0.08-0.56) | 1205 (306-2366) | 0.11 (0.03-0.22) | -3.11 (-3.93--2.29) |
| High-income Asia Pacific | 148 (38-290) | 0.13 (0.03-0.26) | 90 (19-189) | 0.02 (0.01-0.04) | -4.27 (-5.48--3.04) |
| High-income North America | 93 (22-186) | 0.04 (0.01-0.08) | 73 (17-149) | 0.02 (0-0.03) | -2.85 (-3.74--1.95) |
| Latin America & Caribbean - WB | 165 (46-306) | 0.13 (0.03-0.23) | 184 (49-348) | 0.05 (0.01-0.09) | -2.09 (-2.49--1.7) |
| Limited Health System | 847 (265-1630) | 0.24 (0.07-0.48) | 1600 (452-3091) | 0.17 (0.05-0.33) | -0.74 (-1.59-0.12) |
| Middle East & North Africa - WB | 265 (80-504) | 0.47 (0.13-0.91) | 451 (133-857) | 0.28 (0.08-0.54) | -0.96 (-1.78--0.13) |
| Minimal Health System | 92 (27-183) | 0.34 (0.09-0.68) | 161 (45-337) | 0.28 (0.07-0.59) | -0.93 (-2.26-0.42) |
| North Africa and Middle East | 363 (109-673) | 0.46 (0.13-0.88) | 585 (171-1113) | 0.27 (0.08-0.53) | -1.06 (-1.82--0.3) |
| North America | 93 (22-186) | 0.04 (0.01-0.08) | 73 (17-149) | 0.02 (0-0.03) | -2.85 (-3.74--1.95) |
| Northern Africa | 160 (48-325) | 0.58 (0.16-1.16) | 269 (79-518) | 0.39 (0.11-0.76) | -0.49 (-1.3-0.32) |
| Oceania | 3 (1-5) | 0.23 (0.06-0.45) | 6 (2-12) | 0.19 (0.05-0.39) | -0.42 (-1.6-0.78) |
| Region of the Americas | 257 (68-488) | 0.07 (0.02-0.14) | 256 (66-490) | 0.03 (0.01-0.06) | -2.11 (-2.4--1.81) |
| South-East Asia Region | 761 (244-1479) | 0.24 (0.07-0.47) | 1521 (443-3060) | 0.17 (0.05-0.35) | -0.31 (-0.95-0.33) |
| South Asia | 544 (169-1114) | 0.22 (0.07-0.44) | 1087 (312-2160) | 0.15 (0.04-0.31) | -0.48 (-1.22-0.25) |
| South Asia - WB | 579 (183-1168) | 0.23 (0.07-0.46) | 1149 (332-2256) | 0.16 (0.05-0.31) | -0.5 (-1.23-0.23) |
| Southeast Asia | 369 (112-712) | 0.3 (0.09-0.59) | 752 (216-1432) | 0.23 (0.06-0.45) | 0.11 (-0.46-0.69) |
| Southern Africa | 42 (13-82) | 0.19 (0.06-0.38) | 91 (26-176) | 0.19 (0.05-0.38) | 0.23 (-0.78-1.25) |
| Southern Latin America | 36 (10-69) | 0.14 (0.04-0.27) | 23 (6-46) | 0.04 (0.01-0.08) | -2.93 (-3.31--2.56) |
| Southern Sub-Saharan Africa | 23 (7-46) | 0.16 (0.04-0.31) | 49 (13-96) | 0.16 (0.04-0.32) | 0.84 (0.11-1.57) |
| Sub-Saharan Africa - WB | 271 (84-547) | 0.27 (0.08-0.55) | 490 (140-969) | 0.21 (0.06-0.43) | -0.84 (-2.13-0.47) |
| Tropical Latin America | 48 (13-93) | 0.11 (0.03-0.22) | 54 (14-109) | 0.04 (0.01-0.08) | -2.22 (-2.63--1.81) |
| Western Africa | 97 (28-211) | 0.28 (0.08-0.6) | 175 (49-345) | 0.22 (0.06-0.46) | -0.95 (-2.3-0.43) |
| Western Europe | 503 (123-1016) | 0.13 (0.03-0.26) | 211 (45-439) | 0.03 (0.01-0.06) | -4.15 (-5.37--2.92) |
| Western Pacific Region | 1522 (423-2974) | 0.27 (0.07-0.53) | 2203 (610-4509) | 0.14 (0.04-0.29) | -0.89 (-1.23--0.56) |
| Western Sub-Saharan Africa | 108 (31-232) | 0.28 (0.08-0.6) | 199 (55-388) | 0.23 (0.06-0.46) | -0.86 (-2.22-0.51) |
| World Bank High Income | 1074 (274-2123) | 0.13 (0.03-0.26) | 583 (138-1170) | 0.04 (0.01-0.07) | -3.68 (-4.63--2.71) |
| World Bank Low Income | 213 (65-406) | 0.3 (0.09-0.6) | 362 (101-711) | 0.23 (0.06-0.46) | -1.13 (-2.31-0.06) |
| World Bank Lower Middle Income | 1453 (448-2779) | 0.31 (0.09-0.61) | 2488 (731-4950) | 0.21 (0.06-0.42) | -0.84 (-1.57--0.1) |
| World Bank Upper Middle Income | 2385 (667-4673) | 0.32 (0.09-0.64) | 2847 (780-5782) | 0.15 (0.04-0.31) | -1.77 (-2.08--1.45) |
| **Countries** |  |  |  |  |  |
| Afghanistan | 22 (6-44) | 0.68 (0.18-1.4) | 32 (10-70) | 0.58 (0.16-1.27) | -1.57 (-2.7--0.44) |
| Albania | 1 (0-3) | 0.15 (0.04-0.3) | 3 (1-6) | 0.12 (0.03-0.25) | 1.63 (1.13-2.13) |
| Algeria | 21 (6-43) | 0.45 (0.11-0.96) | 37 (10-77) | 0.27 (0.07-0.55) | -0.76 (-1.68-0.17) |
| American Samoa | 0 (0-0) | 0.15 (0.04-0.3) | 0 (0-0) | 0.14 (0.04-0.27) | 0.79 (-0.06-1.65) |
| Andorra | 0 (0-0) | 0.06 (0.01-0.13) | 0 (0-0) | 0.03 (0.01-0.05) | -1.02 (-1.76--0.27) |
| Angola | 3 (1-7) | 0.22 (0.06-0.43) | 10 (3-20) | 0.19 (0.05-0.38) | -0.78 (-2.37-0.84) |
| Antigua and Barbuda | 0 (0-0) | 0.08 (0.02-0.15) | 0 (0-0) | 0.06 (0.02-0.12) | -0.87 (-1.09--0.64) |
| Argentina | 24 (6-46) | 0.13 (0.04-0.26) | 14 (3-28) | 0.04 (0.01-0.08) | -2.98 (-3.37--2.59) |
| Armenia | 5 (1-9) | 0.31 (0.09-0.59) | 4 (1-8) | 0.16 (0.04-0.31) | -2.04 (-2.5--1.59) |
| Australia | 13 (3-26) | 0.11 (0.03-0.23) | 9 (2-19) | 0.03 (0.01-0.06) | -3.67 (-4.53--2.8) |
| Austria | 13 (3-28) | 0.16 (0.04-0.32) | 4 (1-8) | 0.03 (0.01-0.05) | -5.62 (-6.83--4.39) |
| Azerbaijan | 5 (1-10) | 0.19 (0.05-0.37) | 7 (2-15) | 0.14 (0.04-0.31) | -0.62 (-0.99--0.25) |
| Bahamas | 0 (0-0) | 0.08 (0.02-0.15) | 0 (0-0) | 0.06 (0.02-0.12) | 0.24 (-0.13-0.61) |
| Bahrain | 0 (0-0) | 0.29 (0.08-0.56) | 0 (0-1) | 0.14 (0.04-0.29) | -2.42 (-3.87--0.95) |
| Bangladesh | 68 (20-134) | 0.36 (0.1-0.74) | 157 (44-316) | 0.27 (0.07-0.54) | 0.44 (-0.77-1.66) |
| Barbados | 0 (0-1) | 0.17 (0.05-0.33) | 0 (0-1) | 0.11 (0.03-0.21) | -0.92 (-1.57--0.27) |
| Belarus | 35 (9-66) | 0.41 (0.11-0.78) | 22 (6-45) | 0.2 (0.05-0.4) | -2.66 (-3.52--1.79) |
| Belgium | 10 (2-21) | 0.1 (0.02-0.2) | 4 (1-8) | 0.02 (0.01-0.05) | -3.95 (-5.16--2.72) |
| Belize | 0 (0-0) | 0.09 (0.02-0.18) | 0 (0-0) | 0.06 (0.02-0.12) | -1.45 (-2.35--0.54) |
| Benin | 3 (1-7) | 0.33 (0.09-0.72) | 7 (2-13) | 0.28 (0.07-0.57) | -0.61 (-1.99-0.79) |
| Bermuda | 0 (0-0) | 0.11 (0.03-0.21) | 0 (0-0) | 0.03 (0.01-0.07) | -2.75 (-3.51--1.99) |
| Bhutan | 0 (0-0) | 0.22 (0.06-0.45) | 0 (0-1) | 0.13 (0.04-0.27) | -0.83 (-1.69-0.04) |
| Bolivia (Plurinational State of) | 2 (1-5) | 0.15 (0.04-0.31) | 3 (1-7) | 0.08 (0.02-0.17) | -1.44 (-2.14--0.74) |
| Bosnia and Herzegovina | 10 (3-19) | 0.49 (0.13-0.93) | 10 (3-21) | 0.28 (0.08-0.56) | -0.83 (-1.52--0.12) |
| Botswana | 1 (0-1) | 0.28 (0.07-0.57) | 1 (0-2) | 0.17 (0.04-0.34) | -0.3 (-1.42-0.83) |
| Brazil | 47 (12-90) | 0.11 (0.03-0.21) | 53 (14-106) | 0.04 (0.01-0.08) | -2.21 (-2.61--1.8) |
| Brunei Darussalam | 0 (0-0) | 0.27 (0.07-0.55) | 0 (0-0) | 0.12 (0.03-0.23) | -1.82 (-3.03--0.59) |
| Bulgaria | 27 (8-52) | 0.47 (0.13-0.91) | 26 (7-52) | 0.29 (0.08-0.58) | -0.5 (-1.41-0.42) |
| Burkina Faso | 3 (1-6) | 0.14 (0.04-0.31) | 6 (2-14) | 0.15 (0.04-0.31) | 0.27 (-1.04-1.6) |
| Burundi | 6 (2-12) | 0.49 (0.13-1.03) | 5 (1-12) | 0.26 (0.07-0.58) | -3.33 (-4.74--1.89) |
| Cabo Verde | 0 (0-0) | 0.15 (0.04-0.33) | 0 (0-1) | 0.12 (0.03-0.24) | -0.42 (-0.81--0.02) |
| Cambodia | 8 (3-15) | 0.35 (0.1-0.69) | 17 (5-35) | 0.27 (0.08-0.57) | -0.09 (-0.99-0.82) |
| Cameroon | 5 (1-10) | 0.25 (0.07-0.52) | 15 (4-30) | 0.26 (0.07-0.55) | 0.19 (-1.21-1.6) |
| Canada | 16 (4-32) | 0.08 (0.02-0.16) | 14 (3-28) | 0.03 (0.01-0.06) | -2.87 (-3.79--1.94) |
| Central African Republic | 2 (1-4) | 0.4 (0.11-0.83) | 3 (1-7) | 0.34 (0.08-0.71) | -0.83 (-2.27-0.64) |
| Chad | 4 (1-9) | 0.27 (0.07-0.64) | 7 (2-15) | 0.29 (0.08-0.64) | -0.57 (-2.06-0.94) |
| Chile | 8 (2-15) | 0.15 (0.04-0.3) | 7 (2-13) | 0.04 (0.01-0.08) | -2.41 (-2.77--2.06) |
| China | 1242 (349-2437) | 0.31 (0.09-0.62) | 1864 (516-3843) | 0.17 (0.05-0.36) | -0.75 (-1.1--0.4) |
| Colombia | 12 (4-23) | 0.14 (0.04-0.26) | 12 (3-23) | 0.04 (0.01-0.07) | -3.09 (-3.63--2.55) |
| Comoros | 0 (0-1) | 0.32 (0.09-0.66) | 0 (0-1) | 0.21 (0.05-0.43) | -0.87 (-1.87-0.14) |
| Congo | 2 (1-4) | 0.37 (0.1-0.74) | 4 (1-7) | 0.31 (0.08-0.63) | -0.85 (-2.04-0.34) |
| Cook Islands | 0 (0-0) | 0.14 (0.04-0.29) | 0 (0-0) | 0.07 (0.02-0.14) | -1 (-1.44--0.55) |
| Costa Rica | 1 (0-1) | 0.08 (0.02-0.16) | 1 (0-2) | 0.03 (0.01-0.06) | -2.26 (-2.81--1.71) |
| C么te d'Ivoire | 4 (1-8) | 0.27 (0.08-0.54) | 11 (3-23) | 0.26 (0.07-0.53) | 0.79 (-0.88-2.49) |
| Croatia | 11 (3-22) | 0.32 (0.08-0.63) | 5 (1-11) | 0.08 (0.02-0.17) | -3.5 (-4.51--2.48) |
| Cuba | 7 (2-14) | 0.15 (0.04-0.29) | 9 (3-19) | 0.09 (0.02-0.17) | -0.66 (-1.18--0.15) |
| Cyprus | 1 (0-1) | 0.18 (0.04-0.37) | 0 (0-1) | 0.05 (0.01-0.1) | -3.8 (-4.13--3.46) |
| Czechia | 38 (10-77) | 0.44 (0.11-0.87) | 10 (2-20) | 0.07 (0.02-0.14) | -5.59 (-6.53--4.63) |
| Democratic People's Republic of Korea | 20 (6-38) | 0.21 (0.06-0.42) | 35 (10-70) | 0.18 (0.05-0.37) | 0.14 (-0.15-0.44) |
| Democratic Republic of the Congo | 22 (6-44) | 0.32 (0.08-0.65) | 43 (10-99) | 0.26 (0.06-0.6) | -0.76 (-2.08-0.57) |
| Denmark | 5 (1-11) | 0.1 (0.03-0.2) | 3 (1-6) | 0.03 (0.01-0.07) | -3.45 (-4.56--2.32) |
| Djibouti | 0 (0-0) | 0.19 (0.05-0.41) | 0 (0-1) | 0.16 (0.05-0.35) | -0.1 (-1.61-1.44) |
| Dominica | 0 (0-0) | 0.22 (0.06-0.43) | 0 (0-0) | 0.16 (0.04-0.32) | -0.42 (-0.7--0.13) |
| Dominican Republic | 1 (0-3) | 0.08 (0.02-0.16) | 2 (1-4) | 0.04 (0.01-0.08) | -0.38 (-0.98-0.22) |
| Ecuador | 3 (1-5) | 0.11 (0.03-0.21) | 3 (1-6) | 0.04 (0.01-0.08) | -2.51 (-3.09--1.94) |
| Egypt | 101 (28-215) | 0.93 (0.24-2) | 156 (47-302) | 0.71 (0.2-1.37) | -0.39 (-1.59-0.83) |
| El Salvador | 2 (0-3) | 0.1 (0.03-0.18) | 2 (0-4) | 0.05 (0.01-0.09) | -1.23 (-1.7--0.77) |
| Equatorial Guinea | 0 (0-1) | 0.33 (0.1-0.7) | 1 (0-1) | 0.23 (0.06-0.51) | -1.71 (-3.09--0.3) |
| Eritrea | 2 (0-4) | 0.32 (0.09-0.71) | 3 (1-6) | 0.23 (0.06-0.48) | -0.46 (-1.89-0.99) |
| Estonia | 6 (2-12) | 0.43 (0.11-0.84) | 1 (0-2) | 0.05 (0.01-0.11) | -7.44 (-8.53--6.34) |
| Eswatini | 0 (0-1) | 0.27 (0.07-0.56) | 1 (0-2) | 0.29 (0.07-0.62) | 1.31 (-0.1-2.75) |
| Ethiopia | 17 (4-42) | 0.2 (0.05-0.47) | 24 (7-51) | 0.12 (0.04-0.26) | -1.83 (-3.37--0.25) |
| Fiji | 0 (0-0) | 0.09 (0.02-0.18) | 0 (0-1) | 0.09 (0.03-0.19) | 1.26 (0.43-2.1) |
| Finland | 8 (2-17) | 0.17 (0.04-0.34) | 4 (1-9) | 0.04 (0.01-0.09) | -3.39 (-4.6--2.15) |
| France | 52 (12-106) | 0.09 (0.02-0.17) | 27 (6-59) | 0.02 (0-0.05) | -3.47 (-4.74--2.18) |
| Gabon | 1 (0-1) | 0.26 (0.06-0.5) | 1 (0-2) | 0.22 (0.06-0.49) | -1.05 (-1.93--0.16) |
| Gambia | 0 (0-1) | 0.24 (0.06-0.5) | 1 (0-3) | 0.27 (0.07-0.63) | 1.03 (-0.38-2.46) |
| Georgia | 10 (3-20) | 0.27 (0.07-0.53) | 11 (3-22) | 0.27 (0.07-0.53) | 0.42 (-0.47-1.32) |
| Germany | 124 (30-250) | 0.14 (0.03-0.27) | 43 (10-88) | 0.03 (0.01-0.06) | -3.98 (-5.25--2.7) |
| Ghana | 11 (3-22) | 0.38 (0.1-0.77) | 27 (7-57) | 0.33 (0.08-0.7) | -0.07 (-1.24-1.1) |
| Greece | 23 (6-46) | 0.27 (0.07-0.55) | 13 (3-27) | 0.07 (0.02-0.13) | -3.87 (-4.93--2.79) |
| Greenland | 0 (0-0) | 0.21 (0.06-0.43) | 0 (0-0) | 0.05 (0.01-0.1) | -4.19 (-4.8--3.57) |
| Grenada | 0 (0-0) | 0.28 (0.08-0.54) | 0 (0-0) | 0.1 (0.03-0.19) | -2.72 (-3.1--2.34) |
| Guam | 0 (0-0) | 0.13 (0.04-0.26) | 0 (0-0) | 0.04 (0.01-0.09) | -1.44 (-2.01--0.86) |
| Guatemala | 2 (1-4) | 0.12 (0.03-0.23) | 3 (1-5) | 0.05 (0.01-0.09) | -2.61 (-3.71--1.49) |
| Guinea | 5 (1-10) | 0.3 (0.08-0.67) | 7 (2-15) | 0.29 (0.08-0.61) | -0.19 (-1.24-0.87) |
| Guinea-Bissau | 1 (0-1) | 0.35 (0.09-0.75) | 1 (0-2) | 0.35 (0.09-0.74) | 0.05 (-1.42-1.54) |
| Guyana | 1 (0-2) | 0.48 (0.13-0.95) | 1 (0-2) | 0.26 (0.07-0.52) | -0.31 (-1.06-0.45) |
| Haiti | 7 (2-15) | 0.48 (0.12-0.94) | 11 (3-23) | 0.32 (0.08-0.68) | -1.15 (-2.16--0.13) |
| Honduras | 2 (1-4) | 0.18 (0.05-0.35) | 6 (2-13) | 0.2 (0.05-0.41) | 0.73 (-0.26-1.73) |
| Hungary | 33 (9-63) | 0.38 (0.1-0.72) | 12 (3-25) | 0.09 (0.03-0.19) | -4.09 (-5.04--3.13) |
| Iceland | 0 (0-0) | 0.08 (0.02-0.17) | 0 (0-0) | 0.02 (0.01-0.05) | -3.48 (-4.25--2.7) |
| India | 406 (126-842) | 0.2 (0.06-0.41) | 796 (227-1603) | 0.13 (0.04-0.27) | -0.52 (-1.15-0.11) |
| Indonesia | 160 (47-325) | 0.34 (0.1-0.71) | 380 (106-773) | 0.36 (0.09-0.73) | 0.84 (0.15-1.52) |
| Iran (Islamic Republic of) | 35 (10-65) | 0.3 (0.09-0.59) | 50 (14-92) | 0.14 (0.04-0.26) | -1.55 (-2.34--0.76) |
| Iraq | 23 (7-43) | 0.56 (0.16-1.06) | 41 (13-77) | 0.36 (0.1-0.68) | -1.67 (-2.62--0.72) |
| Ireland | 4 (1-7) | 0.15 (0.04-0.29) | 1 (0-3) | 0.03 (0.01-0.05) | -5.03 (-5.65--4.41) |
| Israel | 2 (0-3) | 0.06 (0.02-0.12) | 1 (0-2) | 0.01 (0-0.03) | -4.42 (-4.84--4.01) |
| Italy | 69 (17-140) | 0.12 (0.03-0.25) | 38 (8-82) | 0.03 (0.01-0.06) | -3.66 (-5.08--2.21) |
| Jamaica | 2 (1-4) | 0.21 (0.06-0.4) | 2 (1-4) | 0.12 (0.03-0.24) | -0.84 (-1.2--0.48) |
| Japan | 105 (26-212) | 0.11 (0.03-0.22) | 72 (15-153) | 0.02 (0-0.04) | -3.67 (-5.13--2.18) |
| Jordan | 3 (1-5) | 0.47 (0.13-0.9) | 4 (1-9) | 0.15 (0.04-0.31) | -3.94 (-5.27--2.6) |
| Kazakhstan | 33 (9-63) | 0.43 (0.11-0.82) | 25 (7-48) | 0.26 (0.07-0.5) | -2.45 (-2.88--2.03) |
| Kenya | 6 (2-14) | 0.17 (0.05-0.38) | 16 (4-35) | 0.16 (0.04-0.35) | 0.7 (-0.71-2.13) |
| Kiribati | 0 (0-0) | 0.17 (0.05-0.35) | 0 (0-0) | 0.17 (0.05-0.35) | 0.14 (-0.78-1.08) |
| Kuwait | 0 (0-1) | 0.12 (0.03-0.23) | 0 (0-1) | 0.04 (0.01-0.07) | -2.98 (-4.24--1.71) |
| Kyrgyzstan | 8 (2-15) | 0.46 (0.13-0.86) | 6 (2-12) | 0.24 (0.07-0.44) | -3.03 (-3.55--2.52) |
| Lao People's Democratic Republic | 6 (2-11) | 0.56 (0.16-1.1) | 7 (2-13) | 0.31 (0.08-0.6) | -1.84 (-2.77--0.89) |
| Latvia | 10 (3-20) | 0.42 (0.11-0.82) | 6 (1-12) | 0.17 (0.04-0.35) | -2.54 (-3.65--1.42) |
| Lebanon | 1 (0-3) | 0.13 (0.03-0.27) | 2 (0-3) | 0.05 (0.01-0.1) | -2.71 (-3.02--2.39) |
| Lesotho | 1 (0-2) | 0.2 (0.05-0.42) | 2 (1-4) | 0.36 (0.09-0.75) | 3.06 (2.25-3.87) |
| Liberia | 1 (0-3) | 0.3 (0.08-0.63) | 3 (1-6) | 0.29 (0.08-0.65) | -0.73 (-2.06-0.61) |
| Libya | 1 (0-3) | 0.16 (0.04-0.32) | 5 (1-9) | 0.17 (0.04-0.34) | 1.31 (0.38-2.25) |
| Lithuania | 7 (2-15) | 0.26 (0.07-0.51) | 5 (1-10) | 0.11 (0.03-0.22) | -1.94 (-2.97--0.91) |
| Luxembourg | 1 (0-1) | 0.2 (0.05-0.4) | 0 (0-0) | 0.03 (0.01-0.05) | -5.79 (-6.71--4.86) |
| Madagascar | 8 (2-17) | 0.36 (0.1-0.73) | 16 (4-34) | 0.31 (0.08-0.64) | -0.78 (-2.21-0.68) |
| Malawi | 5 (1-10) | 0.29 (0.07-0.58) | 10 (2-19) | 0.27 (0.07-0.54) | -0.6 (-1.97-0.8) |
| Malaysia | 9 (2-18) | 0.2 (0.05-0.4) | 17 (5-33) | 0.13 (0.04-0.27) | -0.17 (-0.96-0.62) |
| Maldives | 0 (0-0) | 0.41 (0.12-0.77) | 0 (0-0) | 0.12 (0.03-0.24) | -3.17 (-4.34--1.98) |
| Mali | 6 (2-12) | 0.32 (0.09-0.71) | 10 (3-23) | 0.26 (0.07-0.59) | -0.91 (-2.31-0.5) |
| Malta | 0 (0-0) | 0.1 (0.03-0.2) | 0 (0-0) | 0.03 (0.01-0.05) | -3.5 (-4.42--2.58) |
| Marshall Islands | 0 (0-0) | 0.24 (0.06-0.49) | 0 (0-0) | 0.23 (0.05-0.49) | 0.38 (-0.95-1.73) |
| Mauritania | 1 (0-3) | 0.29 (0.08-0.65) | 2 (1-5) | 0.21 (0.06-0.47) | -1.23 (-2.36--0.09) |
| Mauritius | 0 (0-1) | 0.11 (0.03-0.22) | 0 (0-1) | 0.04 (0.01-0.08) | -3.22 (-3.69--2.74) |
| Mexico | 25 (7-45) | 0.13 (0.04-0.25) | 31 (9-62) | 0.05 (0.01-0.1) | -1.9 (-2.57--1.23) |
| Micronesia (Federated States of) | 0 (0-0) | 0.28 (0.07-0.56) | 0 (0-0) | 0.22 (0.06-0.47) | -0.25 (-1.13-0.64) |
| Monaco | 0 (0-0) | 0.11 (0.02-0.22) | 0 (0-0) | 0.04 (0.01-0.08) | -3.4 (-5.02--1.75) |
| Mongolia | 1 (0-1) | 0.1 (0.03-0.2) | 1 (0-2) | 0.07 (0.02-0.14) | -1.16 (-2.03--0.29) |
| Montenegro | 0 (0-1) | 0.1 (0.03-0.21) | 1 (0-2) | 0.16 (0.04-0.33) | 2.52 (1.97-3.07) |
| Morocco | 31 (9-62) | 0.44 (0.12-0.88) | 59 (15-123) | 0.35 (0.08-0.72) | 0.21 (-0.33-0.76) |
| Mozambique | 8 (2-15) | 0.28 (0.08-0.56) | 14 (4-30) | 0.27 (0.07-0.55) | -0.13 (-1.5-1.26) |
| Myanmar | 51 (15-98) | 0.44 (0.12-0.86) | 59 (17-117) | 0.23 (0.06-0.47) | -1.72 (-2.25--1.18) |
| Namibia | 1 (0-2) | 0.35 (0.09-0.7) | 2 (0-4) | 0.27 (0.07-0.54) | -0.83 (-1.9-0.25) |
| Nauru | 0 (0-0) | 0.34 (0.09-0.71) | 0 (0-0) | 0.34 (0.1-0.71) | 0.33 (-0.74-1.41) |
| Nepal | 12 (3-25) | 0.29 (0.08-0.57) | 14 (4-30) | 0.13 (0.03-0.27) | -2.16 (-3.04--1.27) |
| Netherlands | 10 (2-20) | 0.07 (0.02-0.15) | 7 (2-15) | 0.03 (0.01-0.06) | -2.63 (-3.66--1.58) |
| New Zealand | 2 (1-5) | 0.11 (0.03-0.21) | 2 (0-4) | 0.04 (0.01-0.08) | -2.83 (-3.57--2.09) |
| Nicaragua | 1 (0-1) | 0.09 (0.03-0.18) | 1 (0-2) | 0.05 (0.01-0.09) | -1.05 (-1.87--0.23) |
| Niger | 3 (1-7) | 0.25 (0.06-0.59) | 8 (2-19) | 0.23 (0.05-0.53) | -0.31 (-2.04-1.45) |
| Nigeria | 53 (14-117) | 0.27 (0.07-0.59) | 75 (21-152) | 0.19 (0.05-0.39) | -1.81 (-3.2--0.4) |
| Niue | 0 (0-0) | 0.2 (0.06-0.41) | 0 (0-0) | 0.15 (0.04-0.32) | -0.88 (-1.3--0.46) |
| North Macedonia | 6 (1-11) | 0.63 (0.17-1.23) | 8 (2-16) | 0.53 (0.13-1.09) | -0.2 (-0.51-0.11) |
| Northern Mariana Islands | 0 (0-0) | 0.16 (0.04-0.31) | 0 (0-0) | 0.09 (0.02-0.19) | 0.04 (-1.26-1.35) |
| Norway | 3 (1-6) | 0.07 (0.02-0.13) | 1 (0-2) | 0.01 (0-0.03) | -4.69 (-5.88--3.48) |
| Oman | 1 (0-2) | 0.35 (0.1-0.7) | 2 (0-3) | 0.18 (0.05-0.35) | -1.29 (-2.53--0.03) |
| Pakistan | 58 (18-113) | 0.25 (0.07-0.51) | 119 (37-240) | 0.23 (0.07-0.46) | -0.71 (-1.83-0.43) |
| Palau | 0 (0-0) | 0.21 (0.06-0.42) | 0 (0-0) | 0.19 (0.05-0.39) | 0.66 (0.14-1.19) |
| Palestine | 3 (1-5) | 0.58 (0.15-1.17) | 3 (1-6) | 0.29 (0.08-0.57) | -2.27 (-3.49--1.04) |
| Panama | 0 (0-1) | 0.06 (0.02-0.13) | 1 (0-1) | 0.03 (0.01-0.06) | -1.44 (-1.81--1.07) |
| Papua New Guinea | 2 (1-4) | 0.27 (0.07-0.54) | 4 (1-9) | 0.22 (0.06-0.45) | -0.69 (-2.03-0.68) |
| Paraguay | 1 (0-3) | 0.13 (0.03-0.27) | 2 (0-3) | 0.05 (0.01-0.11) | -2.52 (-3.08--1.96) |
| Peru | 5 (1-10) | 0.08 (0.02-0.17) | 8 (2-15) | 0.04 (0.01-0.09) | -1.84 (-2.4--1.28) |
| Philippines | 25 (7-47) | 0.21 (0.06-0.41) | 69 (19-138) | 0.17 (0.05-0.35) | 0.62 (-0.29-1.53) |
| Poland | 102 (27-200) | 0.39 (0.1-0.76) | 53 (13-107) | 0.11 (0.03-0.22) | -3.45 (-4.24--2.66) |
| Portugal | 26 (7-52) | 0.33 (0.08-0.65) | 10 (2-21) | 0.05 (0.01-0.1) | -5.23 (-6.33--4.12) |
| Puerto Rico | 1 (0-2) | 0.06 (0.02-0.11) | 1 (0-1) | 0.01 (0-0.03) | -3.25 (-4.02--2.47) |
| Qatar | 0 (0-0) | 0.2 (0.05-0.42) | 0 (0-0) | 0.06 (0.02-0.13) | -4.08 (-6.16--1.96) |
| Republic of Korea | 40 (11-80) | 0.28 (0.07-0.57) | 17 (4-36) | 0.03 (0.01-0.06) | -6.17 (-6.72--5.62) |
| Republic of Moldova | 6 (2-12) | 0.28 (0.07-0.54) | 6 (2-11) | 0.16 (0.04-0.31) | -0.56 (-1.21-0.1) |
| Romania | 61 (16-121) | 0.44 (0.12-0.87) | 54 (13-106) | 0.21 (0.05-0.41) | -1.65 (-2.48--0.81) |
| Russian Federation | 694 (189-1338) | 0.59 (0.16-1.13) | 445 (114-882) | 0.28 (0.07-0.54) | -2.96 (-3.77--2.14) |
| Rwanda | 7 (2-15) | 0.51 (0.15-1.04) | 6 (2-14) | 0.2 (0.05-0.44) | -3.76 (-5.06--2.43) |
| Saint Kitts and Nevis | 0 (0-0) | 0.38 (0.11-0.75) | 0 (0-0) | 0.15 (0.04-0.29) | -2.78 (-2.95--2.61) |
| Saint Lucia | 0 (0-0) | 0.42 (0.12-0.83) | 0 (0-0) | 0.15 (0.04-0.3) | -2.59 (-3.09--2.09) |
| Saint Vincent and the Grenadines | 0 (0-0) | 0.31 (0.08-0.59) | 0 (0-0) | 0.15 (0.04-0.29) | -1.44 (-1.76--1.12) |
| Samoa | 0 (0-0) | 0.19 (0.05-0.39) | 0 (0-0) | 0.16 (0.05-0.33) | -0.07 (-0.8-0.68) |
| San Marino | 0 (0-0) | 0.09 (0.02-0.18) | 0 (0-0) | 0.03 (0.01-0.07) | -2.7 (-4--1.38) |
| Sao Tome and Principe | 0 (0-0) | 0.25 (0.07-0.49) | 0 (0-0) | 0.28 (0.08-0.54) | -0.02 (-1-0.96) |
| Saudi Arabia | 12 (3-23) | 0.47 (0.13-0.93) | 27 (8-49) | 0.3 (0.09-0.59) | -0.92 (-2.08-0.25) |
| Senegal | 4 (1-7) | 0.25 (0.07-0.52) | 7 (2-15) | 0.2 (0.05-0.42) | -0.37 (-1.57-0.86) |
| Serbia | 32 (9-61) | 0.65 (0.18-1.24) | 33 (8-68) | 0.33 (0.08-0.66) | -1.57 (-2.29--0.84) |
| Seychelles | 0 (0-0) | 0.16 (0.04-0.31) | 0 (0-0) | 0.12 (0.03-0.23) | -0.02 (-0.2-0.16) |
| Sierra Leone | 3 (1-6) | 0.31 (0.08-0.67) | 6 (2-12) | 0.33 (0.08-0.71) | 0.2 (-0.99-1.4) |
| Singapore | 2 (1-4) | 0.19 (0.05-0.38) | 1 (0-2) | 0.02 (0-0.03) | -6.69 (-7.05--6.32) |
| Slovakia | 10 (2-20) | 0.28 (0.07-0.55) | 7 (2-14) | 0.11 (0.03-0.23) | -2.5 (-3.19--1.8) |
| Slovenia | 3 (1-7) | 0.22 (0.06-0.43) | 1 (0-3) | 0.04 (0.01-0.08) | -4.12 (-5.21--3.01) |
| Solomon Islands | 0 (0-0) | 0.3 (0.08-0.62) | 0 (0-1) | 0.27 (0.07-0.56) | 0.3 (-1.15-1.78) |
| Somalia | 3 (1-7) | 0.25 (0.06-0.6) | 6 (1-14) | 0.2 (0.04-0.47) | -0.93 (-2.61-0.77) |
| South Africa | 17 (5-35) | 0.15 (0.04-0.3) | 36 (10-69) | 0.15 (0.04-0.28) | 0.56 (-0.01-1.15) |
| South Sudan | 2 (1-5) | 0.22 (0.06-0.48) | 3 (1-6) | 0.19 (0.05-0.4) | -1.04 (-2.71-0.66) |
| Spain | 50 (12-100) | 0.15 (0.04-0.3) | 20 (4-42) | 0.02 (0.01-0.05) | -4.76 (-5.98--3.52) |
| Sri Lanka | 14 (4-26) | 0.32 (0.08-0.64) | 29 (8-62) | 0.21 (0.05-0.44) | 0.54 (0.03-1.05) |
| Sudan | 28 (7-60) | 0.62 (0.15-1.33) | 39 (10-84) | 0.42 (0.1-0.9) | -1.53 (-2.66--0.39) |
| Suriname | 0 (0-0) | 0.15 (0.04-0.29) | 0 (0-0) | 0.06 (0.01-0.11) | -2.46 (-2.91--2.02) |
| Sweden | 11 (3-22) | 0.1 (0.03-0.2) | 5 (1-10) | 0.03 (0.01-0.06) | -3.57 (-4.94--2.18) |
| Switzerland | 7 (2-15) | 0.1 (0.02-0.19) | 3 (1-7) | 0.02 (0-0.05) | -3.98 (-5.26--2.69) |
| Syrian Arab Republic | 10 (3-19) | 0.4 (0.12-0.79) | 14 (4-28) | 0.26 (0.07-0.53) | -1.06 (-2.34-0.23) |
| Taiwan (Province of China) | 4 (1-8) | 0.06 (0.02-0.12) | 2 (0-4) | 0.01 (0-0.02) | -4.62 (-5.15--4.08) |
| Tajikistan | 6 (2-10) | 0.37 (0.11-0.7) | 8 (2-16) | 0.29 (0.08-0.61) | -1.41 (-2.38--0.42) |
| Thailand | 30 (8-58) | 0.17 (0.05-0.34) | 49 (13-100) | 0.08 (0.02-0.17) | -1.34 (-1.81--0.87) |
| Timor-Leste | 0 (0-1) | 0.29 (0.08-0.58) | 1 (0-2) | 0.26 (0.07-0.56) | 0.95 (-0.26-2.18) |
| Togo | 2 (1-4) | 0.32 (0.09-0.7) | 5 (1-11) | 0.28 (0.07-0.6) | 0.14 (-1.26-1.56) |
| Tokelau | 0 (0-0) | 0.27 (0.07-0.56) | 0 (0-0) | 0.18 (0.04-0.36) | -0.84 (-1.05--0.62) |
| Tonga | 0 (0-0) | 0.11 (0.03-0.22) | 0 (0-0) | 0.1 (0.03-0.2) | 0.73 (0.18-1.29) |
| Trinidad and Tobago | 1 (0-1) | 0.13 (0.03-0.25) | 0 (0-1) | 0.05 (0.01-0.09) | -2.66 (-3.02--2.29) |
| Tunisia | 5 (1-10) | 0.22 (0.06-0.46) | 10 (2-23) | 0.16 (0.04-0.35) | 0 (-0.47-0.47) |
| Turkey | 50 (13-95) | 0.29 (0.07-0.58) | 64 (17-131) | 0.13 (0.04-0.27) | -1.27 (-1.67--0.87) |
| Turkmenistan | 5 (1-9) | 0.42 (0.13-0.82) | 10 (3-19) | 0.43 (0.13-0.87) | 0.45 (-0.17-1.09) |
| Tuvalu | 0 (0-0) | 0.31 (0.08-0.62) | 0 (0-0) | 0.23 (0.06-0.5) | -0.74 (-1.06--0.42) |
| Uganda | 6 (2-13) | 0.21 (0.06-0.45) | 10 (3-22) | 0.14 (0.04-0.31) | -2.19 (-3.76--0.6) |
| Ukraine | 228 (61-444) | 0.48 (0.13-0.94) | 120 (29-240) | 0.23 (0.06-0.46) | -2.77 (-3.61--1.91) |
| United Arab Emirates | 0 (0-1) | 0.26 (0.07-0.53) | 1 (0-3) | 0.42 (0.1-0.89) | 1.43 (-1.17-4.1) |
| United Kingdom | 85 (21-171) | 0.14 (0.03-0.27) | 26 (6-54) | 0.03 (0.01-0.06) | -4.95 (-6.08--3.8) |
| United Republic of Tanzania | 8 (2-17) | 0.17 (0.04-0.35) | 20 (5-42) | 0.17 (0.04-0.37) | 0.11 (-1.2-1.43) |
| United States of America | 77 (19-156) | 0.04 (0.01-0.07) | 59 (14-123) | 0.02 (0-0.03) | -2.91 (-3.81--2.01) |
| United States Virgin Islands | 0 (0-0) | 0.1 (0.03-0.2) | 0 (0-0) | 0.03 (0.01-0.05) | -2.47 (-3.03--1.9) |
| Uruguay | 5 (1-9) | 0.2 (0.05-0.39) | 2 (1-5) | 0.06 (0.01-0.12) | -3.51 (-4.33--2.69) |
| Uzbekistan | 20 (6-37) | 0.3 (0.09-0.56) | 30 (9-58) | 0.22 (0.06-0.44) | -1.3 (-1.93--0.68) |
| Vanuatu | 0 (0-0) | 0.25 (0.07-0.52) | 0 (0-0) | 0.2 (0.05-0.41) | -0.23 (-1.51-1.06) |
| Venezuela (Bolivarian Republic of) | 4 (1-7) | 0.07 (0.02-0.14) | 8 (2-16) | 0.05 (0.01-0.1) | -0.37 (-1.02-0.28) |
| Viet Nam | 66 (17-139) | 0.3 (0.08-0.63) | 122 (32-251) | 0.24 (0.06-0.5) | 0.46 (0.06-0.86) |
| Yemen | 16 (4-31) | 0.64 (0.17-1.28) | 37 (9-78) | 0.53 (0.12-1.14) | -0.68 (-1.98-0.63) |
| Zambia | 3 (1-7) | 0.27 (0.07-0.58) | 8 (2-16) | 0.26 (0.07-0.54) | -0.22 (-1.87-1.46) |

Abbreviations: IS,Ischemic stroke,IIPUFAs, insufficient intake of polyunsaturated fatty acids,EAPC, estimated annual percentage change, SDl, Sociodemographic Index; Ul,uncertainty interval. “ EAPC is expressed as 95% CIs.
