# Supplementary material for: Global burden of disease analysis and projections of ischemic stroke linked to inadequate polyunsaturated fatty acid intake in older women (1990–2021)
Source: Front Nutr. 2025 Dec 12;12:1659895. doi: 10.3389/fnut.2025.1659895 (PMC12742205; doi:10.3389/fnut.2025.1659895)
Supplement: Supplementary file 2 [file Table_2.docx]

Table S5. Sex-stratified global ischemic stroke burden attributable to diet low in PUFA by age group, 2021 (GBD 2021): absolute numbers (thousands) and crude rates (per 100,000) with 95% uncertainty intervals for Deaths, DALYs, YLLs, and YLDs, reported for women ≥50, men ≥50, and women <50. Abbreviations: DALY, disability-adjusted life year; YLL, years of life lost; YLD, years lived with disability.

| measure | location | sex | age years | metric | val | upper | lower |
| --- | --- | --- | --- | --- | --- | --- | --- |
| Deaths | Global | Male | 25-29 | Number | 0.64 | 1.11 | 0.26 |
| Deaths | Global | Female | 25-29 | Number | 0.51 | 0.88 | 0.20 |
| Deaths | Global | Both | 25-29 | Number | 1.15 | 1.95 | 0.45 |
| Deaths | Global | Male | 25-29 | Rate | 0.00 | 0.00 | 0.00 |
| Deaths | Global | Female | 25-29 | Rate | 0.00 | 0.00 | 0.00 |
| Deaths | Global | Both | 25-29 | Rate | 0.00 | 0.00 | 0.00 |
| Deaths | Global | Male | 30-34 | Number | 1.35 | 2.33 | 0.54 |
| Deaths | Global | Female | 30-34 | Number | 0.88 | 1.49 | 0.34 |
| Deaths | Global | Both | 30-34 | Number | 2.23 | 3.76 | 0.87 |
| Deaths | Global | Male | 30-34 | Rate | 0.00 | 0.00 | 0.00 |
| Deaths | Global | Female | 30-34 | Rate | 0.00 | 0.00 | 0.00 |
| Deaths | Global | Both | 30-34 | Rate | 0.00 | 0.00 | 0.00 |
| Deaths | Global | Male | 35-39 | Number | 2.29 | 3.94 | 0.91 |
| Deaths | Global | Female | 35-39 | Number | 1.45 | 2.50 | 0.56 |
| Deaths | Global | Both | 35-39 | Number | 3.73 | 6.13 | 1.48 |
| Deaths | Global | Male | 35-39 | Rate | 0.00 | 0.00 | 0.00 |
| Deaths | Global | Female | 35-39 | Rate | 0.00 | 0.00 | 0.00 |
| Deaths | Global | Both | 35-39 | Rate | 0.00 | 0.00 | 0.00 |
| Deaths | Global | Male | 40-44 | Number | 4.07 | 6.95 | 1.61 |
| Deaths | Global | Female | 40-44 | Number | 2.50 | 4.32 | 0.97 |
| Deaths | Global | Both | 40-44 | Number | 6.57 | 10.93 | 2.52 |
| Deaths | Global | Male | 40-44 | Rate | 0.00 | 0.00 | 0.00 |
| Deaths | Global | Female | 40-44 | Rate | 0.00 | 0.00 | 0.00 |
| Deaths | Global | Both | 40-44 | Rate | 0.00 | 0.00 | 0.00 |
| Deaths | Global | Male | 45-49 | Number | 8.25 | 14.23 | 3.18 |
| Deaths | Global | Female | 45-49 | Number | 4.54 | 7.71 | 1.71 |
| Deaths | Global | Both | 45-49 | Number | 12.79 | 21.55 | 4.85 |
| Deaths | Global | Male | 45-49 | Rate | 0.00 | 0.01 | 0.00 |
| Deaths | Global | Female | 45-49 | Rate | 0.00 | 0.00 | 0.00 |
| Deaths | Global | Both | 45-49 | Rate | 0.00 | 0.00 | 0.00 |
| Deaths | Global | Male | 50-54 | Number | 15.91 | 28.19 | 5.63 |
| Deaths | Global | Female | 50-54 | Number | 8.19 | 14.37 | 2.96 |
| Deaths | Global | Both | 50-54 | Number | 24.10 | 41.52 | 8.63 |
| Deaths | Global | Male | 50-54 | Rate | 0.01 | 0.01 | 0.00 |
| Deaths | Global | Female | 50-54 | Rate | 0.00 | 0.01 | 0.00 |
| Deaths | Global | Both | 50-54 | Rate | 0.01 | 0.01 | 0.00 |
| Deaths | Global | Male | 55-59 | Number | 23.06 | 41.44 | 7.64 |
| Deaths | Global | Female | 55-59 | Number | 12.96 | 23.33 | 4.42 |
| Deaths | Global | Both | 55-59 | Number | 36.02 | 63.83 | 12.05 |
| Deaths | Global | Male | 55-59 | Rate | 0.01 | 0.02 | 0.00 |
| Deaths | Global | Female | 55-59 | Rate | 0.01 | 0.01 | 0.00 |
| Deaths | Global | Both | 55-59 | Rate | 0.01 | 0.02 | 0.00 |
| Deaths | Global | Male | 60-64 | Number | 40.04 | 74.54 | 12.01 |
| Deaths | Global | Female | 60-64 | Number | 22.28 | 41.19 | 6.90 |
| Deaths | Global | Both | 60-64 | Number | 62.32 | 115.18 | 19.04 |
| Deaths | Global | Male | 60-64 | Rate | 0.03 | 0.05 | 0.01 |
| Deaths | Global | Female | 60-64 | Rate | 0.01 | 0.03 | 0.00 |
| Deaths | Global | Both | 60-64 | Rate | 0.02 | 0.04 | 0.01 |
| Deaths | Global | Male | 65-69 | Number | 50.12 | 97.34 | 13.39 |
| Deaths | Global | Female | 65-69 | Number | 31.80 | 61.35 | 9.20 |
| Deaths | Global | Both | 65-69 | Number | 81.92 | 156.62 | 22.76 |
| Deaths | Global | Male | 65-69 | Rate | 0.04 | 0.07 | 0.01 |
| Deaths | Global | Female | 65-69 | Rate | 0.02 | 0.04 | 0.01 |
| Deaths | Global | Both | 65-69 | Rate | 0.03 | 0.06 | 0.01 |
| Deaths | Global | Male | 70-74 | Number | 61.70 | 121.85 | 15.69 |
| Deaths | Global | Female | 70-74 | Number | 47.95 | 95.93 | 12.60 |
| Deaths | Global | Both | 70-74 | Number | 109.65 | 215.09 | 28.78 |
| Deaths | Global | Male | 70-74 | Rate | 0.06 | 0.13 | 0.02 |
| Deaths | Global | Female | 70-74 | Rate | 0.04 | 0.09 | 0.01 |
| Deaths | Global | Both | 70-74 | Rate | 0.05 | 0.10 | 0.01 |
| Deaths | Global | Male | 75-79 | Number | 55.47 | 115.70 | 13.68 |
| Deaths | Global | Female | 75-79 | Number | 50.22 | 103.52 | 12.74 |
| Deaths | Global | Both | 75-79 | Number | 105.69 | 210.21 | 26.40 |
| Deaths | Global | Male | 75-79 | Rate | 0.09 | 0.19 | 0.02 |
| Deaths | Global | Female | 75-79 | Rate | 0.07 | 0.14 | 0.02 |
| Deaths | Global | Both | 75-79 | Rate | 0.08 | 0.16 | 0.02 |
| Deaths | Global | Male | 80-84 | Number | 59.11 | 126.24 | 14.53 |
| Deaths | Global | Female | 80-84 | Number | 68.54 | 145.33 | 15.28 |
| Deaths | Global | Both | 80-84 | Number | 127.65 | 269.81 | 29.46 |
| Deaths | Global | Male | 80-84 | Rate | 0.16 | 0.34 | 0.04 |
| Deaths | Global | Female | 80-84 | Rate | 0.13 | 0.29 | 0.03 |
| Deaths | Global | Both | 80-84 | Rate | 0.15 | 0.31 | 0.03 |
| Deaths | Global | Male | 85-89 | Number | 48.33 | 101.13 | 11.30 |
| Deaths | Global | Female | 85-89 | Number | 59.58 | 128.82 | 13.13 |
| Deaths | Global | Both | 85-89 | Number | 107.92 | 230.70 | 23.99 |
| Deaths | Global | Male | 85-89 | Rate | 0.28 | 0.59 | 0.07 |
| Deaths | Global | Female | 85-89 | Rate | 0.21 | 0.45 | 0.05 |
| Deaths | Global | Both | 85-89 | Rate | 0.24 | 0.50 | 0.05 |
| Deaths | Global | Male | 90-94 | Number | 21.23 | 44.42 | 5.01 |
| Deaths | Global | Female | 90-94 | Number | 37.01 | 79.82 | 7.91 |
| Deaths | Global | Both | 90-94 | Number | 58.24 | 125.37 | 12.71 |
| Deaths | Global | Male | 90-94 | Rate | 0.36 | 0.76 | 0.09 |
| Deaths | Global | Female | 90-94 | Rate | 0.31 | 0.66 | 0.07 |
| Deaths | Global | Both | 90-94 | Rate | 0.33 | 0.70 | 0.07 |
| Deaths | Global | Male | 95+ | Number | 5.15 | 10.66 | 1.13 |
| Deaths | Global | Female | 95+ | Number | 16.03 | 34.38 | 3.37 |
| Deaths | Global | Both | 95+ | Number | 21.17 | 44.89 | 4.49 |
| Deaths | Global | Male | 95+ | Rate | 0.34 | 0.70 | 0.08 |
| Deaths | Global | Female | 95+ | Rate | 0.41 | 0.87 | 0.09 |
| Deaths | Global | Both | 95+ | Rate | 0.39 | 0.82 | 0.08 |
| DALYs | Global | Male | 25-29 | Number | 77.28 | 137.77 | 30.15 |
| DALYs | Global | Female | 25-29 | Number | 86.33 | 149.33 | 33.70 |
| DALYs | Global | Both | 25-29 | Number | 163.61 | 283.98 | 63.70 |
| DALYs | Global | Male | 25-29 | Rate | 0.03 | 0.05 | 0.01 |
| DALYs | Global | Female | 25-29 | Rate | 0.03 | 0.05 | 0.01 |
| DALYs | Global | Both | 25-29 | Rate | 0.03 | 0.05 | 0.01 |
| DALYs | Global | Male | 30-34 | Number | 129.52 | 226.38 | 51.29 |
| DALYs | Global | Female | 30-34 | Number | 123.83 | 216.88 | 47.62 |
| DALYs | Global | Both | 30-34 | Number | 253.35 | 450.80 | 99.58 |
| DALYs | Global | Male | 30-34 | Rate | 0.04 | 0.07 | 0.02 |
| DALYs | Global | Female | 30-34 | Rate | 0.04 | 0.07 | 0.02 |
| DALYs | Global | Both | 30-34 | Rate | 0.04 | 0.07 | 0.02 |
| DALYs | Global | Male | 35-39 | Number | 185.52 | 324.65 | 73.65 |
| DALYs | Global | Female | 35-39 | Number | 163.72 | 285.29 | 62.56 |
| DALYs | Global | Both | 35-39 | Number | 349.25 | 604.72 | 138.40 |
| DALYs | Global | Male | 35-39 | Rate | 0.07 | 0.11 | 0.03 |
| DALYs | Global | Female | 35-39 | Rate | 0.06 | 0.10 | 0.02 |
| DALYs | Global | Both | 35-39 | Rate | 0.06 | 0.11 | 0.02 |
| DALYs | Global | Male | 40-44 | Number | 274.14 | 478.15 | 107.09 |
| DALYs | Global | Female | 40-44 | Number | 219.40 | 382.66 | 83.40 |
| DALYs | Global | Both | 40-44 | Number | 493.54 | 852.15 | 190.56 |
| DALYs | Global | Male | 40-44 | Rate | 0.11 | 0.19 | 0.04 |
| DALYs | Global | Female | 40-44 | Rate | 0.09 | 0.15 | 0.03 |
| DALYs | Global | Both | 40-44 | Rate | 0.10 | 0.17 | 0.04 |
| DALYs | Global | Male | 45-49 | Number | 466.21 | 818.71 | 175.60 |
| DALYs | Global | Female | 45-49 | Number | 322.74 | 561.09 | 122.84 |
| DALYs | Global | Both | 45-49 | Number | 788.95 | 1377.46 | 292.96 |
| DALYs | Global | Male | 45-49 | Rate | 0.20 | 0.34 | 0.07 |
| DALYs | Global | Female | 45-49 | Rate | 0.14 | 0.24 | 0.05 |
| DALYs | Global | Both | 45-49 | Rate | 0.17 | 0.29 | 0.06 |
| DALYs | Global | Male | 50-54 | Number | 762.26 | 1357.76 | 267.33 |
| DALYs | Global | Female | 50-54 | Number | 475.03 | 846.39 | 169.44 |
| DALYs | Global | Both | 50-54 | Number | 1237.30 | 2161.39 | 441.62 |
| DALYs | Global | Male | 50-54 | Rate | 0.34 | 0.61 | 0.12 |
| DALYs | Global | Female | 50-54 | Rate | 0.21 | 0.38 | 0.08 |
| DALYs | Global | Both | 50-54 | Rate | 0.28 | 0.49 | 0.10 |
| DALYs | Global | Male | 55-59 | Number | 959.95 | 1757.70 | 320.70 |
| DALYs | Global | Female | 55-59 | Number | 623.84 | 1123.63 | 209.96 |
| DALYs | Global | Both | 55-59 | Number | 1583.78 | 2836.05 | 534.05 |
| DALYs | Global | Male | 55-59 | Rate | 0.49 | 0.90 | 0.16 |
| DALYs | Global | Female | 55-59 | Rate | 0.31 | 0.56 | 0.10 |
| DALYs | Global | Both | 55-59 | Rate | 0.40 | 0.72 | 0.13 |
| DALYs | Global | Male | 60-64 | Number | 1339.39 | 2492.65 | 401.01 |
| DALYs | Global | Female | 60-64 | Number | 823.58 | 1523.10 | 257.75 |
| DALYs | Global | Both | 60-64 | Number | 2162.97 | 3995.26 | 659.72 |
| DALYs | Global | Male | 60-64 | Rate | 0.86 | 1.60 | 0.26 |
| DALYs | Global | Female | 60-64 | Rate | 0.50 | 0.93 | 0.16 |
| DALYs | Global | Both | 60-64 | Rate | 0.68 | 1.25 | 0.21 |
| DALYs | Global | Male | 65-69 | Number | 1409.55 | 2726.41 | 385.55 |
| DALYs | Global | Female | 65-69 | Number | 962.25 | 1875.27 | 275.17 |
| DALYs | Global | Both | 65-69 | Number | 2371.80 | 4537.52 | 670.60 |
| DALYs | Global | Male | 65-69 | Rate | 1.07 | 2.07 | 0.29 |
| DALYs | Global | Female | 65-69 | Rate | 0.67 | 1.30 | 0.19 |
| DALYs | Global | Both | 65-69 | Rate | 0.86 | 1.64 | 0.24 |
| DALYs | Global | Male | 70-74 | Number | 1396.13 | 2744.48 | 352.80 |
| DALYs | Global | Female | 70-74 | Number | 1117.95 | 2218.47 | 294.33 |
| DALYs | Global | Both | 70-74 | Number | 2514.08 | 4893.10 | 659.46 |
| DALYs | Global | Male | 70-74 | Rate | 1.45 | 2.85 | 0.37 |
| DALYs | Global | Female | 70-74 | Rate | 1.02 | 2.03 | 0.27 |
| DALYs | Global | Both | 70-74 | Rate | 1.22 | 2.38 | 0.32 |
| DALYs | Global | Male | 75-79 | Number | 1008.96 | 2087.42 | 256.48 |
| DALYs | Global | Female | 75-79 | Number | 918.03 | 1902.08 | 230.74 |
| DALYs | Global | Both | 75-79 | Number | 1926.99 | 3849.63 | 496.16 |
| DALYs | Global | Male | 75-79 | Rate | 1.69 | 3.49 | 0.43 |
| DALYs | Global | Female | 75-79 | Rate | 1.27 | 2.64 | 0.32 |
| DALYs | Global | Both | 75-79 | Rate | 1.46 | 2.92 | 0.38 |
| DALYs | Global | Male | 80-84 | Number | 826.37 | 1766.87 | 200.73 |
| DALYs | Global | Female | 80-84 | Number | 948.19 | 1999.73 | 208.18 |
| DALYs | Global | Both | 80-84 | Number | 1774.55 | 3774.44 | 405.04 |
| DALYs | Global | Male | 80-84 | Rate | 2.25 | 4.82 | 0.55 |
| DALYs | Global | Female | 80-84 | Rate | 1.86 | 3.93 | 0.41 |
| DALYs | Global | Both | 80-84 | Rate | 2.03 | 4.31 | 0.46 |
| DALYs | Global | Male | 85-89 | Number | 519.39 | 1085.35 | 121.67 |
| DALYs | Global | Female | 85-89 | Number | 648.93 | 1391.78 | 142.67 |
| DALYs | Global | Both | 85-89 | Number | 1168.33 | 2500.89 | 257.94 |
| DALYs | Global | Male | 85-89 | Rate | 3.01 | 6.29 | 0.71 |
| DALYs | Global | Female | 85-89 | Rate | 2.28 | 4.89 | 0.50 |
| DALYs | Global | Both | 85-89 | Rate | 2.56 | 5.47 | 0.56 |
| DALYs | Global | Male | 90-94 | Number | 195.40 | 411.43 | 45.75 |
| DALYs | Global | Female | 90-94 | Number | 344.89 | 745.71 | 72.89 |
| DALYs | Global | Both | 90-94 | Number | 540.29 | 1152.76 | 117.22 |
| DALYs | Global | Male | 90-94 | Rate | 3.35 | 7.06 | 0.78 |
| DALYs | Global | Female | 90-94 | Rate | 2.86 | 6.18 | 0.60 |
| DALYs | Global | Both | 90-94 | Rate | 3.02 | 6.44 | 0.66 |
| DALYs | Global | Male | 95+ | Number | 44.94 | 92.72 | 9.86 |
| DALYs | Global | Female | 95+ | Number | 138.36 | 291.93 | 29.02 |
| DALYs | Global | Both | 95+ | Number | 183.30 | 382.71 | 38.66 |
| DALYs | Global | Male | 95+ | Rate | 2.97 | 6.13 | 0.65 |
| DALYs | Global | Female | 95+ | Rate | 3.51 | 7.41 | 0.74 |
| DALYs | Global | Both | 95+ | Rate | 3.36 | 7.02 | 0.71 |
| YLDs | Global | Male | 95+ | Number | 3.14 | 7.09 | 0.68 |
| YLDs | Global | Female | 95+ | Number | 8.89 | 19.89 | 1.90 |
| YLDs | Global | Both | 95+ | Number | 12.03 | 27.09 | 2.58 |
| YLDs | Global | Male | 95+ | Rate | 0.21 | 0.47 | 0.05 |
| YLDs | Global | Female | 95+ | Rate | 0.23 | 0.51 | 0.05 |
| YLDs | Global | Both | 95+ | Rate | 0.22 | 0.50 | 0.05 |
| YLDs | Global | Male | 25-29 | Number | 37.12 | 68.72 | 13.63 |
| YLDs | Global | Female | 25-29 | Number | 54.49 | 101.81 | 20.32 |
| YLDs | Global | Both | 25-29 | Number | 91.61 | 171.77 | 33.69 |
| YLDs | Global | Male | 25-29 | Rate | 0.01 | 0.02 | 0.00 |
| YLDs | Global | Female | 25-29 | Rate | 0.02 | 0.03 | 0.01 |
| YLDs | Global | Both | 25-29 | Rate | 0.02 | 0.03 | 0.01 |
| YLDs | Global | Male | 30-34 | Number | 51.50 | 91.71 | 19.83 |
| YLDs | Global | Female | 30-34 | Number | 73.18 | 133.07 | 27.55 |
| YLDs | Global | Both | 30-34 | Number | 124.68 | 224.67 | 47.38 |
| YLDs | Global | Male | 30-34 | Rate | 0.02 | 0.03 | 0.01 |
| YLDs | Global | Female | 30-34 | Rate | 0.02 | 0.04 | 0.01 |
| YLDs | Global | Both | 30-34 | Rate | 0.02 | 0.04 | 0.01 |
| YLDs | Global | Male | 35-39 | Number | 64.73 | 116.39 | 23.99 |
| YLDs | Global | Female | 35-39 | Number | 87.36 | 155.91 | 32.40 |
| YLDs | Global | Both | 35-39 | Number | 152.09 | 272.30 | 56.50 |
| YLDs | Global | Male | 35-39 | Rate | 0.02 | 0.04 | 0.01 |
| YLDs | Global | Female | 35-39 | Rate | 0.03 | 0.06 | 0.01 |
| YLDs | Global | Both | 35-39 | Rate | 0.03 | 0.05 | 0.01 |
| YLDs | Global | Male | 40-44 | Number | 79.36 | 143.66 | 28.93 |
| YLDs | Global | Female | 40-44 | Number | 99.86 | 179.04 | 37.38 |
| YLDs | Global | Both | 40-44 | Number | 179.23 | 322.45 | 66.68 |
| YLDs | Global | Male | 40-44 | Rate | 0.03 | 0.06 | 0.01 |
| YLDs | Global | Female | 40-44 | Rate | 0.04 | 0.07 | 0.02 |
| YLDs | Global | Both | 40-44 | Rate | 0.04 | 0.06 | 0.01 |
| YLDs | Global | Male | 45-49 | Number | 111.95 | 206.21 | 38.83 |
| YLDs | Global | Female | 45-49 | Number | 127.85 | 231.52 | 46.31 |
| YLDs | Global | Both | 45-49 | Number | 239.80 | 437.60 | 85.45 |
| YLDs | Global | Male | 45-49 | Rate | 0.05 | 0.09 | 0.02 |
| YLDs | Global | Female | 45-49 | Rate | 0.05 | 0.10 | 0.02 |
| YLDs | Global | Both | 45-49 | Rate | 0.05 | 0.09 | 0.02 |
| YLDs | Global | Male | 50-54 | Number | 154.49 | 295.88 | 51.48 |
| YLDs | Global | Female | 50-54 | Number | 162.52 | 307.00 | 54.44 |
| YLDs | Global | Both | 50-54 | Number | 317.00 | 604.51 | 105.93 |
| YLDs | Global | Male | 50-54 | Rate | 0.07 | 0.13 | 0.02 |
| YLDs | Global | Female | 50-54 | Rate | 0.07 | 0.14 | 0.02 |
| YLDs | Global | Both | 50-54 | Rate | 0.07 | 0.14 | 0.02 |
| YLDs | Global | Male | 55-59 | Number | 186.66 | 365.84 | 56.75 |
| YLDs | Global | Female | 55-59 | Number | 189.43 | 368.96 | 58.70 |
| YLDs | Global | Both | 55-59 | Number | 376.09 | 734.77 | 114.85 |
| YLDs | Global | Male | 55-59 | Rate | 0.10 | 0.19 | 0.03 |
| YLDs | Global | Female | 55-59 | Rate | 0.09 | 0.18 | 0.03 |
| YLDs | Global | Both | 55-59 | Rate | 0.10 | 0.19 | 0.03 |
| YLDs | Global | Male | 60-64 | Number | 183.22 | 355.37 | 51.38 |
| YLDs | Global | Female | 60-64 | Number | 180.89 | 351.03 | 50.65 |
| YLDs | Global | Both | 60-64 | Number | 364.11 | 702.55 | 102.03 |
| YLDs | Global | Male | 60-64 | Rate | 0.12 | 0.23 | 0.03 |
| YLDs | Global | Female | 60-64 | Rate | 0.11 | 0.21 | 0.03 |
| YLDs | Global | Both | 60-64 | Rate | 0.11 | 0.22 | 0.03 |
| YLDs | Global | Male | 65-69 | Number | 190.33 | 380.55 | 51.74 |
| YLDs | Global | Female | 65-69 | Number | 190.14 | 376.28 | 50.85 |
| YLDs | Global | Both | 65-69 | Number | 380.47 | 756.76 | 102.34 |
| YLDs | Global | Male | 65-69 | Rate | 0.14 | 0.29 | 0.04 |
| YLDs | Global | Female | 65-69 | Rate | 0.13 | 0.26 | 0.04 |
| YLDs | Global | Both | 65-69 | Rate | 0.14 | 0.27 | 0.04 |
| YLDs | Global | Male | 70-74 | Number | 159.95 | 329.73 | 42.57 |
| YLDs | Global | Female | 70-74 | Number | 158.38 | 325.59 | 40.65 |
| YLDs | Global | Both | 70-74 | Number | 318.32 | 652.02 | 83.00 |
| YLDs | Global | Male | 70-74 | Rate | 0.17 | 0.34 | 0.04 |
| YLDs | Global | Female | 70-74 | Rate | 0.14 | 0.30 | 0.04 |
| YLDs | Global | Both | 70-74 | Rate | 0.15 | 0.32 | 0.04 |
| YLDs | Global | Male | 75-79 | Number | 121.58 | 255.39 | 30.17 |
| YLDs | Global | Female | 75-79 | Number | 117.80 | 245.32 | 28.04 |
| YLDs | Global | Both | 75-79 | Number | 239.38 | 500.67 | 58.48 |
| YLDs | Global | Male | 75-79 | Rate | 0.20 | 0.43 | 0.05 |
| YLDs | Global | Female | 75-79 | Rate | 0.16 | 0.34 | 0.04 |
| YLDs | Global | Both | 75-79 | Rate | 0.18 | 0.38 | 0.04 |
| YLDs | Global | Male | 80-84 | Number | 87.98 | 188.69 | 19.41 |
| YLDs | Global | Female | 80-84 | Number | 94.69 | 205.93 | 20.88 |
| YLDs | Global | Both | 80-84 | Number | 182.67 | 390.28 | 40.56 |
| YLDs | Global | Male | 80-84 | Rate | 0.24 | 0.51 | 0.05 |
| YLDs | Global | Female | 80-84 | Rate | 0.19 | 0.40 | 0.04 |
| YLDs | Global | Both | 80-84 | Rate | 0.21 | 0.45 | 0.05 |
| YLDs | Global | Male | 85-89 | Number | 40.14 | 88.34 | 9.07 |
| YLDs | Global | Female | 85-89 | Number | 59.01 | 128.51 | 12.25 |
| YLDs | Global | Both | 85-89 | Number | 99.15 | 216.67 | 21.32 |
| YLDs | Global | Male | 85-89 | Rate | 0.23 | 0.51 | 0.05 |
| YLDs | Global | Female | 85-89 | Rate | 0.21 | 0.45 | 0.04 |
| YLDs | Global | Both | 85-89 | Rate | 0.22 | 0.47 | 0.05 |
| YLDs | Global | Male | 90-94 | Number | 12.14 | 26.83 | 2.73 |
| YLDs | Global | Female | 90-94 | Number | 26.15 | 57.76 | 5.63 |
| YLDs | Global | Both | 90-94 | Number | 38.29 | 84.37 | 8.35 |
| YLDs | Global | Male | 90-94 | Rate | 0.21 | 0.46 | 0.05 |
| YLDs | Global | Female | 90-94 | Rate | 0.22 | 0.48 | 0.05 |
| YLDs | Global | Both | 90-94 | Rate | 0.21 | 0.47 | 0.05 |
| YLLs | Global | Male | 95+ | Number | 41.79 | 86.50 | 9.22 |
| YLLs | Global | Female | 95+ | Number | 129.48 | 277.67 | 27.27 |
| YLLs | Global | Both | 95+ | Number | 171.27 | 363.03 | 36.31 |
| YLLs | Global | Male | 95+ | Rate | 2.76 | 5.72 | 0.61 |
| YLLs | Global | Female | 95+ | Rate | 3.29 | 7.05 | 0.69 |
| YLLs | Global | Both | 95+ | Rate | 3.14 | 6.66 | 0.67 |
| YLLs | Global | Male | 25-29 | Number | 40.16 | 69.78 | 16.19 |
| YLLs | Global | Female | 25-29 | Number | 31.84 | 55.01 | 12.65 |
| YLLs | Global | Both | 25-29 | Number | 72.00 | 122.46 | 28.03 |
| YLLs | Global | Male | 25-29 | Rate | 0.01 | 0.02 | 0.01 |
| YLLs | Global | Female | 25-29 | Rate | 0.01 | 0.02 | 0.00 |
| YLLs | Global | Both | 25-29 | Rate | 0.01 | 0.02 | 0.00 |
| YLLs | Global | Male | 30-34 | Number | 78.02 | 134.57 | 31.30 |
| YLLs | Global | Female | 30-34 | Number | 50.65 | 86.23 | 19.59 |
| YLLs | Global | Both | 30-34 | Number | 128.67 | 216.75 | 50.38 |
| YLLs | Global | Male | 30-34 | Rate | 0.03 | 0.04 | 0.01 |
| YLLs | Global | Female | 30-34 | Rate | 0.02 | 0.03 | 0.01 |
| YLLs | Global | Both | 30-34 | Rate | 0.02 | 0.04 | 0.01 |
| YLLs | Global | Male | 35-39 | Number | 120.80 | 208.12 | 48.13 |
| YLLs | Global | Female | 35-39 | Number | 76.36 | 131.71 | 29.62 |
| YLLs | Global | Both | 35-39 | Number | 197.16 | 323.61 | 77.93 |
| YLLs | Global | Male | 35-39 | Rate | 0.04 | 0.07 | 0.02 |
| YLLs | Global | Female | 35-39 | Rate | 0.03 | 0.05 | 0.01 |
| YLLs | Global | Both | 35-39 | Rate | 0.04 | 0.06 | 0.01 |
| YLLs | Global | Male | 40-44 | Number | 194.78 | 332.44 | 76.82 |
| YLLs | Global | Female | 40-44 | Number | 119.54 | 206.81 | 46.53 |
| YLLs | Global | Both | 40-44 | Number | 314.32 | 522.95 | 120.56 |
| YLLs | Global | Male | 40-44 | Rate | 0.08 | 0.13 | 0.03 |
| YLLs | Global | Female | 40-44 | Rate | 0.05 | 0.08 | 0.02 |
| YLLs | Global | Both | 40-44 | Rate | 0.06 | 0.10 | 0.02 |
| YLLs | Global | Male | 45-49 | Number | 354.26 | 610.97 | 136.74 |
| YLLs | Global | Female | 45-49 | Number | 194.89 | 330.99 | 73.52 |
| YLLs | Global | Both | 45-49 | Number | 549.15 | 925.37 | 208.46 |
| YLLs | Global | Male | 45-49 | Rate | 0.15 | 0.26 | 0.06 |
| YLLs | Global | Female | 45-49 | Rate | 0.08 | 0.14 | 0.03 |
| YLLs | Global | Both | 45-49 | Rate | 0.12 | 0.20 | 0.04 |
| YLLs | Global | Male | 50-54 | Number | 607.78 | 1076.40 | 214.89 |
| YLLs | Global | Female | 50-54 | Number | 312.52 | 548.30 | 112.94 |
| YLLs | Global | Both | 50-54 | Number | 920.29 | 1585.34 | 329.48 |
| YLLs | Global | Male | 50-54 | Rate | 0.27 | 0.48 | 0.10 |
| YLLs | Global | Female | 50-54 | Rate | 0.14 | 0.25 | 0.05 |
| YLLs | Global | Both | 50-54 | Rate | 0.21 | 0.36 | 0.07 |
| YLLs | Global | Male | 55-59 | Number | 773.28 | 1389.38 | 256.22 |
| YLLs | Global | Female | 55-59 | Number | 434.41 | 782.03 | 148.15 |
| YLLs | Global | Both | 55-59 | Number | 1207.69 | 2139.89 | 403.84 |
| YLLs | Global | Male | 55-59 | Rate | 0.40 | 0.71 | 0.13 |
| YLLs | Global | Female | 55-59 | Rate | 0.22 | 0.39 | 0.07 |
| YLLs | Global | Both | 55-59 | Rate | 0.31 | 0.54 | 0.10 |
| YLLs | Global | Male | 60-64 | Number | 1156.17 | 2152.36 | 346.74 |
| YLLs | Global | Female | 60-64 | Number | 642.69 | 1188.10 | 198.97 |
| YLLs | Global | Both | 60-64 | Number | 1798.86 | 3325.16 | 549.64 |
| YLLs | Global | Male | 60-64 | Rate | 0.74 | 1.38 | 0.22 |
| YLLs | Global | Female | 60-64 | Rate | 0.39 | 0.72 | 0.12 |
| YLLs | Global | Both | 60-64 | Rate | 0.56 | 1.04 | 0.17 |
| YLLs | Global | Male | 65-69 | Number | 1219.22 | 2367.96 | 325.67 |
| YLLs | Global | Female | 65-69 | Number | 772.12 | 1489.62 | 223.42 |
| YLLs | Global | Both | 65-69 | Number | 1991.33 | 3807.00 | 553.27 |
| YLLs | Global | Male | 65-69 | Rate | 0.92 | 1.80 | 0.25 |
| YLLs | Global | Female | 65-69 | Rate | 0.54 | 1.03 | 0.16 |
| YLLs | Global | Both | 65-69 | Rate | 0.72 | 1.38 | 0.20 |
| YLLs | Global | Male | 70-74 | Number | 1236.18 | 2441.36 | 314.39 |
| YLLs | Global | Female | 70-74 | Number | 959.57 | 1919.83 | 252.22 |
| YLLs | Global | Both | 70-74 | Number | 2195.75 | 4307.20 | 576.33 |
| YLLs | Global | Male | 70-74 | Rate | 1.28 | 2.53 | 0.33 |
| YLLs | Global | Female | 70-74 | Rate | 0.88 | 1.75 | 0.23 |
| YLLs | Global | Both | 70-74 | Rate | 1.07 | 2.09 | 0.28 |
| YLLs | Global | Male | 75-79 | Number | 887.38 | 1850.80 | 218.86 |
| YLLs | Global | Female | 75-79 | Number | 800.24 | 1649.37 | 203.08 |
| YLLs | Global | Both | 75-79 | Number | 1687.61 | 3356.30 | 421.56 |
| YLLs | Global | Male | 75-79 | Rate | 1.48 | 3.10 | 0.37 |
| YLLs | Global | Female | 75-79 | Rate | 1.11 | 2.29 | 0.28 |
| YLLs | Global | Both | 75-79 | Rate | 1.28 | 2.54 | 0.32 |
| YLLs | Global | Male | 80-84 | Number | 738.38 | 1576.98 | 181.50 |
| YLLs | Global | Female | 80-84 | Number | 853.50 | 1809.65 | 190.25 |
| YLLs | Global | Both | 80-84 | Number | 1591.88 | 3364.52 | 367.47 |
| YLLs | Global | Male | 80-84 | Rate | 2.01 | 4.30 | 0.50 |
| YLLs | Global | Female | 80-84 | Rate | 1.68 | 3.55 | 0.37 |
| YLLs | Global | Both | 80-84 | Rate | 1.82 | 3.84 | 0.42 |
| YLLs | Global | Male | 85-89 | Number | 479.25 | 1002.75 | 112.10 |
| YLLs | Global | Female | 85-89 | Number | 589.93 | 1275.42 | 130.04 |
| YLLs | Global | Both | 85-89 | Number | 1069.18 | 2285.60 | 237.72 |
| YLLs | Global | Male | 85-89 | Rate | 2.78 | 5.81 | 0.65 |
| YLLs | Global | Female | 85-89 | Rate | 2.07 | 4.48 | 0.46 |
| YLLs | Global | Both | 85-89 | Rate | 2.34 | 5.00 | 0.52 |
| YLLs | Global | Male | 90-94 | Number | 183.26 | 383.44 | 43.26 |
| YLLs | Global | Female | 90-94 | Number | 318.74 | 687.45 | 68.09 |
| YLLs | Global | Both | 90-94 | Number | 502.00 | 1080.74 | 109.61 |
| YLLs | Global | Male | 90-94 | Rate | 3.14 | 6.58 | 0.74 |
| YLLs | Global | Female | 90-94 | Rate | 2.64 | 5.70 | 0.56 |
| YLLs | Global | Both | 90-94 | Rate | 2.81 | 6.04 | 0.61 |
