# Supplementary material for: Global burden of disease analysis and projections of ischemic stroke linked to inadequate polyunsaturated fatty acid intake in older women (1990–2021)
Source: Front Nutr. 2025 Dec 12;12:1659895. doi: 10.3389/fnut.2025.1659895 (PMC12742205; doi:10.3389/fnut.2025.1659895)
Supplement: Supplementary file 3 [file Table_3.docx]

**Supplementary Table S6. Percent contributions to the 1990–2021 change attributable to diet low in polyunsaturated fatty acid — Global–Female (all outcomes) and YLDs by SDI.** Percent contributions may be negative or exceed 100% because they are directional components whose sum equals the observed percent change (three-factor Das Gupta decomposition). SDI, Sociodemographic Index; DALYs, disability-adjusted life years; YLDs, years lived with disability; YLLs, years of life lost.

| Group | Outcome | Population growth (%) | Population aging (%) | Epidemiological change (%) |
| --- | --- | --- | --- | --- |
| Global – Female | Deaths | 296.08 | 46.71 | -242.78 |
| Global – Female | DALYs | 289.45 | 20.01 | -209.46 |
| Global – Female | YLLs | 383.31 | 29.59 | -312.9 |
| Global – Female | YLDs | 91.24 | 4.46 | 4.31 |
| High-middle SDI | YLDs | 79.83 | -8.56 | 28.74 |
| Low-middle SDI | YLDs | 132.44 | 4.25 | -36.7 |
| High SDI | YLDs | -392.78 | 35.33 | 457.45 |
| Low SDI | YLDs | 99.63 | 0.6 | -0.22 |
| Middle SDI | YLDs | 88.21 | 4.75 | 7.04 |
