# Supplementary material for: Global burden of disease analysis and projections of ischemic stroke linked to inadequate polyunsaturated fatty acid intake in older women (1990–2021)
Source: Front Nutr. 2025 Dec 12;12:1659895. doi: 10.3389/fnut.2025.1659895 (PMC12742205; doi:10.3389/fnut.2025.1659895)
Supplement: Supplementary file 7 [file Data_Sheet_1.pdf]

Supplementary Methods — Section S1: Projection model diagnostics and selection

We implemented univariate ARIMA(p,d,q) and, where indicated, parsimonious EC models. Stationarity was assessed by ADF and KPSS; the differencing order (d) was set accordingly. Candidate ARIMA models were fit over a grid of (p,q) and ranked by AICc/BIC. Residual adequacy was confirmed by Ljung–Box tests and inspection of residual ACF/PACF. When Engle–Granger tests supported a stable long-run relation with calendar time, we estimated an EC model with a lagged error-correction term. For each outcome (deaths, DALYs, YLLs, YLDs), we report the selected (p,d,q), AICc/BIC, and rolling-origin (2018–2021) RMSE/MAPE. Forecasts are summarized as medians with 80% and 95% prediction intervals.

Supplementary Results

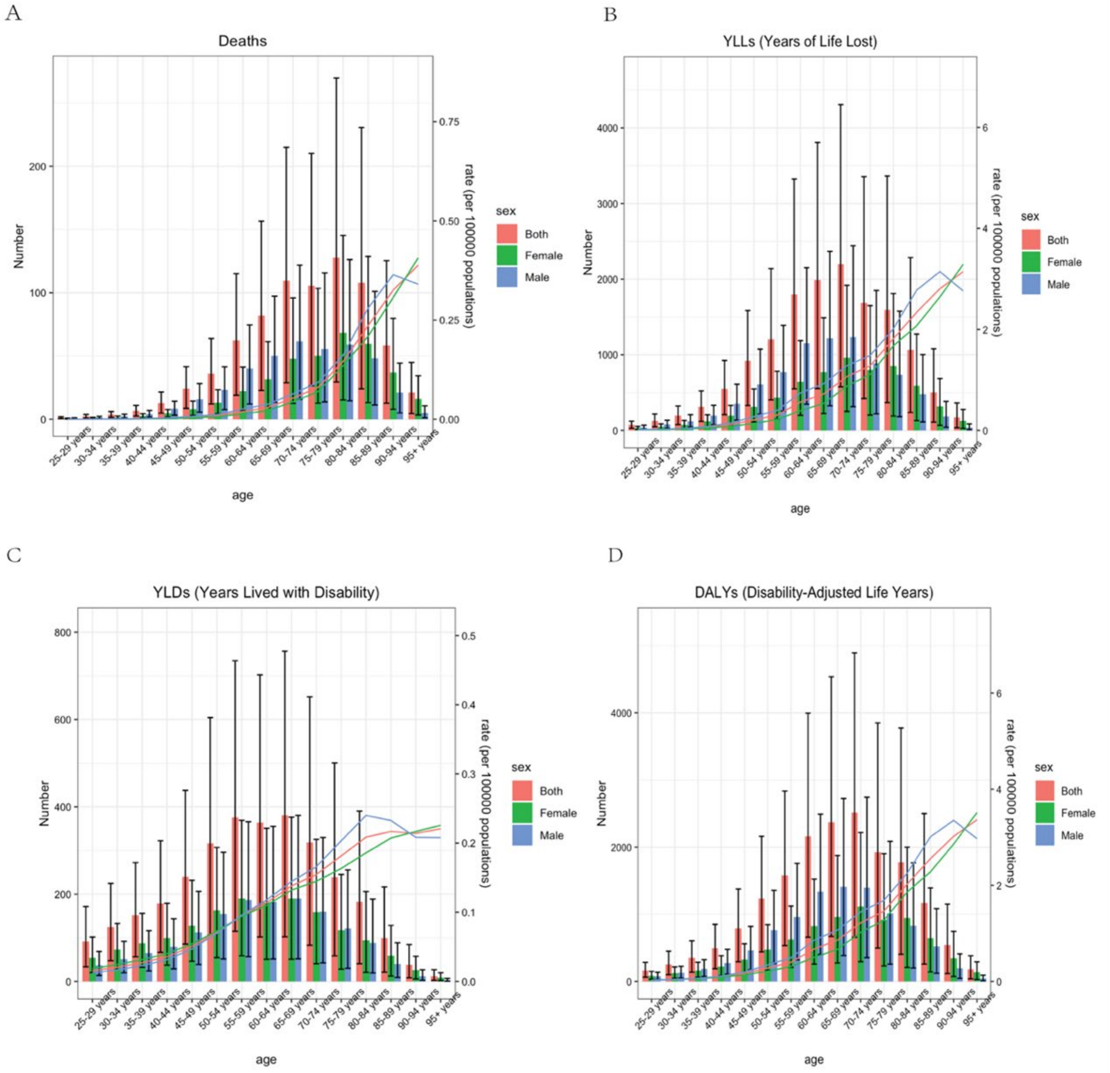

Supplementary Figure S1: Global, 2021 sex- and age-based contrasts in ischemic stroke burden attributable to diet low in PUFA (GBD 2021). Panels A–D correspond to Deaths (A), DALYs (B), YLLs (C), and YLDs (D). Within each panel, two descriptive comparisons are shown: women ≥50 vs men ≥50 and women ≥50 vs women <50. Bars denote absolute numbers (thousands); lines denote crude rates (per 100,000) with 95%

uncertainty intervals. (DALY, disability-adjusted life year; YLL, years of life lost; YLD, years lived with disability)

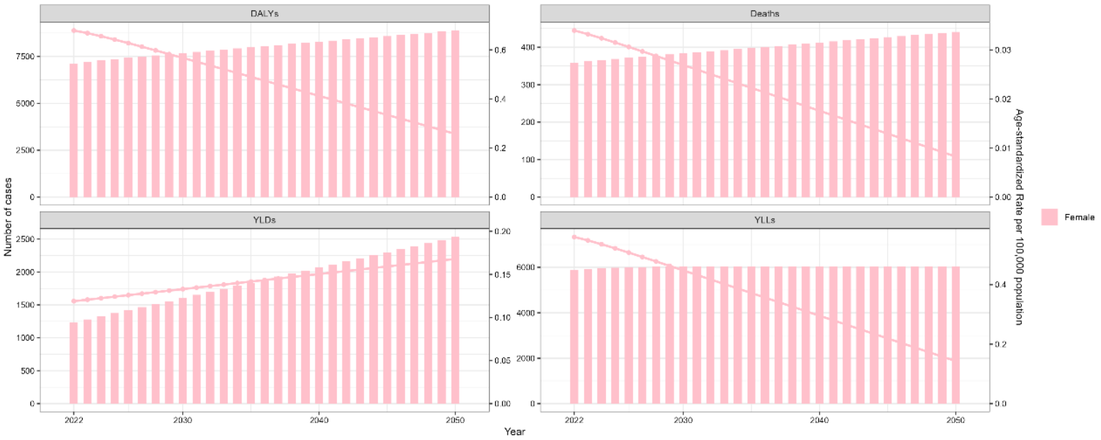

Supplementary Figure S2 (age–sex trajectories): 2022–2050 paths showing increasing YLD counts and declining ASRs across groups; within women, ≥50 years consistently exceed <50 years across outcomes; among adults ≥50, men generally show higher crude deaths and DALYs, while YLDs are similar between sexes.

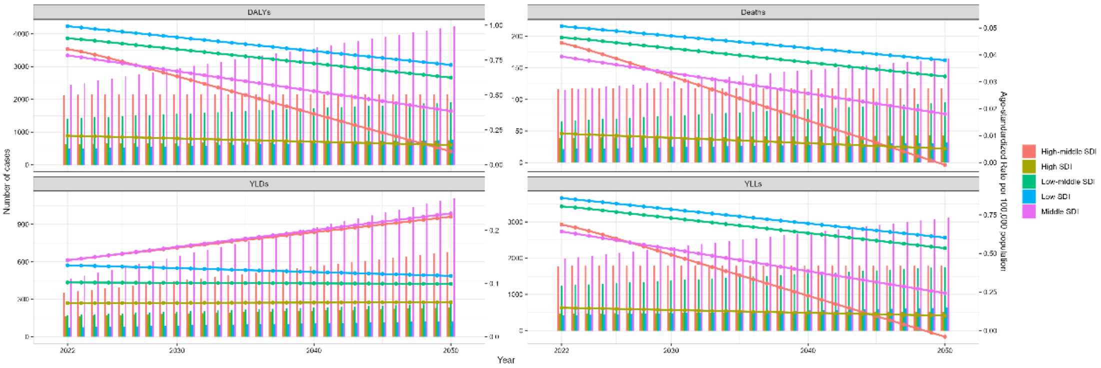

Supplementary Figure S3 (SDI-stratified projections): heterogeneous future burden; middle-SDI shows the steepest growth in counts despite declining ASRs.

A

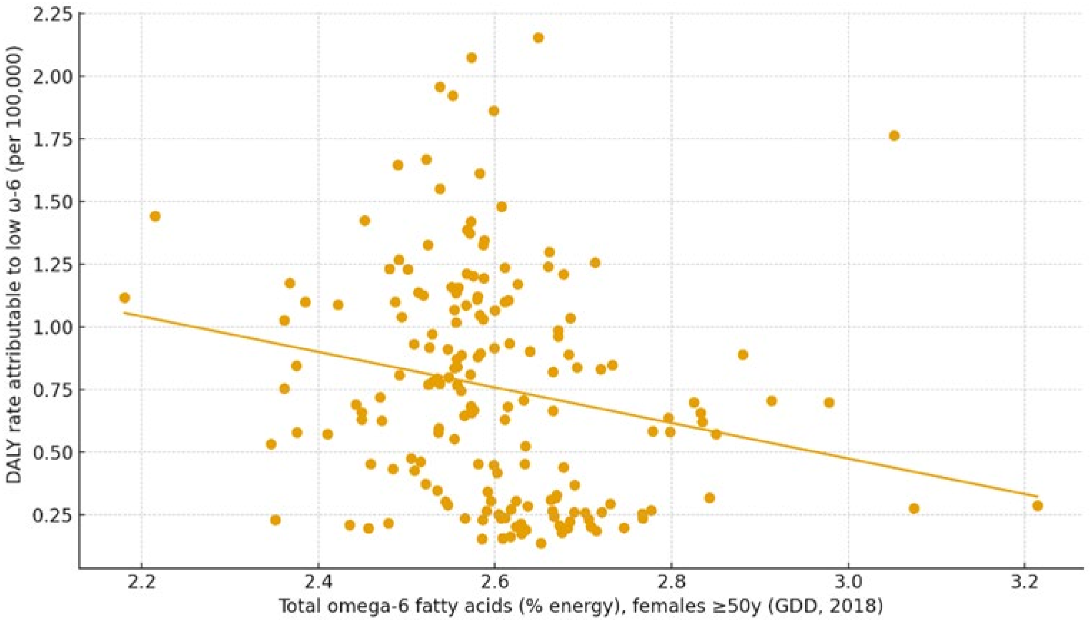

B

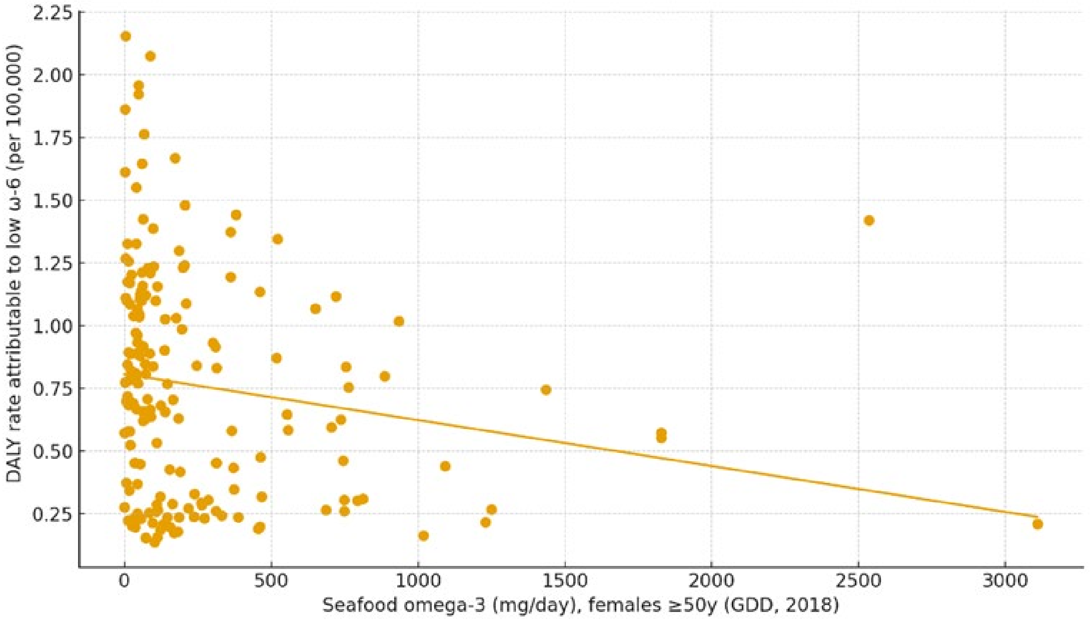

Supplementary Figure S4. External ecological validation combining dietary exposures from 2018 and ischemic stroke burden from 2021 (women ≥50 years). A. Country-level association between total omega-6 fatty acids (% of total energy; 2018) and ischemic stroke DALY rate attributable to diet low in omega-6 PUFA (per 100,000; 2021). B. Country-level association between seafood omega-3 fatty acids (mg/day; 2018) and the same attributable DALY rate (2021). Points denote countries; lines show least-squares fits. Abbreviations: GDD, Global Dietary Database; GBD, Global Burden of Disease; IS, ischemic stroke; DALY, disability-adjusted life-year; PUFA, polyunsaturated fatty acids; ω-6, omega-6 (n-6) fatty acids; ω-3, omega-3 (n-3) fatty acids.
